# Applying Unique Molecular Identifiers in Next Generation Sequencing Reveals a Constrained Viral Quasispecies Evolution under Cross-Reactive Antibody Pressure Targeting Long Alpha Helix of Hemagglutinin

**DOI:** 10.3390/v10040148

**Published:** 2018-03-25

**Authors:** Nastasja C. Hauck, Josiane Kirpach, Christina Kiefer, Sophie Farinelle, Sophie Maucourant, Stephen A. Morris, William Rosenberg, Feng Q. He, Claude P. Muller, I-Na Lu

**Affiliations:** 1 Department of Infection and Immunity, Luxembourg Institute of Health, 29, rue Henri Koch, L-4354 Esch-sur-Alzette, Luxembourg; Nastasja.Hauck@gmx.de (N.C.H.); josiane.kirpach@lih.lu (J.K.); muesli.chrissy.kiefer@googlemail.com (C.K.); sophie.farinelle@lih.lu (S.F.); feng.he@lih.lu (F.Q.H.); Claude.Muller@lih.lu (C.P.M.); 2 iQur Ltd., London NW1 0NH, UK; sophie.maucourant@touchlight.com (S.M.); stephen.morris@ucl.ac.uk (S.A.M.); william.rosenberg@iqur.com (W.R.); 3 Laboratoire national de santé, 1, rue Louis Rech, L-3555 Dudelange, Luxembourg

**Keywords:** influenza, next generation sequencing, unique molecular identifiers, virus evolution, immune selection, immune pressure, bottleneck effect, vaccine

## Abstract

To overcome yearly efforts and costs for the production of seasonal influenza vaccines, new approaches for the induction of broadly protective and long-lasting immune responses have been developed in the past decade. To warrant safety and efficacy of the emerging crossreactive vaccine candidates, it is critical to understand the evolution of influenza viruses in response to these new immune pressures. Here we applied unique molecular identifiers in next generation sequencing to analyze the evolution of influenza quasispecies under in vivo antibody pressure targeting the hemagglutinin (HA) long alpha helix (LAH). Our vaccine targeting LAH of hemagglutinin elicited significant seroconversion and protection against homologous and heterologous influenza virus strains in mice. The vaccine not only significantly reduced lung viral titers, but also induced a well-known bottleneck effect by decreasing virus diversity. In contrast to the classical bottleneck effect, here we showed a significant increase in the frequency of viruses with amino acid sequences identical to that of vaccine targeting LAH domain. No escape mutant emerged after vaccination. These results not only support the potential of a universal influenza vaccine targeting the conserved LAH domains, but also clearly demonstrate that the well-established bottleneck effect on viral quasispecies evolution does not necessarily generate escape mutants.

## 1. Introduction

Influenza A viruses (IAVs) remain a major public health concern, causing severe respiratory tract infections, especially in young children, the elderly, and patients with chronic and multiple morbidities. Seasonal epidemics can cause high mortality throughout the world [1]. Antiviral drugs for the treatment of influenza infections must be administered early during IAV infection and provide only partial reduction in disease severity. High levels of anti-drug resistance further undermine their application [2]. Therefore, vaccination is the most efficient and cost-effective public health intervention against IAV infections.

IAV vaccine strategies rely on a protective antibody response against the hemagglutinin (HA) and neuraminidase (NA). Both of these surface glycoproteins are highly variable among IAVs circulating in humans and animals [3]. Current vaccines induce only a narrow and strain-specific immunity. Because of antigenic drift and shift [4], they have to be reformulated for every season to match the circulating strains. It is therefore essential to improve current vaccines and to develop drift resistant strategies providing a broad and lasting protection. Such universal IAV vaccines are based on conserved domains of viral proteins. Usually these domains are less exposed to the host immune system and are less immunogenic resulting in less immune pressure-derived antigenic changes [1,5]. In contrast to the conserved influenza matrix protein and nucleoprotein epitopes, which provide only weak protection in human challenge studies [1], the conserved stalk portion of HA is a much more potent candidate for a universal vaccine [1,5,6]. The highly conserved long α-helix (LAH) of the stalk domain spanning amino acid (aa) 76–130 has been shown to induce broadly reactive and protective Ab responses [7,8,9].

Due to an error-prone replication machinery, IAVs can easily adapt to immune pressure. Therefore, it is of interest to monitor the emergence of escape mutants in particular against otherwise conserved epitopes [10,11,12,13]. In the present study, we used next generation sequencing (NGS) to investigate the impact of antibody mediated immune responses against LAH on strain diversity within the targeted region [7,8]. Unlike conventional techniques, NGS provides a direct and extensive snapshot of in vivo viral quasispecies [14,15,16]. Moreover, we incorporate unique molecular identifiers in our approach which has been previously shown to dramatically lower the sequencing error rate and to improve the sensitivity for detection of variants with very low frequencies [17,18]. Focusing on the LAH domains, we showed that there was a constrained evolution of viral quasispecies after vaccination and no emergence of a new major virus variant. These findings demonstrate that LAH is a promising candidate as a safe and potent universal influenza vaccine target.

## 2. Materials and Methods

### 2.1. Mice and Viral Infection

**Figure 1:**
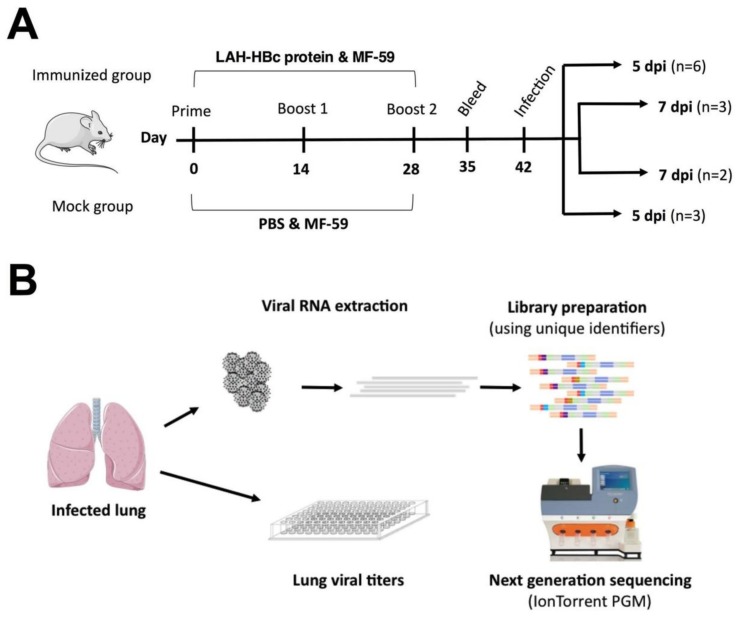
(**A**) Scheme of mouse immunization and virus challenge. (**B**) Assays performed on lung samples from immunized and mock immunized mice.

Female BALB/c mice were purchased from Harlan Laboratories, Inc. (Horst, The Netherlands). 8-week-old BALB/c mice were immunized 3 times intraperitoneally within 2 week intervals with LAH-HBc chimeric proteins containing LAH of pH1N1/09 (A/Luxembourg/46/2009, pH1N1) and MF59/AddaVax^TM^ (Invivogen, Toulouse, France). The LAH domain from pH1N1/09 has been incorporated into hepatitis B virus core protein (HBc) using recently developed tandem core technology [9]. The LAH-HBc chimeric proteins were produced in *E. coli* BL21 as described previously [19], and purified using two consecutive size exclusion chromatography steps. Mock group mice received adjuvant only. Two weeks after the final immunization, mice were challenged with 5 × 50% Mouse Lethal Dose (MLD50) of pH1N1/09 intranasally, after anesthesia with isoflurane [20]. Mice were sacrificed 5 days post infection (dpi) (*n* = 3 for Mock, *n* = 6 for Immunized) or 7 dpi (*n* = 2 for Mock, *n* = 3 for Immunized) for organ removal (Figure 1A). Animals with a 20% weight loss were sacrificed to conform with humane endpoint recommendations.

### 2.2. Ethics Statement

All mouse experiments were performed in accordance with protocols approved by the Animal Welfare Structure of Luxembourg Institute of Health and by the Minister of Agriculture, Viticulture and the Consumer Protection of the Grand Duchy of Luxembourg (Ref. LNSI-2014-02, permission date (1 September, 2014)).

### 2.3. ELISA

Sera from individual mice were collected 10 days after the final immunization and were tested for seroconversion. LAH-specific IgG in mouse serum were measured by indirect ELISA. A free polypeptide covering the entire sequence of the LAH region from pH1N1/09 virus (amino acids 76–130) was synthesized with a MultiPep RS peptide synthesizer (IntavisAG, Tuebingen, Germany) by a modified SPOT synthesis protocol. Wells of 384-well microtiter plates (Greiner, Diegem, Belgium) were coated overnight at 4 °C with 20 μL/well of 2.5 μg/mL resuspended LAH polypeptide or purified HA in carbonate buffer (100 mM, pH 9.6), or with carbonate buffer alone as a background control. All subsequent steps were performed at room temperature. Wells were washed sequentially in washing buffer (Tris containing 1% Tween 20) and blocked for 2 h with 1% BSA in Tris buffer. After washing, sera (starting 100-fold dilution) were added, incubated for 90 min, and washed. Bound IgG was detected using alkaline phosphatase (ALP) conjugated goat anti-mouse IgG (1/750 dilution, ImTec Diagnostics, Antwerp, Belgium). Color reactions were developed using 2-amino-2-methyle-1-propanale. Absorbance was measured at 405 nm (Spectromax Plus, Sopachem, Eke, Belgium). Purified HA for A/California/4/2009 (Cal09) (H1), A/Japan/305/57 (JP57) (H2), A/Perth/16/2009 (Perth09) (H3), A/Vietnam/1203/04 (VN04) (H5), A/Netherlands/219/2003 (Neth03) (H7), and A/Hong Kong/1073/99 (HK99) (H9) were purchased from Sino Biological Inc. (Beijing, China).

### 2.4. Virus Culture, Titrations and Lung Titer

Lungs (*n* = 14) were explanted and homogenized (TissueLyserII, Qiagen, Hilden, Germany) in 900 µL virus growth medium for 12 min at 25 Hz and centrifuged for 10 min at 11,000 rpm [21]. TCID50 in the supernatant was determined on Madin-Darby canine kidney (MDCK, American Type Culture Collection) cells. Wildtype pH1N1/09 virus was cultured in MDCK cells in serum free virus growth medium that contained 2 mg/mL l-1-tosylamido-2-phenylethyl chloromethylketone-(TPCK) trypsin (Sigma-Aldrich, Diegem, Belgium). 50% Tissue culture Infective Dose (TCID50) determinations of virus were performed on MDCK cells by incubating them in quadruplicates for 20 h with 8-fold serial dilutions of virus-containing supernatant at 37 °C and 5% CO_2_, and were calculated by the ID-50 5.0 program (http://www.ncbi.nlm.nih.gov/CBBresearch/Spouge/html_ncbi/html/index/software.html#1).

### 2.5. RNA Extraction and Library Preparation

RNA was extracted from wildtype virus and from the supernatant of the homogenized lungs using the QIAamp Viral RNA Mini kit (Qiagen, Hilden, Germany). The quality of the extracted RNA in the final eluent of 60 µL was analyzed employing an Agilent Bioanalyzer 2100 (Agilent Genomics, Santa Clara, CA, USA). The libraries for sequencing were prepared following an adapted protocol from Buerckert et al. (in preparation), Kinde et al. [17], Vollmers et al. [22], and Loman et al. [17,22,23] (Figure 3A). Reverse transcription was performed on 100–200 ng virus RNA (NanoDrop, Thermo Scientific, Waltham, MA, USA) with Superscript IV (ThermoFisher), following the provided protocol. The primer for reverse transcription (5′ CACAGTTCACAGCAGTAGGTAAAGA 3′) was linked to a unique identifier (UID) consisting of 14 random nucleotides which enables the recognition of every original mRNA strand after amplification. The primer was connected to four different mouse identifiers (MIDs) to allow pooling samples from different mice on a single sequencing chip. Furthermore, the primer contained a short sequence of IonTorrent A-Adaptor. After the reverse transcription the second strand synthesis was performed with Phusion enzyme (NewEngland BioLabs, Ipswich, MA, USA) on half the outcome of reverse transcription. The reverse primer (5′ ATTGCCCCCAGGGAGACTAC 3′) was connected to a short sequence of IonTorrent P1-Adaptor (2′98°, 2′50°, 10′72°). After second strand synthesis and before amplification, the samples were purified twice with Agencourt AMPure XP beads (Beckman Coulter, Suarlée, Belgium) at a ratio of 1:1. For the final amplification step of the library Q5 enzyme (NewEngland BioLabs, Ipswich, Massachusetts) in combination with a primer mix of A-Adaptor and P1-Adaptor was used (5′98°, 20× (10′′98°, 20′′65°, 30′′72°), 2′72°). The finished library was purified once with Agencourt AMPure XP beads before being analyzed for both quality and quantity on Agilent Genomic’s Bioanalyzer 2100 (High sensitivity chip) before deep sequencing. The amount of library input RNA for NGS was determined by the reciprocal library output quality of 150 fluorescence units on Agilent Genomic’s Bioanalyzer 2100 (Table S1). For each sample, libraries with different amounts of RNA input were prepared. Those libraries that showed similar end concentrations were chosen to be sequenced. This approach allowed us to control a similar RNA input between different samples. In combination with UID barcoding, this allow for the exclusion of a possible effect of differences in viral titers on the rate of false positive variant calls between the groups [18].

### 2.6. High-Throughput Next Generation Sequencing

Libraries of two immunized and one mock mouse were pooled to be sequenced on the same chip. Wildtype virus libraries were done in triplicates and pooled together. Template preparation was done using the Ion PGM™ Template OT2 400 Kit (Life Technologies Europe BV, Gent, Belgium). For sequencing, the Ion PGM™ Sequencing 400 Kit (Life Technologies Europe BV, Gent, Belgium) and Ion 316/318 Chip Kits v2 (Life Technologies Europe BV, Gent, Belgium) were used. For all these steps protocols were followed as indicated by LifeTech (Paisley, UK).

### 2.7. NGS Data Analysis

Trimmed BAM files were exported from the IonTorrent platform. An in-house pipeline adapted to the IonTorrent technology was built to filter out poor quality reads and to correct for homopolymer errors (Figure 3B and refer to the details below). In order to normalize for differences between sequencing runs, similar to the principle of quantile normalization [17,24], we compared the percentage of reads with a minimum coverage of 3 (it was chosen because consensus building requires at least 3 copies) for every chip. A minimum UID copy number for a sequence to be included into data analysis per chip was calculated, so that the same percentage of reads (33%) from every chip would be used. The threshold of 33% was determined by checking for every chip the percentage of reads with a copy number greater or equal to 3 and then selecting the lowest percentage of all chips. To build a reliable consensus sequence from only 3 copies for a given UID, a minimum of 2 need to be the same to define a reliable nucleotide read. The thresholds were calculated individually for every degree of coverage using the cumulative binomial distribution, with q = 0.015625, and α = 1 − q. Cumulative binomial distribution was used to create a list that assigns the copy number needed to always reach the same probability to every possible copy number/UID.

Trimmed BAM files exported from IonTorrent platform were imported. In a first quality filtering step, reads with 20% less than the expected length were filtered out and only reads which had more than 95% of nucleotide positions with a minimum quality score of 20 were kept for further analysis. Then, after splitting the reads from the different samples according to their MIDs, they were grouped into reads originally coming from the same mRNA template input by using the UIDs. Errors in poly-A-sequences were corrected to forestall the most commonly reported indel errors in IonTorrent sequencing by replacing homopolymeric regions with the correct number of nucleotides (as in the virus reference sequence) [25,26]. Sequences were cut to include only the epitope (corresponding to HA amino acids 420–474) that the vaccine construct targets. Within each selected UID family (Figure 3B), we aligned reads using MAFFT (a multiple sequence alignment program) and built a consensus sequence using Biopython (gap_consensus). Geneious and Bioedit were used to convert the nucleotide sequences into amino acid sequences and to study mutations. The Shannon Diversity index was calculated [25] to determine diversity of quasispecies population. All sequences, including the ones having frameshifts or stop codons (1–2% of final sequences) were included in the diversity calculations. The heat map showing each diversified amino acid position on the LAH epitope was generated using R program. Values for each mutation have first been normalized by determining the percentage of total consensus sequences they make up in the individual samples before a clustering analysis. An overview of the sample properties for NGS is shown in Table S1. Details of amino acid sequences in each sample are listed in Tables S2 and S3.

### 2.8. Statistical Analysis

In GraphPad Prism 5 (San Diego, CA, USA) multiple *t*-tests, unpaired *t*-test, one-way ANOVA followed by Tukey’s as post-hoc test and Benjamini and Hochberg correction were used to determine statistical significance. A *p* value less than 0.05 was considered as significant.

## 3. Results

### 3.1. Induction of Broadly Reactive Anti-LAH Antibodies by Immunization

**Figure 2:**
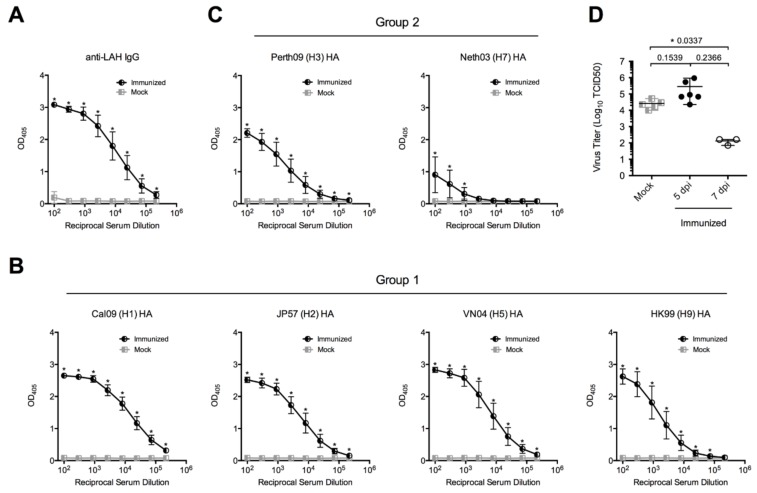
Seroconversion elicited by vaccination. Serum reactivity with synthetic long alpha helix (LAH) peptide (**A**), with group 1 HA proteins H1, H2, H5 and H9 (**B**) and with group 2 HA proteins H3 and H7 (**C**). (**D**) Lung virus titers of mice five (5 dpi) and seven (7 dpi) days post infection. * *p* < 0.05. Error bars represent standard error of the mean (SEM).

To elicit antibodies against the influenza virus LAH domain, BALB/c mice were immunized three times with LAH-HBc chimeric proteins at two week intervals (Figure 1A). Seven days after the third immunization, all immunized animals showed high titers of antibodies against a synthetic LAH peptide, while the mock group showed no reactivity (Figure 2A). The LAH antisera reacted not only with the homologous H1 HA protein but cross-reacted also with multiple group 1 and group 2 HA proteins, including H2, H3, H5, H7, and H9 (Figure 2B,C). Thus, LAH-HBc chimeric proteins induced broadly reactive anti-LAH serum against both group 1 and 2 HA proteins.

### 3.2. Reduced Lung Virus Titers in Immunized Mice

After immunization, animals were challenged intranasally with 5× MLD50 of IAV and the viral load was measured in the lung at five and seven days post infection (dpi). A significant difference in virus lung titers emerged at 7 dpi between immunized and mock animals (Figure 2D). In parallel, another group of animals receiving the same immunization and viral challenge were maintained for survival observation. As shown in Figure S1, LAH-HBc chimeric protein immunized mice were significantly protected against lethal challenge with pH1N1 and H3N2 viruses while in the mock group all animals died.

### 3.3. Decreased Diversity of LAH Epitopes

**Figure 3:**
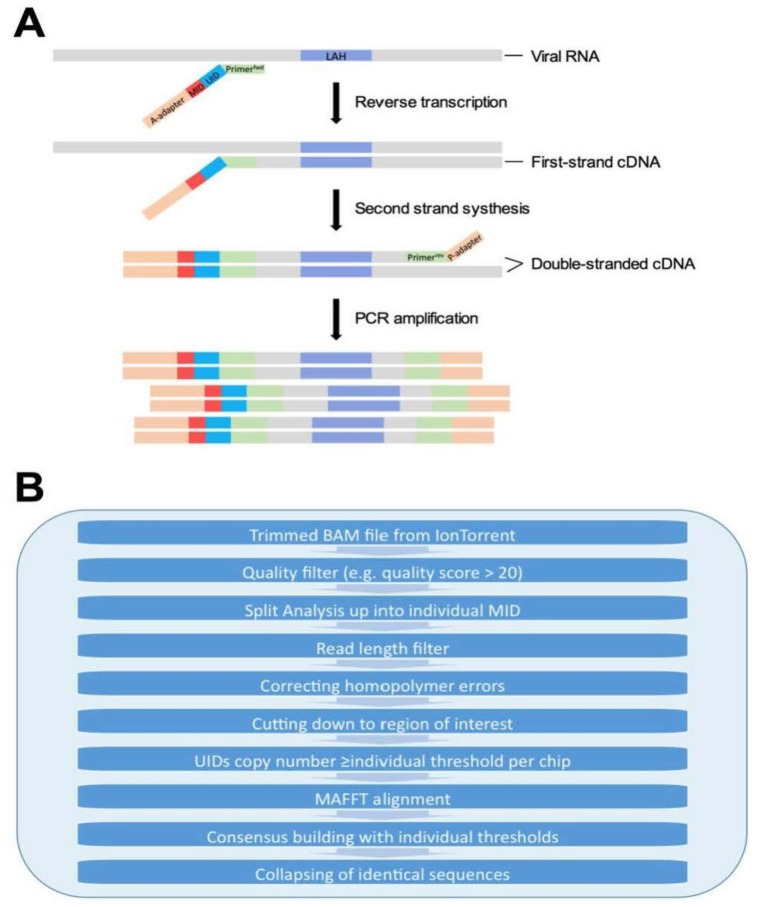
Overview of (**A**) library preparation using unique identifiers (UID) and (**B**) processing pipeline used for deep sequencing data cleanup.

**Figure 4:**
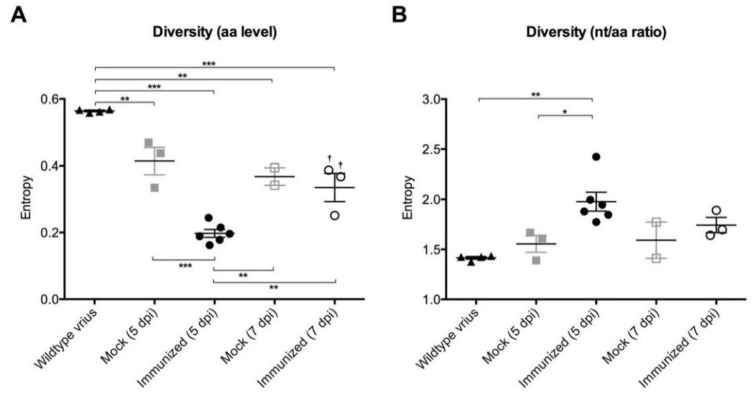
Lung viruses of vaccinated mice showed a reduction in LAH sequence diversity. (**A**) Shannon Diversity index of LAH amino acids. †: corresponding to the samples with the same labeling in Figure 5B and Figure S2A. (**B**) Nucleotide to amino acid entropy ratios of the Shannon Diversity calculated for each sample. * *p* < 0.05, ** *p* < 0.01, and *** *p* < 0.001. Error bars represent SEM.

We further characterized the virus on a population level at 5 dpi and 7 dpi using NGS (Figure 1B and Figure 3). The overall diversity of viral quasispecies in the LAH domain in the different groups was calculated using the Shannon Diversity Index. The wildtype virus expanded in vitro in MDCK cells showed the highest variability within the tested region (Figure 4A). At 5 dpi, viruses from the lung of immunized mice showed significantly less variability than those of the mock group. This was no longer true at 7 dpi (Figure 4A). These results demonstrate that in the animals the virus loses diversity and that this effect is more dramatic in immunized mice than in the control group. Inversely, when comparing the diversity of nucleotides with amino acids, the ratio was significantly increased at 5 dpi in the immunized mice compared to the wildtype virus and mock animals (Figure 4B). Thus, in the immunized animals the virus has more synonymous mutations compared to the one from the wildtype virus and the mock group.

### 3.4. Absence of Escape Mutants Following Vaccination

**Figure 5:**
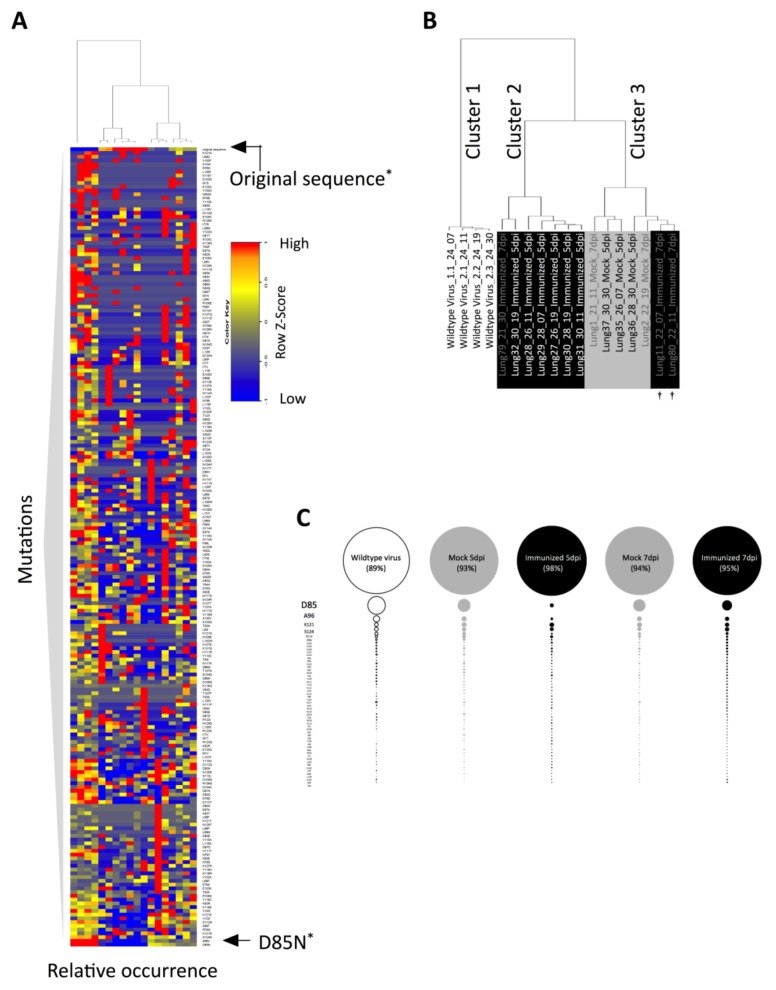
Constrained viral quasispecies evolution under immune pressure. (**A**) Heatmap and dendrogram of missense mutations detected in at least three out of the 18 samples analyzed by Z score based on frequency. Values from each row (i.e., mutation) have been normalized to have a mean of 0 and SD of 1. Relative expression levels of the same mutation within the individual mice are represented by the color code indicated. (**B**) Enlarged dendrogram from (**A**) using hierarchical clustering analysis performed by complete linkage and Euclidean distance measurement. †: corresponding to the samples with the same labeling in Figure 4A and Figure S2A; (**C**) Mean sequence composition of samples per group. The sizes of the bubbles are proportional to the mean percentage sample consensus sequences of each group. * *p* < 0.05. Error bars represent SEM.

To understand the observed differences in quasispecies diversity, we examined the missense mutations in the different groups. In order to select relevant mutations only, we focused on those that appeared in at least 3 of the 18 samples. With this approach we found 212 possible amino acid exchanges within the analyzed epitope, which covered all 55 positions. A heat map was generated to compare the occurrence of these missense mutations between the groups (Figure 5A). Hierarchical clustering based on these occurrence levels resulted in three major clusters, one composed only of wildtype virus samples (cluster 1), a second one composed only of samples from immunized mice (cluster 2), and a third one containing all mock samples and two of the 7 dpi samples from the immunized group (cluster 3) (Figure 5B). This observation could be further confirmed by principle component analysis (PCA) to the frequencies of mutants among different groups (Figure S2A). The marked two 7 dpi samples showed a similar level of diversity as in the mock mice (Figure 4A and Figure S2A). An overall gradual decline in mutated virus sequences measured at 5 dpi can be observed when comparing the wildtype virus, mock, and immunized samples (Figure 5C), confirming again the differences in diversity observed between the groups (Figure 4A).

### 3.5. Identification of Diversified Positions on LAH Epitope

**Figure 6:**
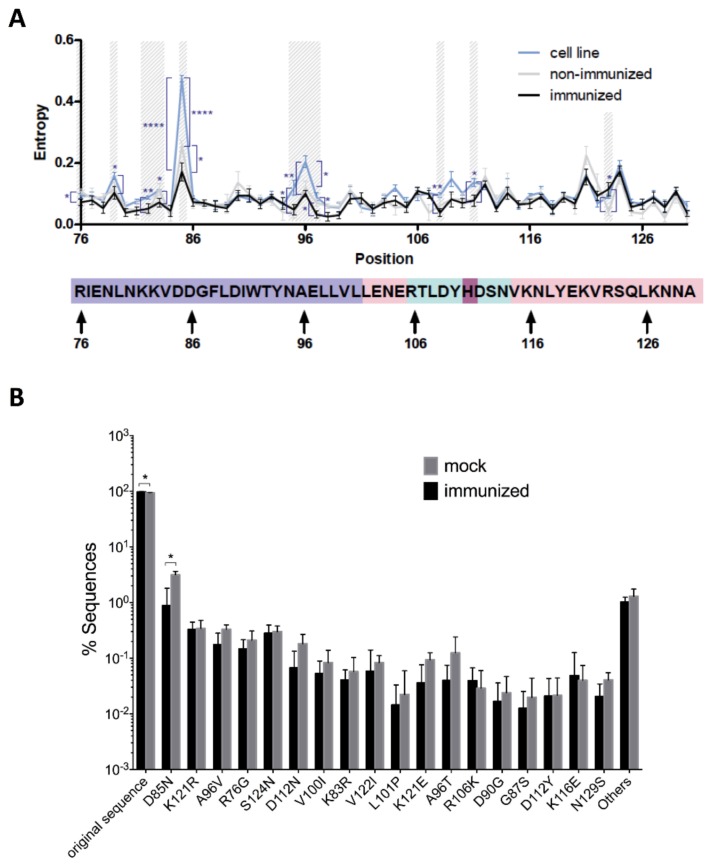
Identification of variable amino acid positions. (**A**) Entropy at individual positions. Significant differences of mutation frequencies among groups are highlighted. * *p* < 0.05, ** *p* < 0.01, and **** *p* < 0.0001. *p*-values were calculated before the Benjamini and Hochberg correction. (**B**) Frequencies of top 18 mutations in Mock and Immunized groups. * *p* < 0.05 after the Benjamini and Hochberg correction. Error bars represent SEM.

In order to extract the most significantly mutated positions of the analyzed epitope, the Shannon Diversity index was calculated for every amino acid position across the LAH epitope. In this way, 11 different positions were identified to reach statistical significance when comparing the groups using a two-tailed student’s *t*-test (Figure 6A). At each of these positions, except for position 123, the immunized group showed a lower or similar aa diversity as compared to the mock mice. After Benjamini and Hochberg correction, the diversity in amino acid positions 85 and 96 was still significantly higher in the cultured virus than that in the in vivo virus populations. Variability in these two positions were the major contributors to the differences in diversity observed between the groups (Figure 4A) as confirmed by PCA analysis on the frequency of mutants (Figure S2A). Positions 121 and 124 seem to be generally more diverse but with little differences among groups (Figure 6A). The PCA analysis on the Shannon Diversity per position showed differences between in vitro (Wildtype Virus) and in vivo (Mock and Immunized) samples (Figure S2B). However, the differences between Mock and Immunized mice were not clearly distinguishable by using the Shannon Diversity index alone (Figure S2B), indicating the need for more detailed analysis on the amino acid substitutions.

### 3.6. Analysis of Amino Acid Substitutions

To identify the mutations that were selected by the vaccine-induced immune pressure, all the identified 212 amino acid exchanges were compared between the Immunized and Mock groups. Notably, the frequencies of the mutation D85N retained significant difference between the two groups following the Benjamini and Hochberg correction. The mutation D85N has an average number of reads of 97.8/sample (average frequencies: 0.86%) in the immunized group vs. 474.2/sample (average frequencies: 3.09%) in the control group (Figure 6A,B). This amino acid showed a significantly reduced frequency in the immunized animals (Figure 6B). No mutant variant emerged that replaced the dominant viral sequence in any of the samples. There were, however, significant differences in the occurrence of the dominant viral sequence between groups (Mock: 93.70 ± 1.01%; Immunized: 96.76 ± 1.40%; *p* = 0.01 after the Benjamini and Hochberg correction, Figure 6B), suggesting a constrained viral quasispecies evolution in the immunized group.

## 4. Discussion

In the present study, we applied NGS to follow the in vivo evolution of IAV quasispecies in response to our novel HBc-based LAH fusion protein. This new LAH targeting vaccine induced a strong antibody response and protected against both homologous (pH1N1) and heterologous IAV (H3N2) strains in mice. Interestingly, after vaccination, the virus quasispecies showed a significantly reduced variability in amino acid sequences of the LAH domain and no escape mutant emerged.

The analysis of quasispecies is complicated by the need to reduce technical nucleotide substitution artefacts to a minimum. Therefore, we chose the IonTorrent platform which in contrast to other NGS technologies has a low propensity for substitution errors (0.085/100 bp) [27]. On the other hand, this platform is prone to indel errors (0.6/100 bp), which, however, can be reliably removed when the corresponding reads are compared with a reference sequence. To further reduce substitution errors, we applied a UID barcoding technique that allows to trace back individual RNA strands [17,23]. This technique also enabled us to pool samples from different groups on a single sequencing chip to minimize differences caused, for example, by chip loading. Inter-chip normalization further improved the comparability of different samples measured on different chips [28]. Compared with the other NGS approaches, Kinde et al. [17] and Peng et al. [18] have reported a dramatic reduction in the sequencing error of about 20 folds with a tagging method that is based on labeling nucleotide fragments with primers containing 14-bp UIDs. This UID approach allows for detecting as low as 0.001% mutations per base pair and has the ability to detect 1% mutations with minimal false positives. Although our sequencing approach covered the complete LAH, like most of the other NGS techniques it does not allow to reconstitute longer sequences (e.g., of the whole HA), in particular when the variability is low. Because of this limitation it would not have been possible to link mutations within LAH with other concurrent HA mutations. Sequencing the whole HA would have provided information about possible escape mutations outside of the targeted epitope region, but current NGS techniques do generally not allow to constitute the genome on a single strand so that mutations that occur on different amplicons cannot be related to each other in a straightforward way.

We used a mouse infection model to monitor the changes of lung virus quasispecies after vaccination against LAH, i.e., under the pressure of protective LAH antibodies. Unvaccinated animals failed to control IAV replication in the lungs and died at 7 dpi due to pulmonary damage. In response to the LAH vaccine, viral replication in the lungs became detectable at 3 dpi, peaked at day five, and began to decrease at day seven. This is in line with previous observations that a short but rapid virus proliferation in mice with a stronger immunity leads to enhanced activation of the acquired immune system, which ultimately results in rapid virus clearance [29,30,31]. Using Fcγ chain–deficient mice, DiLillo et al. [32] has shown the mice that received the broadly neutralizing HA stalk–specific antibodies treatment showed minimal weight loss compared to PBS-treated mice at day 7 and such an effect requires FcγR interactions for protection against influenza virus in vivo. Thus, our observation on the significant reduction of lung viral titer at day 7 might be associated with the effect of virus-specific cytotoxicity mediated by LAH–specific antibodies. A bottleneck effect [16,32,33,34], i.e., a reduction in viral diversity concomitant with emerging escape mutants, is often observed in viral evolution studies under host immune selection pressure in humans. Here we also indeed observed a reduced diversity in the LAH domain at 5 dpi, but unexpectedly, without any emerging escape mutant. Our analysis showed that, in the immunized animals at 5 dpi, the virus has more synonymous mutations. This is indicative of an ongoing adaptation process in these mice which generates higher genetic diversity with higher mutation rates in nucleotide sequences albeit under structural or functional constraints [35]. It looks like the virus tried to generate a higher genetic diversity, but only mutant variants without changes at the amino acid level were able to survive. Our observation that mutations are unfavorable in LAH domain may be due to their important role for the conformational changes of HA that are induced by low pH during fusion between the viral envelope and the host endosome membrane.

We observed one mutation that showed a conspicuous and significant difference in prevalence between the immunized and the mock group: D85N in the heptad repeat domain [36] was significantly lower in the immunized group. In addition, previous studies have shown that it is common in nature for influenza virus variants to possess shorter length of genes due to premature termination [37,38,39]. Indeed, we observed 1–2% of final sequences containing frameshifts or stop codons. Furthermore, we also found an unexpected preferential proliferation of viruses with non-mutated LAH in the immunized mice. This suggests that the virus failed to generate mutations in the LAH domain that were compatible with virus fitness. The absence of major non-synonymous substitutions within this region indicates that the amino acid composition is highly restricted, leaving little room for escape mutants with sufficient viral fitness to develop. This is in line with the extensive conformational changes that the LAH undergoes during viral proliferation [36]. Consistent with our notion, Chai et al. [40] have reported that under the very focused pressure of a protective monoclonal antibody against LAH, escape mutants can develop in vitro, but these suffer from a reduced fitness both in vitro and in vivo. Although mutant variants increased again in proportion at the later timepoint, no new mutant variant dominated the virus population. The proportional increase in mutant variants from 5 dpi to 7 dpi in the vaccinated group could be explained by the observed decrease in total virus titers at the later stage (Figure 2D). Another possible explanation for this phenomenon could be an unequal pressure on the different mutant variants as a result of a decline in the broadly reactive CTL response [41,42].

Other mutations may have occurred in other regions of HA outside of the targeted LAH domain, that may protect the virus against LAH antibodies. In our approach, we focused first on the targeted epitope as proof of concept. Synergetic mutations using this approach cannot be detected and further studies sequencing the whole genome to confirm and extend our findings will be of interest. Moreover, because of the above difficulties to link them to LAH mutations, it would have been challenging to interpret these. The conventional seasonal split vaccine induces a much larger panoply of protective antibodies against the HA head [24] and none against the HA stalk or the LAH domain [1]. Similarly, the seasonal vaccine is also not known to favor mutations in this domain. In any case, at 7 dpi animals immunized with the split vaccine have essentially cleared the challenge virus, further complicating any comparison between the split and the LAH vaccine. Therefore, in this work we did not include a split vaccine as an additional control. In the future, universal vaccines will have to be judged in comparison to seasonal vaccines so further studies are needed to determine similarities and differences.

Altogether, we observed a bottleneck effect in terms of variability [16] and no increase in sequence diversity, nor the displacement of the dominant virus strain after vaccination against LAH or the development of an escape mutant. These findings provide further support for LAH as a potential vaccine candidate and as proof of concept for a broadly protective influenza vaccine without enhanced propensity for escape mutants.

## Supplementary Materials

The following are available online at http://www.mdpi.com/1999-4915/10/4/148/s1, Figure S1: Protection conferred against lethal challenge of 5xMLD50 of either pH1N1 (A/Luxembourg/46/2009) or H3N2 (A/Aichi/68) virus, Figure S2: Principle component analysis, Table S1: Sample characteristics, Table S2: Comparison of diversity at individual epitope positions, Table S3: Table containing the different amino acid sequences of LAH domain (55 amino acids) and the number of sequences within the individual samples.
