# Supplementary material for: Applying Unique Molecular Identifiers in Next Generation Sequencing Reveals a Constrained Viral Quasispecies Evolution under Cross-Reactive Antibody Pressure Targeting Long Alpha Helix of Hemagglutinin
Source: Viruses. 2018 Mar 25;10(4):148. doi: 10.3390/v10040148 (PMC5923442; doi:10.3390/v10040148)
Supplement: Supplementary file 1 [file viruses-10-00148-s001.pdf]

**Supplementary information**

**Figure S1:** Protection conferred against lethal challenge of 5x MLD50 of either pH1N1 (A/Luxembourg/46/2009) or H3N2 (A/Aichi/68) virus. Mock mice were vaccinated with adjuvant only. Mice vaccinated with LAH-HBc chimeric proteins showed (A and B) 100% survival after pH1N1 challenge and (C and D) 75% survival after H3N2 challenge, while the mock mice all died. Error bar indicates SD.

**Figure S2:** (A) Principle component analysis on the frequency of mutants. †: corresponding to the samples with the same labeling in Fig. 4A and 5B. (B) Principle component analysis on the Shannon diversity per position.

**Table S1:** Sample characteristics.

**Table S2:** Comparison of diversity at individual epitope positions. Highlighted in grey are the p-values that are smaller or equal to 0.05.

**Table S3:** Table containing the different amino acid sequences of LAH domain (55 amino acids) and the number of sequences within the individual samples.

# Figure S1

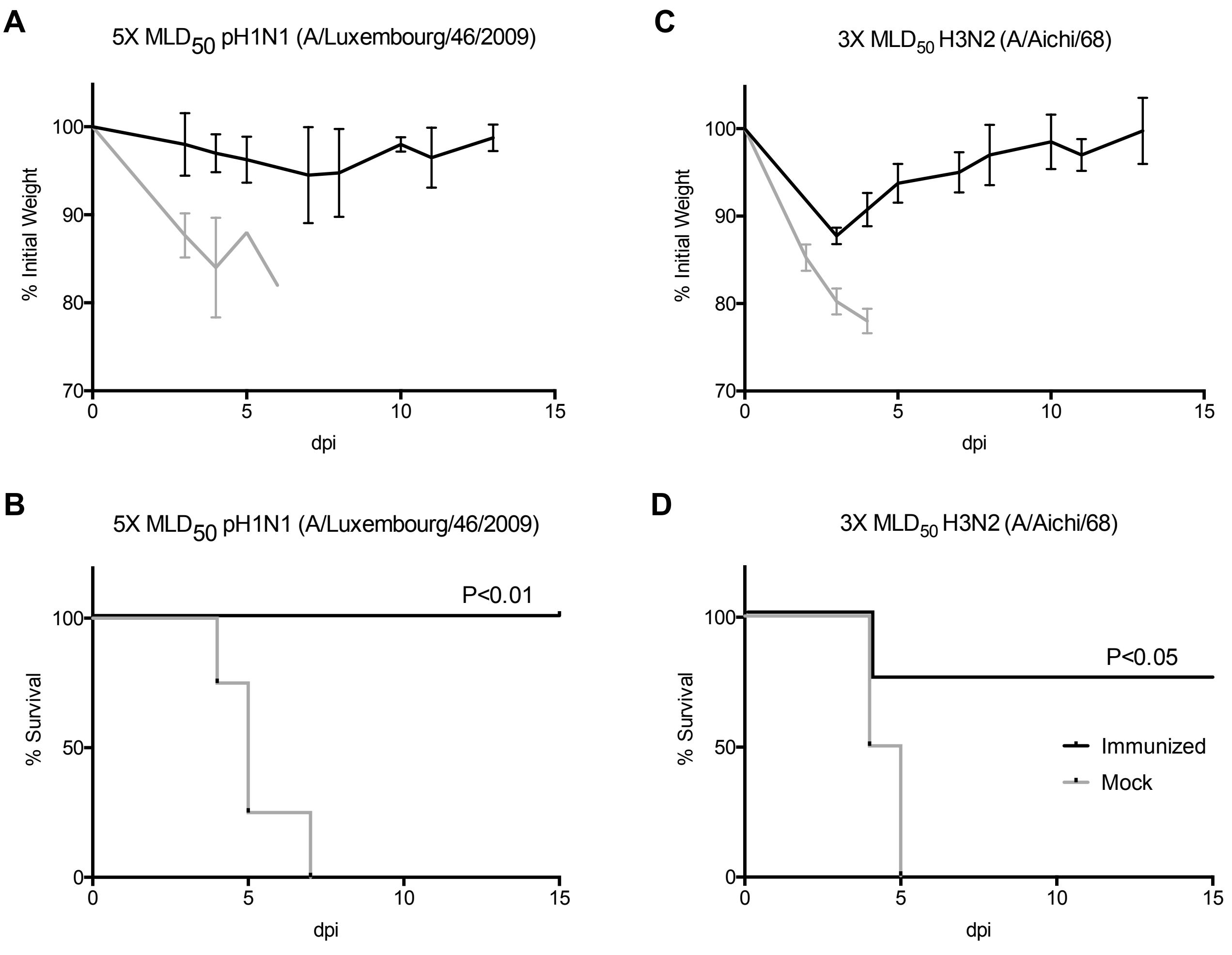
A
5X MLD50 pH1N1 (A/Luxembourg/46/2009)
% Initial Weight
dpi
B
5X MLD50 pH1N1 (A/Luxembourg/46/2009)
P<0.01
% Survival
dpi
C
3X MLD50 H3N2 (A/Aichi/68)
% Initial Weight
dpi
D
3X MLD50 H3N2 (A/Aichi/68)
P<0.05
% Survival
Immunized
Mock
dpi

**Figure S2**

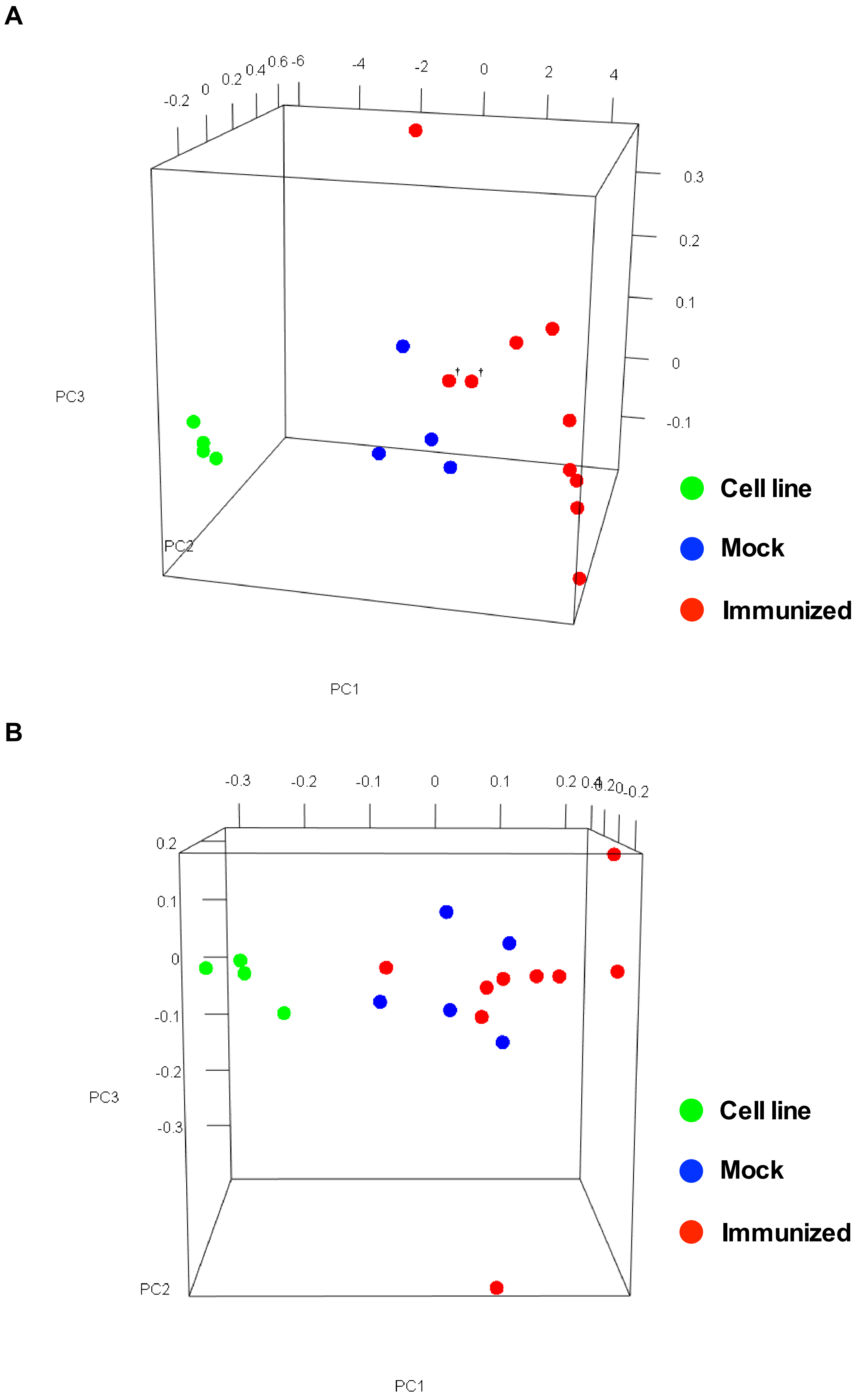
A
-0.2
0
0.2
0.4
0.6
-6
-4
-2
0
2
4
0.3
0.2
0.1
0
-0.1
PC3
PC2
PC1
Cell line
Mock
Immunized
B
-0.3
-0.2
-0.1
0
0.1
0.2
0.2
0.1
0
-0.1
-0.2
-0.3
PC3
PC2
PC1
Cell line
Mock
Immunized

**Table S1** : Sample characteristics

| Sample ID | Sample type | Library input RNA (ng) | Sacrife DPI | Total entropy (nt) | Total entropy (aa) | Lung viral load (TCID50/ml) | Raw reads | UIDs | Reads for consensus sequence | Consensus sequences | Average reads/UID | Unique consensus sequences | Average UIDs/unique consensus sequence | Average reads/unique consensus sequence | Pool N° | MID |
|---|---|---|---|---|---|---|---|---|---|---|---|---|---|---|---|---|
| 1,1 | Virus stock (wildtype virus) | 100 | n.a. | 0,768456 | 0,55844187 | n.a. | 249062 | 28895 | 207057 | 19306 | 10,725008 | 319 | 60,52037618 | 649,0815047 | 24 | 7 |
| 2,1 | Virus stock (wildtype virus) | 100 | n.a. | 0,805674 | 0,56186719 | n.a. | 413227 | 62346 | 377514 | 46755 | 8,0743022 | 539 | 86,74397032 | 700,3970315 | 24 | 11 |
| 2,2 | Virus stock (wildtype virus) | 100 | n.a. | 0,808001 | 0,567291049 | n.a. | 384512 | 51099 | 351825 | 38826 | 9,0615824 | 501 | 77,49700599 | 702,245509 | 24 | 19 |
| 2,3 | Virus stock (wildtype virus) | 100 | n.a. | 0,810224 | 0,56844083 | n.a. | 478384 | 75522 | 434946 | 51322 | 8,4748451 | 572 | 89,72377622 | 760,3951049 | 24 | 30 |
| 1 | Lung (non-immunized) | 275 | 7 | 0,555852 | 0,394090453 | 1,17E+04 | 389408 | 26892 | 218449 | 8102 | 26,962355 | 147 | 55,11564626 | 1486,047619 | 21 | 11 |
| 2 | Lung (non-immunized) | 1375 | 7 | 0,606014 | 0,34190796 | 2,97E+04 | 968277 | 249635 | 418190 | 55685 | 7,5099219 | 537 | 103,6964618 | 778,7523277 | 22 | 19 |
| 35 | Lung (non-immunized) | 145 | 5 | 0,783145 | 0,46964991 | 5,18E+04 | 520478 | 17578 | 468695 | 11755 | 39,871969 | 298 | 39,44630872 | 1572,802013 | 26 | 7 |
| 36 | Lung (non-immunized) | 100 | 5 | 0,538169 | 0,334547876 | 2,26E+04 | 729269 | 34345 | 697904 | 13131 | 53,149341 | 199 | 65,98492462 | 3507,055276 | 28 | 30 |
| 37 | Lung (non-immunized) | 200 | 5 | 0,607578 | 0,437185145 | 1,01E+04 | 504915 | 18283 | 434872 | 1852 | 234,8121 | 69 | 26,84057971 | 6302,492754 | 30 | 30 |
| 79 | Lung (immunized) | 1750 | 7 | 0,475210 | 0,251287634 | 1,62E+02 | 359130 | 12425 | 327526 | 5014 | 65,322298 | 128 | 39,171875 | 2558,796875 | 21 | 30 |
| 80 | Lung (immunized) | 640 | 7 | 0,602794 | 0,367450867 | 1,62E+02 | 558652 | 13859 | 539592 | 9040 | 59,689381 | 201 | 44,97512438 | 2684,537313 | 22 | 11 |
| 11 | Lung (immunized) | 600 | 7 | 0,657133 | 0,387058732 | 7,12E+01 | 405625 | 18700 | 391768 | 14014 | 27,955473 | 284 | 49,34507042 | 1379,464789 | 22 | 7 |
| 27 | Lung (immunized) | 110 | 5 | 0,368877 | 0,196255209 | 8,97E+04 | 604422 | 15195 | 583742 | 10267 | 56,856141 | 137 | 74,94160584 | 4260,890511 | 26 | 19 |
| 28 | Lung (immunized) | 100 | 5 | 0,392637 | 0,162089965 | 6,82E+04 | 848061 | 95931 | 334904 | 21357 | 15,681229 | 239 | 89,35983264 | 1401,271967 | 26 | 11 |
| 29 | Lung (immunized) | 100 | 5 | 0,396898 | 0,215045413 | 6,82E+04 | 397063 | 27641 | 386600 | 23130 | 16,714224 | 253 | 91,4229249 | 1528,063241 | 28 | 7 |
| 30 | Lung (immunized) | 100 | 5 | 0,367257 | 0,188697023 | 5,40E+05 | 620031 | 40259 | 601034 | 31871 | 18,858335 | 303 | 105,1848185 | 1983,610561 | 28 | 19 |
| 31 | Lung (immunized) | 100 | 5 | 0,355671 | 0,178209204 | 2,26E+04 | 780964 | 8348 | 742711 | 3718 | 199,76089 | 83 | 44,79518072 | 8948,325301 | 30 | 11 |
| 32 | Lung (immunized) | 100 | 5 | 0,432963 | 0,244150093 | 9,36E+05 | 678494 | 19510 | 512667 | 7991 | 64,15555 | 153 | 52,22875817 | 3350,764706 | 30 | 19 |

## Table S2

| Position | 76 | 77 | 78 | 79 | 80 | 81 | 82 | 83 | 84 | 85 |
|---|---|---|---|---|---|---|---|---|---|---|
| cell culture vs non-immunized | 0.292616 | 0.831553 | 0.478317 | 0.15173 | 0.711181 | 0.433241 | **0.009468** | 0.868584 | 0.721146 | **2.15E-05** |
| cell culture versus immunized | 0.148155 | 0.439168 | 0.343384 | **0.044034** | 0.268772 | 0.066971 | 0.056373 | **0.020627** | 0.513456 | **2.32E-06** |
| non-immunized versus immunized | **0.024443** | 0.852597 | 0.128802 | 0.695185 | 0.661709 | 0.913779 | 0.628313 | 0.106978 | 0.445411 | **0.026975** |

| Position | 86 | 87 | 88 | 89 | 90 | 91 | 92 | 93 | 94 | 95 |
|---|---|---|---|---|---|---|---|---|---|---|
| cell culture vs non-immunized | 0.409373 | 0.986387 | 0.946881 | 0.980912 | 0.325207 | 0.586326 | 0.944317 | 0.384279 | 0.432374 | **0.037863** |
| cell culture versus immunized | 0.356005 | 0.570698 | 0.899164 | 0.378814 | 0.995405 | 0.628583 | 0.685288 | 0.890213 | 0.817771 | 0.053014 |
| non-immunized versus immunized | 0.870714 | 0.688724 | 0.861248 | 0.693908 | 0.339911 | 0.893354 | 0.819628 | 0.307083 | 0.397107 | 0.689807 |

| Position | 96 | 97 | 98 | 99 | 100 | 101 | 102 | 103 | 104 | 105 |
|---|---|---|---|---|---|---|---|---|---|---|
| cell culture vs non-immunized | **0.022901** | 0.301015 | 0.865686 | 0.887838 | 0.995476 | 0.65968 | 0.929282 | 0.979047 | 0.050824 | 0.656383 |
| cell culture versus immunized | **0.006642** | **0.011795** | 0.06641 | 0.259885 | 0.210621 | 0.200475 | 0.846323 | 0.456511 | 0.053506 | 0.528099 |
| non-immunized versus immunized | 0.43637 | **0.043317** | 0.05457 | 0.213527 | 0.281193 | 0.69395 | 0.946569 | 0.551922 | 0.441355 | 0.856807 |

| Position | 106 | 107 | 108 | 109 | 110 | 111 | 112 | 113 | 114 | 115 |
|---|---|---|---|---|---|---|---|---|---|---|
| cell culture vs non-immunized | 0.647505 | 0.082314 | 0.40209 | 0.063615 | 0.441874 | **0.033465** | 0.349483 | 0.234523 | 0.989344 | 0.731096 |
| cell culture versus immunized | 0.497467 | 0.866529 | **0.00466** | 0.084784 | 0.091535 | **0.021761** | 0.873197 | 0.357405 | 0.515814 | 0.72478 |
| non-immunized versus immunized | 0.799975 | 0.082814 | 0.577504 | 0.99272 | 0.638318 | 0.923318 | 0.369203 | 0.116553 | 0.678137 | 0.981135 |

| Position | 116 | 117 | 118 | 119 | 120 | 121 | 122 | 123 | 124 | 125 |
|---|---|---|---|---|---|---|---|---|---|---|
| cell culture vs non-immunized | 0.252417 | 0.665063 | 0.789354 | 0.945037 | 0.340332 | 0.10876 | 0.077768 | **0.019396** | 0.162794 | 0.082169 |
| cell culture versus immunized | 0.124056 | 0.595786 | 0.760622 | 0.760921 | 0.331341 | 0.806525 | 0.203812 | 0.330418 | 0.314308 | 0.381881 |
| non-immunized versus immunized | 0.394991 | 0.921051 | 0.997191 | 0.670657 | 0.742202 | 0.095301 | 0.230844 | **0.017402** | 0.524808 | 0.354578 |

| Position | 126 | 127 | 128 | 129 | 130 |
|---|---|---|---|---|---|
| cell culture vs non-immunized | 0.144042 | 0.434517 | 0.162201 | 0.727936 | 0.065676 |
| cell culture versus immunized | 0.970594 | 0.872394 | 0.884391 | 0.743456 | 0.212188 |
| non-immunized versus immunized | 0.229351 | 0.508503 | 0.15863 | 0.616044 | 0.273905 |

**Table S3:** Amino acid sequences found and the number of sequences within the individual samples.

| aa sequences | Sample ID (more details in Table S1) | | | | | | | | | | | | | | | | | |
|---|---|---|---|---|---|---|---|---|---|---|---|---|---|---|---|---|---|---|
| | 1 | 79 | 11 | 80 | 2 | 1.1 | 2.1 | 2.2 | 2.3 | 35 | 28 | 27 | 29 | 30 | 36 | 31 | 32 | 37 |
| RIENLNKKVNDGFLDIWTYNAELLVLLENERTLDYHDSNVKNLYEKVRSQLKNNA | 285 | 58 | 300 | 219 | 1279 | 1220 | 3030 | 2516 | 3284 | 355 | 26 | 20 | 81 | 117 | 389 | 6 | 53 | 63 |
| RIENLNKKVDDGFLDIWTYNVELLVLLENERTLDYHDSNVKNLYEKVRSQLKNNA | 34 | 10 | 46 | 27 | 161 | 175 | 371 | 335 | 475 | 40 | 9 | 9 | 14 | 29 | 32 | 7 | 18 | 6 |
| RIENLNKKVDDGFLDIWTYNAELLVLLENERTLDYHNSNVKNLYEKVRSQLKNNA | 13 | 1 | 29 | 11 | 54 | 34 | 93 | 71 | 92 | 19 | 2 | 1 | 12 | 9 | 18 | 3 | 5 | 5 |
| RIENLNKKVDDGFLDIWTYNAELLVLLENERTLDYHDSNVKNLYERVRSQLKNNA | 32 | 9 | 53 | 26 | 186 | 66 | 134 | 121 | 149 | 56 | 99 | 35 | 103 | 66 | 24 | 15 | 13 | 3 |
| RIENLNKKVDDGFLDIWTYNAELLVLLENERTLDYHDSNVKNLYEKVRSQLKNNA | 7467 | 4763 | 12934 | 8371 | 51611 | 16949 | 40950 | 33961 | 44916 | 10587 | 20523 | 9894 | 22254 | 30740 | 12232 | 3577 | 7628 | 1694 |
| RIENLNKKVDDGFLDIWTYNAELLVLLENERTLDYHDSNVKNLYEKVRNQLKNNA | 25 | 17 | 48 | 24 | 84 | 66 | 163 | 138 | 191 | 36 | 9 | 23 | 65 | 69 | 44 | 8 | 31 | 6 |
| RIENLNKKVDDGFLDIWTYNAELLVLLENEGTLDYHDSNVKNLYEKVRSQLKNNA | 2 | 2 | 4 | 4 | 10 | 4 | 13 | 5 | 11 | 1 | 5 | 5 | 9 | 10 | 2 | 1 | 4 | 1 |
| GIENLNKKVDDGFLDIWTYNAELLVLLENERTLDYHDSNVKNLYEKVRSQLKNNA | 6 | 1 | 18 | 13 | 122 | 34 | 71 | 73 | 83 | 35 | 60 | 13 | 30 | 43 | 15 | 5 | 12 | 5 |
| RIENLNKKVDDGFLDIWTYNAELLVLLENERTLDYHDSNVKNLYEKVRSQLKNSA | 3 | 1 | 4 | 4 | 11 | 10 | 28 | 15 | 20 | 6 | 0 | 1 | 3 | 6 | 5 | 1 | 1 | 1 |
| RIENLNKKVDDGFLDIWTYNAELLILLENERTLDYHDSNVKNLYEKVRSQLKNNA | 2 | 5 | 9 | 8 | 19 | 28 | 43 | 41 | 37 | 15 | 4 | 4 | 18 | 16 | 18 | 0 | 1 | 1 |
| RIENLNKRVDDGFLDIWTYNAELLVLLENERTLDYHDSNVKNLYEKVRSQLKNNA | 3 | 2 | 9 | 5 | 26 | 10 | 25 | 23 | 31 | 12 | 10 | 0 | 5 | 14 | 9 | 1 | 4 | 0 |
| RIENLNKKVDDGFLNIWTYNAELLVLLENERTLDYHDSNVKNLYEKVRSQLKNNA | 1 | 0 | 3 | 6 | 21 | 9 | 15 | 19 | 12 | 3 | 3 | 2 | 2 | 1 | 7 | 0 | 2 | 1 |
| RIENLNKKVDDGFLDVWTYNAELLVLLENERTLDYHDSNVKNLYEKVRSQLKNNA | 1 | 1 | 2 | 0 | 13 | 2 | 4 | 5 | 4 | 2 | 2 | 5 | 2 | 2 | 1 | 2 | 1 | 0 |
| RIENLNKKVDDGFLDIWTYNTELLVLLENERTLDYHDSNVKNLYEKVRSQLKNNA | 7 | 5 | 10 | 6 | 48 | 21 | 46 | 34 | 46 | 8 | 4 | 0 | 9 | 6 | 6 | 0 | 2 | 6 |
| RIENLNKKVDDGFLDIWTYNAELLVLLGNERTLDYHDSNVKNLYEKVRSQLKNNA | 1 | 1 | 1 | 2 | 12 | 1 | 1 | 4 | 11 | 4 | 6 | 5 | 2 | 6 | 3 | 0 | 1 | 0 |
| RIENLNKKVDDGFLDIWTYNAELLVLLENERTLGYHDSNVKNLYEKVRSQLKNNA | 3 | 2 | 2 | 5 | 15 | 5 | 17 | 14 | 22 | 4 | 6 | 4 | 7 | 8 | 3 | 0 | 0 | 1 |
| RIENLNKKVDDGFLDIWTYNAELLVLLENERTLDYHDSNVKNLYEKIRSQLKNNA | 7 | 2 | 8 | 7 | 62 | 6 | 32 | 28 | 30 | 12 | 3 | 0 | 60 | 10 | 7 | 0 | 2 | 1 |
| RIENLNKKVDDGFLDIWTYNAELLVLLENERTLDYHDSNVKNLYEEVRSQLKNNA | 10 | 4 | 5 | 8 | 25 | 13 | 36 | 32 | 39 | 9 | 1 | 0 | 1 | 6 | 14 | 0 | 7 | 2 |
| RIENLNKKVDDGFLDIWTYNAELLVLLENERTLDYHDSNVKNLCEKVRSQLKNNA | 7 | 2 | 3 | 6 | 25 | 9 | 11 | 10 | 21 | 6 | 3 | 3 | 4 | 10 | 2 | 0 | 5 | 0 |
| RIENLNKKVDDGFLDIWTYNAELLVLLENERTLDYHDLNVKNLYEKVRSQLKNNA | 4 | 1 | 3 | 2 | 7 | 9 | 17 | 14 | 20 | 4 | 2 | 0 | 4 | 13 | 3 | 1 | 0 | 1 |
| RIENLNKKVDDGFLDIWAYNAELLVLLENERTLDYHDSNVKNLYEKVRSQLKNNA | 1 | 0 | 3 | 2 | 18 | 4 | 12 | 8 | 11 | 1 | 3 | 3 | 4 | 4 | 2 | 0 | 2 | 3 |
| RIENLNKKVDGGFLDIWTYNAELLVLLENERTLDYHDSNVKNLYEKVRSQLKNNA | 1 | 2 | 1 | 1 | 6 | 3 | 2 | 4 | 4 | 1 | 2 | 2 | 2 | 1 | 0 | 0 | 1 | 0 |
| RIENLNKKVDDGFLDIWTYNAELLVLLENERTLDYHYSNVKNLYEKVRSQLKNNA | 3 | 2 | 1 | 2 | 5 | 7 | 23 | 33 | 33 | 6 | 0 | 0 | 1 | 2 | 1 | 2 | 4 | 0 |
| RIENLNKKVDDGFLDIWTYNAELLVLLENEKTLDYHDSNVKNLYEKVRSQLKNNA | 0 | 0 | 4 | 8 | 28 | 12 | 15 | 16 | 21 | 8 | 7 | 6 | 7 | 10 | 1 | 1 | 3 | 0 |
| RIEDLNKKVDDGFLDIWTYNAELLVLLENERTLDYHDSNVKNLYEKVRSQLKNNA | 7 | 0 | 2 | 1 | 8 | 10 | 29 | 19 | 38 | 5 | 2 | 0 | 1 | 7 | 2 | 2 | 2 | 0 |
| RIENLNKKVDDSFLDIWTYNAELLVLLENERTLDYHDSNVKNLYEKVRSQLKNNA | 1 | 0 | 4 | 3 | 5 | 7 | 15 | 13 | 16 | 7 | 1 | 0 | 3 | 6 | 2 | 0 | 1 | 0 |
| RIENLNKKVDDGSLDIWTYNAELLVLLENERTLDYHDSNVKNLYEKVRSQLKNNA | 1 | 1 | 3 | 2 | 13 | 3 | 7 | 5 | 9 | 2 | 3 | 0 | 4 | 3 | 2 | 0 | 0 | 0 |
| RIENLNKKVDDGFLDIWTYNAELLVLLENERTLDYHDSNVKNLYEKARSQLKNNA | 1 | 0 | 0 | 0 | 3 | 3 | 7 | 5 | 3 | 1 | 1 | 1 | 1 | 0 | 1 | 1 | 1 | 1 |
| RIENLNKKVDDGFLDIWTYNAELLVLLENERTLDYHDSNVENLYEKVRSQLKNNA | 7 | 0 | 8 | 0 | 24 | 11 | 26 | 21 | 29 | 5 | 4 | 25 | 8 | 8 | 2 | 0 | 4 | 0 |
| RIENLNKKVDDGFLDIWTYNAELLVLLENERTLDYHDSNMKNLYEKVRSQLKNNA | 0 | 2 | 5 | 1 | 4 | 2 | 2 | 2 | 4 | 2 | 1 | 3 | 0 | 7 | 1 | 0 | 2 | 0 |
| RIENLNKKVDDGFLDIWTYNAELLVLLEKERTLDYHDSNVKNLYEKVRSQLKNNA | 0 | 0 | 2 | 4 | 14 | 3 | 13 | 12 | 9 | 5 | 7 | 1 | 3 | 6 | 0 | 1 | 2 | 0 |
| RIKNLNKKVDDGFLDIWTYNAELLVLLENERTLDYHDSNVKNLYEKVRSQLKNNA | 0 | 0 | 3 | 2 | 7 | 2 | 4 | 3 | 10 | 2 | 1 | 0 | 0 | 4 | 4 | 0 | 2 | 2 |
| RIENLNKKVDDGFPDIWTYNAELLVLLENERTLDYHDSNVKNLYEKVRSQLKNNA | 0 | 1 | 2 | 0 | 1 | 1 | 2 | 1 | 3 | 2 | 2 | 1 | 2 | 7 | 0 | 0 | 0 | 2 |
| RIENLNKKVDDGFLGIWTYNAELLVLLENERTLDYHDSNVKNLYEKVRSQLKNNA | 1 | 0 | 3 | 0 | 12 | 5 | 19 | 12 | 16 | 7 | 4 | 6 | 7 | 2 | 3 | 0 | 0 | 0 |
| RIENLNKKVDDGFLDIWTYNAELLVLLENERTLDYHGSNVKNLYEKVRSQLKNNA | 1 | 0 | 3 | 3 | 4 | 6 | 13 | 11 | 12 | 0 | 2 | 1 | 0 | 2 | 6 | 0 | 0 | 1 |
| RIENLNKKVDDGFLDIWTYNAELLVLLENERTLDYHDSSVKNLYEKVRSQLKNNA | 0 | 1 | 1 | 1 | 3 | 2 | 2 | 6 | 4 | 0 | 0 | 1 | 4 | 3 | 3 | 0 | 1 | 0 |
| RIENLNKKVDDGFLDIWTYNAELLVLLENERTLDYHDSNVRNLYEKVRSQLKNNA | 0 | 0 | 4 | 0 | 6 | 1 | 4 | 3 | 6 | 2 | 2 | 6 | 1 | 1 | 1 | 0 | 0 | 1 |
| RIENLNKKVDDGFLDIWTYNAELLVLLENERTLDYHDSNVKNLYGKVRSQLKNNA | 4 | 0 | 0 | 2 | 9 | 5 | 7 | 2 | 7 | 0 | 2 | 0 | 1 | 1 | 1 | 1 | 3 | 0 |
| RIENLNKKVDDGFLDIWTYNAELLVLLENERTLDYHDSNVKNLYEKVRSQLRNNA | 1 | 0 | 2 | 1 | 1 | 0 | 2 | 2 | 2 | 0 | 1 | 0 | 2 | 3 | 2 | 1 | 0 | 1 |
| RIENLNKKVDDGFLDIWTYNAELLVLLENERTLDYHDSNVKNLYEKVGSQLKNNA | 1 | 0 | 1 | 1 | 6 | 2 | 1 | 2 | 2 | 0 | 1 | 1 | 1 | 0 | 2 | 3 | 0 | 0 |
| RIENLNKKVDDGFLDIWTYNAELLVLLENERTLDYHDSNVKDLYEKVRSQLKNNA | 1 | 0 | 5 | 1 | 6 | 3 | 10 | 5 | 8 | 3 | 2 | 2 | 0 | 4 | 2 | 0 | 0 | 0 |
| RIENLNKKVDDGFLDIWTYNAELLVLLENERTLDYHDSKVKNLYEKVRSQLKNNA | 1 | 0 | 1 | 2 | 9 | 2 | 5 | 7 | 14 | 2 | 1 | 1 | 1 | 0 | 1 | 0 | 0 | 0 |
| RIENLNKKVDDGFLDIWTYNAELLVLLENERALDYHDSNVKNLYEKVRSQLKNNA | 0 | 1 | 3 | 0 | 12 | 1 | 8 | 6 | 8 | 3 | 5 | 2 | 1 | 5 | 0 | 0 | 1 | 0 |
| RIGNLNKKVDDGFLDIWTYNAELLVLLENERTLDYHDSNVKNLYEKVRSQLKNNA | 1 | 0 | 2 | 0 | 9 | 6 | 5 | 8 | 6 | 2 | 2 | 0 | 0 | 2 | 1 | 0 | 1 | 0 |
| RIESLNKKVDDGFLDIWTYNAELLVLLENERTLDYHDSNVKNLYEKVRSQLKNNA | 0 | 0 | 0 | 2 | 12 | 1 | 3 | 1 | 5 | 3 | 3 | 0 | 1 | 2 | 0 | 1 | 0 | 1 |
| RIENLNKKVGDGFLDIWTYNAELLVLLENERTLDYHDSNVKNLYEKVRSQLKNNA | 1 | 0 | 2 | 0 | 3 | 4 | 4 | 2 | 2 | 3 | 0 | 2 | 1 | 1 | 0 | 1 | 0 | 0 |
| RIENLNKKVDNGFLDIWTYNAELLVLLENERTLDYHDSNVKNLYEKVRSQLKNNA | 1 | 4 | 1 | 2 | 4 | 2 | 3 | 3 | 5 | 0 | 0 | 0 | 4 | 3 | 2 | 0 | 0 | 0 |
| RIENLNKKVDDGFLDIWTYNAELLVLLKNERTLDYHDSNVKNLYEKVRSQLKNNA | 0 | 2 | 2 | 5 | 4 | 2 | 4 | 4 | 6 | 2 | 0 | 0 | 2 | 0 | 0 | 0 | 4 | 1 |
| RIENLNKKVDDGFLDIWTYNAELLVLLENERTLDYHDSNVKSLYEKVRSQLKNNA | 0 | 0 | 1 | 0 | 8 | 6 | 13 | 10 | 10 | 4 | 3 | 1 | 0 | 4 | 0 | 1 | 1 | 0 |
| RIENLNKKVDDGFLDIWTYNAELLVLLENERTLDYHDSNVKNLYEKVSSQLKNNA | 0 | 0 | 3 | 1 | 2 | 1 | 2 | 1 | 4 | 1 | 1 | 1 | 1 | 1 | 0 | 0 | 0 | 0 |
| RIENLNKKVDDGFLDIWTYNAELLVLLENERTLDHHDSNVKNLYEKVRSQLKNNA | 0 | 0 | 3 | 2 | 6 | 9 | 10 | 10 | 17 | 0 | 1 | 0 | 1 | 2 | 2 | 0 | 2 | 0 |
| RIENLNKKVDDGFLDIWTYNAELLVLLENERNLDYHDSNVKNLYEKVRSQLKNNA | 0 | 1 | 1 | 1 | 3 | 1 | 4 | 4 | 3 | 1 | 1 | 3 | 1 | 0 | 0 | 0 | 0 | 0 |
| RIENLNKKVDDGFLDIWTYNAELLVILENERTLDYHDSNVKNLYEKVRSQLKNNA | 1 | 0 | 8 | 1 | 1 | 2 | 3 | 6 | 3 | 0 | 1 | 0 | 1 | 1 | 0 | 0 | 1 | 0 |
| RVENLNKKVDDGFLDIWTYNAELLVLLENERTLDYHDSNVKNLYEKVRSQLKNNA | 3 | 0 | 1 | 0 | 9 | 0 | 6 | 7 | 2 | 0 | 1 | 1 | 2 | 2 | 0 | 1 | 0 | 0 |
| RIENLNKKVDDGFLDIWTYNAKLLVLLENERTLDYHDSNVKNLYEKVRSQLKNNA | 0 | 0 | 3 | 2 | 3 | 4 | 9 | 5 | 5 | 2 | 1 | 0 | 3 | 0 | 3 | 0 | 0 | 0 |
| RIENLNKKVDDGFLDIWTYNAELLVLSENERTLDYHDSNVKNLYEKVRSQLKNNA | 1 | 0 | 2 | 0 | 3 | 0 | 4 | 3 | 2 | 0 | 2 | 1 | 3 | 0 | 1 | 0 | 1 | 0 |
| RIENLNKKVDDGFLDIWTYNAELLVLLENERTLDYHDSNVKNLYEQVRSQLKNNA | 1 | 1 | 2 | 0 | 1 | 1 | 1 | 1 | 0 | 0 | 0 | 0 | 1 | 3 | 1 | 0 | 1 | 0 |
| RIENLNKKVDDGFLDIWTYNAELLVLLENERTLDYHDSNVKNLYEKVRSQLKNNV | 0 | 0 | 2 | 0 | 8 | 2 | 4 | 4 | 4 | 3 | 1 | 1 | 0 | 6 | 0 | 0 | 4 | 0 |
| RIENLNKKVDDGFLDIWTYNAELLVLLENERTLDYHDSNVKNLYEKVRSQLKNDA | 0 | 1 | 0 | 0 | 2 | 0 | 2 | 1 | 4 | 0 | 3 | 0 | 4 | 2 | 1 | 1 | 1 | 0 |
| RIENLNKKVDDGFLDIWTYNAELLVLLENERTLDYHDSNVKNLYDKVRSQLKNNA | 0 | 0 | 1 | 0 | 1 | 2 | 1 | 3 | 3 | 0 | 1 | 1 | 2 | 0 | 2 | 0 | 1 | 0 |
| RIENLNKKVDDGFLDIWTYNAELLVLLENERTFDYHDSNVKNLYEKVRSQLKNNA | 1 | 0 | 2 | 1 | 1 | 1 | 3 | 2 | 2 | 0 | 1 | 0 | 2 | 2 | 0 | 0 | 0 | 0 |
| RIENLNKKVDDGFLDIWIYNAELLVLLENERTLDYHDSNVKNLYEKVRSQLKNNA | 0 | 2 | 0 | 0 | 2 | 2 | 1 | 2 | 3 | 1 | 1 | 1 | 2 | 1 | 0 | 0 | 0 | 0 |
| RIENLNKKVDDGFLDIRTYNAELLVLLENERTLDYHDSNVKNLYEKVRSQLKNNA | 1 | 0 | 1 | 1 | 4 | 2 | 2 | 3 | 1 | 3 | 2 | 0 | 1 | 0 | 0 | 0 | 0 | 0 |
| RIENLNKEVDDGFLDIWTYNAELLVLLENERTLDYHDSNVKNLYEKVRSQLKNNA | 0 | 0 | 2 | 0 | 6 | 1 | 2 | 6 | 6 | 0 | 1 | 0 | 2 | 3 | 1 | 0 | 0 | 1 |
| GIENLNKKVNDGFLDIWTYNAELLVLLENERTLDYHDSNVKNLYEKVRSQLKNNA | 1 | 0 | 1 | 1 | 3 | 2 | 7 | 4 | 3 | 2 | 0 | 0 | 0 | 0 | 1 | 0 | 1 | 0 |
| RIEYLNKKVDDGFLDIWTYNAELLVLLENERTLDYHDSNVKNLYEKVRSQLKNNA | 1 | 0 | 1 | 0 | 6 | 4 | 11 | 4 | 6 | 0 | 0 | 0 | 1 | 1 | 0 | 0 | 0 | 1 |
| RIENLNKKVDDGFLDIWTYNAELLVPLENERTLDYHDSNVKNLYEKVRSQLKNNA | 1 | 0 | 0 | 3 | 4 | 0 | 2 | 2 | 3 | 2 | 2 | 0 | 0 | 2 | 0 | 1 | 0 | 0 |
| RIENLNKKVDDGFLDIWTYNAELLVLLENGRTLDYHDSNVKNLYEKVRSQLKNNA | 1 | 0 | 0 | 0 | 0 | 0 | 5 | 4 | 6 | 2 | 3 | 1 | 0 | 1 | 1 | 0 | 1 | 0 |
| RIENLNKKVDDGFLDIWTYNAELLVLLENERTLDYYDSNVKNLYEKVRSQLKNNA | 1 | 0 | 2 | 0 | 2 | 3 | 9 | 9 | 15 | 0 | 0 | 0 | 2 | 2 | 0 | 0 | 0 | 1 |
| RIENLNKKVDDGFLDIWTYNAELLVLLENERTLDYRDSNVKNLYEKVRSQLKNNA | 0 | 1 | 2 | 0 | 2 | 1 | 1 | 7 | 3 | 1 | 2 | 0 | 0 | 0 | 0 | 0 | 1 | 0 |
| RIENLNKKVDDGFLDIWTYNAELLVLLENERTLDYHDSNVKNLYEKVRSQSKNNA | 1 | 0 | 0 | 3 | 1 | 1 | 3 | 3 | 3 | 0 | 2 | 0 | 1 | 1 | 0 | 0 | 0 | 0 |
| RIENLNKKVDDGFLDIWTYNAELLVLLENERTLDYHDSNVKNLYEKVRSQLKDNA | 0 | 0 | 4 | 0 | 4 | 2 | 9 | 4 | 6 | 1 | 0 | 2 | 3 | 0 | 0 | 0 | 2 | 0 |
| RIENLNKKVDDGFLDIWTYNAELLVLLENERTLDYHDSNVKNLYEKVRRQLKNNA | 0 | 0 | 0 | 1 | 3 | 1 | 1 | 1 | 6 | 1 | 0 | 0 | 1 | 19 | 0 | 1 | 0 | 0 |
| RIENLNKKVDDGFLDIWTYNAELLVLLENERTLDNHDSNVKNLYEKVRSQLKNNA | 0 | 0 | 1 | 2 | 4 | 8 | 11 | 4 | 7 | 2 | 0 | 0 | 1 | 0 | 1 | 0 | 0 | 0 |
| RIENLNKKVDDGFLDILTYNAELLVLLENERTLDYHDSNVKNLYEKVRSQLKNNA | 2 | 1 | 0 | 0 | 1 | 1 | 3 | 3 | 2 | 3 | 0 | 1 | 0 | 2 | 0 | 0 | 0 | 0 |
| RIENLNEKVDDGFLDIWTYNAELLVLLENERTLDYHDSNVKNLYEKVRSQLKNNA | 2 | 0 | 0 | 0 | 3 | 6 | 6 | 4 | 7 | 3 | 2 | 0 | 2 | 0 | 0 | 0 | 1 | 0 |
| RIENLNRKVDDGFLDIWTYNAELLVLLENERTLDYHDSNVKNLYEKVRSQLKNNA | 0 | 0 | 1 | 1 | 0 | 1 | 8 | 4 | 4 | 0 | 0 | 0 | 2 | 0 | 5 | 2 | 0 | 0 |
| RIENLNKKVNDGFLDIWTYNAELLVLLENERTLDYHDSNVKNLYERVRSQLKNNA | 1 | 0 | 0 | 0 | 3 | 3 | 7 | 8 | 10 | 1 | 0 | 0 | 0 | 0 | 1 | 0 | 0 | 1 |
| RIENLNKKVEDGFLDIWTYNAELLVLLENERTLDYHDSNVKNLYEKVRSQLKNNA | 0 | 0 | 0 | 0 | 1 | 1 | 2 | 6 | 1 | 2 | 0 | 0 | 0 | 0 | 3 | 1 | 1 | 0 |
| RIENLNKKVDDGLLDIWTYNAELLVLLENERTLDYHDSNVKNLYEKVRSQLKNNA | 0 | 0 | 1 | 2 | 5 | 0 | 7 | 3 | 3 | 0 | 0 | 0 | 1 | 1 | 3 | 0 | 0 | 0 |
| RIENLNKKVDDGFLDTWTYNAELLVLLENERTLDYHDSNVKNLYEKVRSQLKNNA | 2 | 0 | 0 | 0 | 5 | 0 | 1 | 2 | 1 | 0 | 1 | 0 | 1 | 1 | 0 | 0 | 1 | 0 |
| RIENLNKKVDDGFLDIWTYNAELLVLLENKRTLDYHDSNVKNLYEKVRSQLKNNA | 0 | 0 | 1 | 0 | 3 | 0 | 1 | 0 | 1 | 1 | 1 | 0 | 1 | 1 | 1 | 0 | 0 | 0 |
| RIENLNKKVDDGFLDIWTYNAELLVLLENERTWDYHDSNVKNLYEKVRSQLKNNA | 1 | 0 | 2 | 0 | 5 | 3 | 4 | 2 | 5 | 2 | 0 | 0 | 1 | 0 | 0 | 0 | 0 | 0 |
| RIENLNKKVDDGFLDIWTYNAELLVLLENERTLDYHDSTVKNLYEKVRSQLKNNA | 1 | 0 | 2 | 0 | 0 | 0 | 1 | 4 | 1 | 1 | 0 | 1 | 0 | 2 | 1 | 0 | 0 | 0 |
| RIENLNKKVDDGFLDIWTYNAELLVLLENERTLDYHDSNVKNLYEKVRSRLKNNA | 0 | 0 | 1 | 5 | 1 | 0 | 4 | 3 | 0 | 0 | 2 | 0 | 0 | 3 | 3 | 0 | 1 | 0 |
| RIENLNKKVDDGFLDIWTYNAELLVLLENERTLDYHDSNVKNLYEKVRSQIKNNA | 0 | 0 | 2 | 0 | 2 | 1 | 1 | 1 | 2 | 2 | 2 | 0 | 0 | 1 | 0 | 0 | 0 | 0 |
| RIENLNKKVDDGFLDIWTYNAELLVLLENERTLDYHDSNVKNLYEKVRGQLKNNA | 2 | 0 | 0 | 0 | 7 | 2 | 5 | 3 | 2 | 2 | 1 | 0 | 11 | 0 | 0 | 0 | 0 | 0 |
| RIENLNKKVDDGFLDIWTYNAELLVLLENERTLDYHDSNVKNLHEKVRSQLKNNA | 0 | 0 | 0 | 0 | 1 | 2 | 3 | 3 | 1 | 0 | 1 | 0 | 1 | 1 | 0 | 0 | 2 | 0 |
| RIENLNKKVDDGFLDIWTYNAELLVLLENERTLDYHDSNVKKLYEKVRSQLKNNA | 1 | 2 | 1 | 0 | 3 | 0 | 2 | 1 | 3 | 0 | 0 | 0 | 1 | 0 | 0 | 0 | 1 | 0 |
| RIENLNKKVDDGFLDIWTYNAELLVLLENERTLDYHDSNAKNLYEKVRSQLKNNA | 1 | 0 | 1 | 0 | 2 | 0 | 0 | 0 | 4 | 1 | 0 | 0 | 2 | 1 | 0 | 0 | 1 | 1 |
| RIENLNKKVDDGFLDIWTYNAELLVLLENERTLDYHDPNVKNLYEKVRSQLKNNA | 0 | 0 | 0 | 0 | 1 | 0 | 2 | 1 | 1 | 1 | 0 | 1 | 1 | 2 | 2 | 0 | 0 | 0 |
| RIENLNKKVDDGFLDIWTYNAELLVLLENERTLDCHDSNVKNLYEKVRSQLKNNA | 0 | 1 | 0 | 1 | 0 | 2 | 3 | 3 | 0 | 1 | 1 | 0 | 3 | 1 | 0 | 0 | 0 | 0 |
| RIENLNKKVDDGFLDIWTYHAELLVLLENERTLDYHDSNVKNLYEKVRSQLKNNA | 0 | 0 | 1 | 0 | 4 | 0 | 5 | 1 | 4 | 3 | 4 | 1 | 0 | 0 | 0 | 0 | 1 | 0 |
| RIENLNKKVDDGFLDIWTCNAELLVLLENERTLDYHDSNVKNLYEKVRSQLKNNA | 0 | 0 | 1 | 0 | 2 | 6 | 4 | 3 | 2 | 1 | 2 | 0 | 4 | 0 | 0 | 0 | 0 | 0 |
| RIENLNKKVDDDFLDIWTYNAELLVLLENERTLDYHDSNVKNLYEKVRSQLKNNA | 0 | 2 | 3 | 0 | 3 | 1 | 5 | 0 | 4 | 0 | 0 | 1 | 0 | 1 | 0 | 0 | 0 | 1 |
| RIENLDKKVDDGFLDIWTYNAELLVLLENERTLDYHDSNVKNLYEKVRSQLKNNA | 0 | 1 | 0 | 0 | 4 | 0 | 4 | 5 | 1 | 1 | 1 | 0 | 0 | 1 | 0 | 1 | 0 | 0 |
| RIENFNKKVDDGFLDIWTYNAELLVLLENERTLDYHDSNVKNLYEKVRSQLKNNA | 0 | 0 | 3 | 0 | 3 | 0 | 3 | 1 | 5 | 1 | 3 | 0 | 1 | 3 | 0 | 0 | 0 | 0 |
| RIEKLNKKVDDGFLDIWTYNAELLVLLENERTLDYHDSNVKNLYEKVRSQLKNNA | 0 | 0 | 0 | 1 | 4 | 2 | 2 | 1 | 5 | 1 | 0 | 0 | 0 | 2 | 0 | 0 | 1 | 0 |
| RIENLTKKVDDGFLDIWTYNAELLVLLENERTLDYHDSNVKNLYEKVRSQLKNNA | 0 | 0 | 1 | 1 | 3 | 0 | 0 | 1 | 1 | 1 | 1 | 0 | 0 | 2 | 0 | 0 | 0 | 0 |
| RIENLNKKVDDGFLDIWTYNAELLVLLENERTLNYHDSNVKNLYEKVRSQLKNNA | 0 | 2 | 0 | 2 | 4 | 1 | 0 | 7 | 0 | 1 | 0 | 0 | 1 | 2 | 0 | 0 | 0 | 0 |
| RIENLNKKVDDGFLDIWTYNAELLVLLENERTLDYHDSNVKNSYEKVRSQLKNNA | 0 | 0 | 0 | 0 | 5 | 0 | 1 | 4 | 4 | 1 | 1 | 3 | 0 | 1 | 0 | 0 | 0 | 0 |
| RIENLNKKVDDGFLDIWTYNAELLVLLENERTLDYHDSNVKNLYEKVRSQLKNNT | 0 | 0 | 4 | 1 | 0 | 1 | 3 | 5 | 11 | 3 | 0 | 0 | 0 | 1 | 0 | 0 | 0 | 0 |
| RIENLNKKVDDGFLDIWTYNAELLVLLENERTLDYHDSNVKNLYEKVRSPLKNNA | 0 | 0 | 0 | 1 | 1 | 2 | 3 | 2 | 2 | 0 | 0 | 1 | 1 | 0 | 0 | 0 | 0 | 0 |
| RIENLNKKVDDGFLDIWTYNAELLVLLENERTLDYHDSNVKNLYEKVRSHLKNNA | 0 | 0 | 2 | 0 | 2 | 0 | 0 | 1 | 1 | 1 | 1 | 0 | 2 | 2 | 0 | 0 | 0 | 0 |
| RIENLNKKVDDGFLDIWTYNAELLVLLENERTLDYHDSDVKNLYEKVRSQLKNNA | 0 | 0 | 0 | 0 | 2 | 0 | 2 | 1 | 4 | 1 | 0 | 0 | 1 | 2 | 1 | 0 | 0 | 0 |
| RIENLNKKVDDGFLDIWTYNAELLVLLENERILDYHDSNVKNLYEKVRSQLKNNA | 0 | 0 | 0 | 0 | 4 | 2 | 3 | 2 | 1 | 0 | 0 | 1 | 1 | 1 | 0 | 0 | 0 | 0 |
| RIENLNKKVDDGFLDIWTYDAELLVLLENERTLDYHDSNVKNLYEKVRSQLKNNA | 0 | 0 | 0 | 1 | 4 | 1 | 1 | 1 | 5 | 0 | 0 | 1 | 0 | 0 | 2 | 0 | 0 | 0 |
| RIENLNKKVDDGFLDIWTHNAELLVLLENERTLDYHDSNVKNLYEKVRSQLKNNA | 0 | 0 | 0 | 0 | 4 | 2 | 0 | 4 | 8 | 2 | 3 | 2 | 4 | 0 | 0 | 0 | 0 | 0 |
| RIENLNKKADDGFLDIWTYNAELLVLLENERTLDYHDSNVKNLYEKVRSQLKNNA | 0 | 0 | 0 | 0 | 1 | 1 | 1 | 1 | 1 | 1 | 0 | 1 | 0 | 1 | 0 | 0 | 0 | 0 |
| RMENLNKKVDDGFLDIWTYNAELLVLLENERTLDYHDSNVKNLYEKVRSQLKNNA | 0 | 0 | 0 | 1 | 5 | 0 | 2 | 2 | 5 | 3 | 0 | 1 | 0 | 0 | 0 | 0 | 0 | 0 |
| RIETLNKKVDDGFLDIWTYNAELLVLLENERTLDYHDSNVKNLYEKVRSQLKNNA | 0 | 0 | 2 | 0 | 9 | 1 | 4 | 1 | 2 | 0 | 0 | 0 | 1 | 0 | 0 | 0 | 0 | 0 |
| RIENLSKKVDDGFLDIWTYNAELLVLLENERTLDYHDSNVKNLYEKVRSQLKNNA | 0 | 0 | 0 | 0 | 2 | 1 | 2 | 3 | 2 | 1 | 0 | 0 | 0 | 2 | 0 | 0 | 0 | 0 |
| RIENLNKKVDDVFLDIWTYNAELLVLLENERTLDYHDSNVKNLYEKVRSQLKNNA | 0 | 0 | 2 | 1 | 0 | 0 | 0 | 0 | 1 | 0 | 1 | 2 | 1 | 0 | 1 | 0 | 0 | 0 |
| RIENLNKKVDDGFLEIWTYNAELLVLLENERTLDYHDSNVKNLYEKVRSQLKNNA | 0 | 1 | 0 | 2 | 0 | 1 | 2 | 2 | 0 | 0 | 0 | 0 | 1 | 0 | 0 | 0 | 0 | 1 |

| | | | | | | | | | | | | | | | | | | |
|---|---|---|---|---|---|---|---|---|---|---|---|---|---|---|---|---|---|---|
| RIENLNKKVDDGFLDIWTYNAEPLVLLENERTLDYHDSNVKNLYEKVRSQLKNNA | 0 | 0 | 0 | 0 | 2 | 1 | 1 | 5 | 6 | 0 | 0 | 0 | 0 | 1 | 0 | 0 | 1 | 0 |
| RIENLNKKVDDGFLDIWTYNAELLVLLENERTLDYPDSNVKNLYEKVRSQLKNNA | 0 | 0 | 1 | 0 | 3 | 0 | 1 | 2 | 1 | 0 | 0 | 0 | 0 | 4 | 0 | 1 | 0 | 0 |
| RIENLNKKVDDGFLDIWTYNAELLVLLENERTLDYHESNVKNLYEKVRSQLKNNA | 0 | 0 | 1 | 0 | 1 | 0 | 1 | 2 | 1 | 0 | 0 | 0 | 1 | 0 | 0 | 0 | 1 | 0 |
| RIENLNKKVDDGFLDIWTYNAELLVLLENERTLDYHDSNVKNLYEKVRSQLQNNA | 0 | 0 | 1 | 0 | 1 | 1 | 2 | 0 | 2 | 1 | 0 | 0 | 0 | 1 | 0 | 0 | 0 | 0 |
| RIENLNKKVDDGFLDIWTYNAELLVLLENERTLDYHDSNVKNLYEKVRSQLNNNA | 0 | 0 | 1 | 0 | 0 | 0 | 1 | 1 | 1 | 0 | 0 | 0 | 1 | 1 | 0 | 0 | 1 | 0 |
| RIENLNKKVDDGFLDIWTYNAELLVLLENERTLDYHDSNVKNLYEKVRSQLENNA | 0 | 1 | 0 | 1 | 1 | 1 | 3 | 0 | 3 | 0 | 0 | 0 | 0 | 0 | 0 | 0 | 1 | 0 |
| RIENLNKKVDDGFLDIWTYNAELLVLLENERTLDYHDSNVKNLYEKVKNQLKNNA | 0 | 0 | 0 | 1 | 0 | 2 | 2 | 0 | 4 | 0 | 0 | 0 | 8 | 0 | 0 | 1 | 1 | 0 |
| RIENLNKKVDDGFLDIWTYNAELLVLLENERTLDYHDSNVKNLYEKVISQLKNNA | 0 | 1 | 1 | 0 | 0 | 0 | 4 | 1 | 2 | 0 | 0 | 0 | 0 | 0 | 0 | 1 | 1 | 0 |
| RIENLNKKVDDGFLDIWTYNAELLVLLEHERTLDYHDSNVKNLYEKVRSQLKNNA | 1 | 0 | 0 | 0 | 1 | 0 | 2 | 1 | 1 | 1 | 0 | 0 | 0 | 1 | 0 | 0 | 0 | 0 |
| RIENLNKKVDDGFLDIWTYNAELLALLENERTLDYHDSNVKNLYEKVRSQLKNNA | 0 | 0 | 0 | 1 | 0 | 0 | 0 | 1 | 1 | 1 | 0 | 2 | 1 | 1 | 0 | 0 | 0 | 0 |
| RTENLNKKVDDGFLDIWTYNAELLVLLENERTLDYHDSNVKNLYEKVRSQLKNNA | 0 | 0 | 0 | 0 | 2 | 0 | 6 | 2 | 0 | 0 | 3 | 0 | 0 | 1 | 1 | 0 | 0 | 0 |
| RRENLNKKVDDGFLDIWTYNAELLVLLENERTLDYHDSNVKNLYEKVRSQLKNNA | 0 | 0 | 0 | 1 | 2 | 0 | 3 | 0 | 2 | 1 | 0 | 0 | 1 | 0 | 0 | 0 | 0 | 0 |
| RLENLNKKVDDGFLDIWTYNAELLVLLENERTLDYHDSNVKNLYEKVRSQLKNNA | 0 | 0 | 0 | 0 | 1 | 1 | 1 | 3 | 0 | 0 | 0 | 0 | 0 | 1 | 0 | 0 | 1 | 0 |
| RIENSNKKVDDGFLDIWTYNAELLVLLENERTLDYHDSNVKNLYEKVRSQLKNNA | 1 | 0 | 0 | 0 | 2 | 1 | 2 | 2 | 0 | 0 | 0 | 0 | 1 | 0 | 0 | 0 | 0 | 0 |
| RIENLNNKVDDGFLDIWTYNAELLVLLENERTLDYHDSNVKNLYEKVRSQLKNNA | 0 | 0 | 1 | 0 | 0 | 1 | 1 | 1 | 1 | 0 | 0 | 0 | 1 | 0 | 0 | 0 | 0 | 0 |
| RIENLNKKVNDGFLDIWTYNAELLVLLENERTLDYHDSNVKNLYEKVRNQLKNNA | 0 | 0 | 0 | 0 | 1 | 1 | 3 | 1 | 5 | 0 | 0 | 0 | 0 | 0 | 0 | 0 | 1 | 0 |
| RIENLNKKVDDGFMDIWTYNAELLVLLENERTLDYHDSNVKNLYEKVRSQLKNNA | 0 | 0 | 2 | 0 | 1 | 0 | 1 | 0 | 1 | 0 | 0 | 0 | 0 | 0 | 2 | 0 | 0 | 1 |
| RIENLNKKVDDGFLDIWTYNAGLLVLLENERTLDYHDSNVKNLYEKVRSQLKNNA | 0 | 0 | 1 | 0 | 0 | 1 | 1 | 2 | 2 | 0 | 0 | 0 | 1 | 0 | 0 | 0 | 0 | 0 |
| RIENLNKKVDDGFLDIWTYNAEMLVLLENERTLDYHDSNVKNLYEKVRSQLKNNA | 1 | 0 | 5 | 0 | 0 | 0 | 2 | 0 | 1 | 1 | 0 | 0 | 0 | 2 | 0 | 0 | 0 | 0 |
| RIENLNKKVDDGFLDIWTYNAELSVLLENERTLDYHDSNVKNLYEKVRSQLKNNA | 0 | 0 | 0 | 0 | 2 | 2 | 1 | 0 | 0 | 1 | 0 | 0 | 1 | 1 | 0 | 0 | 0 | 0 |
| RIENLNKKVDDGFLDIWTYNAELLVLLESERTLDYHDSNVKNLYEKVRSQLKNNA | 2 | 0 | 0 | 0 | 0 | 2 | 4 | 0 | 4 | 0 | 0 | 0 | 0 | 2 | 0 | 0 | 1 | 0 |
| RIENLNKKVDDGFLDIWTYNAELLVLLENERTSDYHDSNVKNLYEKVRSQLKNNA | 1 | 0 | 0 | 0 | 0 | 0 | 6 | 2 | 1 | 1 | 0 | 0 | 0 | 1 | 0 | 0 | 0 | 0 |
| RIENLNKKVDDGFLDIWTYNAELLVLLENERTLEYHDSNVKNLYEKVRSQLKNNA | 0 | 1 | 0 | 0 | 1 | 1 | 2 | 3 | 1 | 0 | 0 | 0 | 0 | 0 | 0 | 0 | 0 | 0 |
| RIENLNKKVDDGFLDIWTYNAELLVLLENERTLDYNDSNVKNLYEKVRSQLKNNA | 1 | 0 | 2 | 0 | 0 | 0 | 3 | 0 | 3 | 1 | 0 | 0 | 0 | 1 | 0 | 0 | 0 | 0 |
| RIENLNKKVDDGFLDIWTYNAELLVLLENERTLDYHDSNVKNLYEKVRSQLTNNA | 0 | 0 | 0 | 2 | 3 | 1 | 2 | 0 | 1 | 0 | 0 | 0 | 0 | 10 | 0 | 0 | 0 | 0 |
| RIENLNKKVDDGFLDIWTYNAELLVLLENERTLDYHDSNVKNLYEKVRSQLKNKA | 0 | 0 | 1 | 0 | 0 | 1 | 0 | 1 | 2 | 0 | 1 | 0 | 0 | 1 | 0 | 0 | 0 | 0 |
| RIENLNKKVDDGFLDIWTYNAELLVLLENERTLDYHDSNVKNLYEKVRSQLKHNA | 0 | 0 | 0 | 0 | 2 | 0 | 2 | 2 | 2 | 0 | 0 | 2 | 1 | 0 | 0 | 0 | 0 | 0 |
| RIENLNKKVDDGFLDIWTYNAELLVLLENERTLDYHDSNVKNLYEGVRSQLKNNA | 0 | 1 | 0 | 0 | 2 | 1 | 1 | 0 | 0 | 0 | 1 | 0 | 1 | 0 | 0 | 0 | 0 | 0 |
| RIENLNKKVDDGFLDIWTYNAELLVLLENERTLDYHDSNVKNLSEKVRSQLKNNA | 0 | 0 | 0 | 0 | 1 | 0 | 2 | 1 | 1 | 0 | 0 | 0 | 0 | 0 | 1 | 0 | 1 | 0 |
| RIENLNKKVDDGFLDIWTYNAELLVLLEDERTLDYHDSNVKNLYEKVRSQLKNNA | 0 | 0 | 1 | 0 | 5 | 0 | 4 | 0 | 1 | 0 | 0 | 1 | 0 | 3 | 0 | 0 | 0 | 0 |
| RIENLNKKVDDGFLDIWTYNADLLVLLENERTLDYHDSNVKNLYEKVRSQLKNNA | 1 | 0 | 0 | 0 | 0 | 3 | 0 | 1 | 3 | 1 | 0 | 0 | 0 | 2 | 0 | 0 | 0 | 0 |
| RIENLNKKVDDGFLDIWTYKAELLVLLENERTLDYHDSNVKNLYEKVRSQLKNNA | 0 | 0 | 1 | 0 | 0 | 1 | 4 | 0 | 1 | 0 | 1 | 0 | 0 | 0 | 1 | 0 | 0 | 0 |
| RKENLNKKVDDGFLDIWTYNAELLVLLENERTLDYHDSNVKNLYEKVRSQLKNNA | 0 | 0 | 0 | 0 | 2 | 1 | 0 | 2 | 0 | 1 | 0 | 0 | 0 | 2 | 0 | 0 | 0 | 0 |
| RIENLNTKVDDGFLDIWTYNAELLVLLENERTLDYHDSNVKNLYEKVRSQLKNNA | 0 | 0 | 1 | 0 | 3 | 0 | 1 | 2 | 0 | 1 | 0 | 0 | 0 | 0 | 0 | 0 | 0 | 0 |
| RIENLNKTVDDGFLDIWTYNAELLVLLENERTLDYHDSNVKNLYEKVRSQLKNNA | 0 | 0 | 0 | 0 | 3 | 0 | 2 | 3 | 0 | 0 | 0 | 0 | 0 | 1 | 0 | 0 | 0 | 1 |
| RIENLNKKVNDGFLDIWTYNAELLILLENERTLDYHDSNVKNLYEKVRSQLKNNA | 0 | 0 | 0 | 0 | 1 | 1 | 1 | 1 | 1 | 0 | 0 | 0 | 0 | 0 | 0 | 0 | 0 | 0 |
| RIENLNKKVDDGYLDIWTYNAELLVLLENERTLDYHDSNVKNLYEKVRSQLKNNA | 0 | 0 | 1 | 0 | 0 | 0 | 1 | 1 | 0 | 1 | 0 | 0 | 0 | 1 | 0 | 0 | 0 | 0 |
| RIENLNKKVDDGFLDIWTYNAELMVLLENERTLDYHDSNVKNLYEKVRSQLKNNA | 0 | 0 | 0 | 1 | 0 | 0 | 2 | 1 | 0 | 1 | 0 | 0 | 0 | 2 | 0 | 0 | 0 | 0 |
| RIENLNKKVDDGFLDIWTYNAELLVLLENERTLDYQDSNVKNLYEKVRSQLKNNA | 0 | 0 | 2 | 0 | 0 | 1 | 1 | 0 | 2 | 0 | 0 | 0 | 0 | 1 | 0 | 0 | 0 | 0 |
| RIENLNKKVDDGFLDIWTYNAELLVLLENERTLDYHDSNVKNVYEKVRSQLKNNA | 0 | 0 | 0 | 0 | 0 | 0 | 5 | 0 | 1 | 1 | 0 | 0 | 1 | 1 | 0 | 0 | 0 | 0 |
| RIENLNKKVDDGFLDIWTYNAELLVLLENERTLDYHDSNVKNLYENVRSQLKNNA | 0 | 0 | 0 | 0 | 1 | 0 | 0 | 1 | 3 | 0 | 0 | 0 | 1 | 1 | 0 | 0 | 0 | 0 |
| RIENLNKKVDDGFLDIWTYNAELLVLLENERTLDYHDSNVKNLYEKVRSQLKNTA | 0 | 0 | 0 | 1 | 0 | 1 | 1 | 0 | 0 | 1 | 0 | 0 | 0 | 1 | 0 | 0 | 0 | 0 |
| RIENLNKKVDDGFLDIWTYNAELLVLLENERTLDYHDSNVKNLYEKVRCQLKNNA | 0 | 0 | 0 | 1 | 1 | 0 | 0 | 1 | 2 | 0 | 0 | 0 | 1 | 0 | 0 | 0 | 0 | 0 |
| RIENLNKKVDDGFLDIWTYNAELLVLLENERTLDYHDSHVKNLYEKVRSQLKNNA | 0 | 0 | 0 | 0 | 0 | 0 | 1 | 0 | 1 | 0 | 0 | 0 | 0 | 1 | 1 | 0 | 1 | 0 |
| RIENLNKKVDDGFLDIWTYNAELLVLLENERTLAYHDSNVKNLYEKVRSQLKNNA | 0 | 0 | 1 | 0 | 1 | 1 | 0 | 1 | 0 | 2 | 0 | 0 | 0 | 0 | 0 | 0 | 0 | 0 |
| RIENLNKKVDDGFLDIWTYNAELLVLLENDRTLDYHDSNVKNLYEKVRSQLKNNA | 0 | 0 | 0 | 0 | 3 | 1 | 2 | 0 | 1 | 0 | 0 | 0 | 0 | 0 | 0 | 0 | 1 | 0 |
| RIENLNKKVDDGFLDIWTYNAELLVLFENERTLDYHDSNVKNLYEKVRSQLKNNA | 0 | 0 | 0 | 0 | 3 | 0 | 2 | 0 | 1 | 0 | 0 | 1 | 0 | 0 | 0 | 0 | 1 | 0 |
| RIENLNKKVDDGFLDIWTYNAELLDLLENERTLDYHDSNVKNLYEKVRSQLKNNA | 0 | 0 | 0 | 0 | 0 | 1 | 2 | 2 | 3 | 0 | 2 | 0 | 0 | 0 | 0 | 0 | 0 | 0 |
| RIDNLNKKVDDGFLDIWTYNAELLVLLENERTLDYHDSNVKNLYEKVRSQLKNNA | 0 | 0 | 0 | 0 | 5 | 4 | 3 | 0 | 10 | 5 | 0 | 0 | 0 | 0 | 0 | 0 | 0 | 0 |
| RIENLNKRVNDGFLDIWTYNAELLVLLENERTLDYHDSNVKNLYEKVRSQLKNNA | 0 | 0 | 0 | 0 | 0 | 0 | 2 | 1 | 2 | 1 | 0 | 0 | 0 | 0 | 0 | 0 | 0 | 0 |
| RIENLNKKVNDGFLDIWTYNAELLVLLENEGTLDYHDSNVKNLYEKVRSQLKNNA | 0 | 0 | 1 | 0 | 1 | 0 | 1 | 0 | 3 | 0 | 0 | 0 | 0 | 0 | 0 | 0 | 0 | 0 |
| RIENLNKKVDDGFLVIWTYNAELLVLLENERTLDYHDSNVKNLYEKVRSQLKNNA | 1 | 0 | 0 | 0 | 1 | 0 | 1 | 0 | 0 | 0 | 0 | 0 | 1 | 0 | 0 | 0 | 0 | 0 |
| RIENLNKKVDDGFLDSWTYNAELLVLLENERTLDYHDSNVKNLYEKVRSQLKNNA | 0 | 0 | 0 | 0 | 2 | 0 | 1 | 0 | 1 | 0 | 0 | 0 | 0 | 0 | 1 | 0 | 0 | 0 |
| RIENLNKKVDDGFLDNWTYNAELLVLLENERTLDYHDSNVKNLYEKVRSQLKNNA | 0 | 0 | 0 | 0 | 0 | 2 | 1 | 2 | 2 | 0 | 0 | 0 | 0 | 0 | 0 | 0 | 0 | 0 |
| RIENLNKKVDDGFLDLWTYNAELLVLLENERTLDYHDSNVKNLYEKVRSQLKNNA | 1 | 0 | 0 | 0 | 0 | 1 | 0 | 0 | 0 | 0 | 1 | 0 | 0 | 0 | 1 | 0 | 0 | 0 |
| RIENLNKKVDDGFLDIWTYNAELLVLMENERTLDYHDSNVKNLYEKVRSQLKNNA | 0 | 0 | 0 | 0 | 0 | 0 | 0 | 1 | 0 | 0 | 3 | 0 | 0 | 1 | 2 | 0 | 0 | 0 |
| RIENLNKKVDDGFLDIWTYNAELLVLLENERTLDYHDSNVTNLYEKVRSQLKNNA | 0 | 0 | 0 | 0 | 1 | 0 | 3 | 1 | 1 | 0 | 0 | 0 | 0 | 0 | 0 | 0 | 0 | 0 |
| RIENLNKKVDDGFLDIWTYNAELLVLLENERTLDYHDSNVKNLYEKVRSQLKNHA | 0 | 0 | 0 | 0 | 0 | 0 | 2 | 2 | 0 | 1 | 0 | 0 | 0 | 1 | 0 | 0 | 0 | 0 |
| RIENLNKKVDDGFLDIWTYNAELLVLLENERTLDYHDSNVKNLYEKLRSQLKNNA | 0 | 0 | 0 | 2 | 2 | 0 | 1 | 0 | 1 | 0 | 0 | 0 | 0 | 0 | 0 | 0 | 0 | 0 |
| RIENLNKKVDDGFLDIWTYNAELLVLLENERTLDYHDSNVKNIYEKVRSQLKNNA | 0 | 0 | 0 | 0 | 0 | 0 | 0 | 1 | 1 | 0 | 0 | 0 | 0 | 1 | 0 | 0 | 1 | 0 |
| RIENLNKKVDDGFLDIWTYNAELLVLLENERTLDYHDSNVKNFYEKVRSQLKNNA | 0 | 0 | 0 | 2 | 0 | 0 | 2 | 0 | 1 | 0 | 0 | 0 | 0 | 0 | 0 | 0 | 1 | 0 |
| RIENLNKKVDDGFLDIWTYNAELLVLLENERTLDYDDSNVKNLYEKVRSQLKNNA | 0 | 0 | 0 | 0 | 0 | 0 | 1 | 2 | 2 | 1 | 0 | 0 | 0 | 0 | 0 | 0 | 0 | 0 |
| RIENLNKKVDDGFLDIWTYNAELLVLLENERPLDYHDSNVKNLYEKVRSQLKNNA | 0 | 0 | 1 | 0 | 0 | 0 | 0 | 0 | 2 | 0 | 0 | 0 | 0 | 1 | 0 | 1 | 0 | 0 |
| RIENLNKKVDDGFLDIWTYNAELLVLLENEETLDYHDSNVKNLYEKVRSQLKNNA | 0 | 0 | 0 | 0 | 1 | 2 | 0 | 0 | 4 | 0 | 1 | 0 | 0 | 0 | 0 | 0 | 0 | 0 |
| RIENLNKKVDDGFLDIWTYNAELLFLLENERTLDYHDSNVKNLYEKVRSQLKNNA | 0 | 0 | 0 | 0 | 2 | 0 | 0 | 2 | 0 | 0 | 1 | 0 | 0 | 1 | 0 | 0 | 0 | 0 |
| RIENLNKKVDDGFLDIWTYNAELFVLLENERTLDYHDSNVKNLYEKVRSQLKNNA | 0 | 0 | 0 | 0 | 0 | 1 | 0 | 0 | 1 | 0 | 0 | 0 | 0 | 1 | 0 | 0 | 0 | 1 |
| RIENLNKKVDDGFLDIWPYNAELLVLLENERTLDYHDSNVKNLYEKVRSQLKNNA | 0 | 0 | 0 | 1 | 2 | 0 | 1 | 0 | 0 | 0 | 0 | 0 | 0 | 1 | 0 | 0 | 0 | 0 |
| RIENLNKKVDDGFLDIWNYNAELLVLLENERTLDYHDSNVKNLYEKVRSQLKNNA | 0 | 1 | 1 | 0 | 1 | 0 | 0 | 0 | 2 | 0 | 0 | 0 | 0 | 0 | 0 | 0 | 0 | 0 |
| RIENLNKKVDDGFLDIGTYNAELLVLLENERTLDYHDSNVKNLYEKVRSQLKNNA | 0 | 0 | 0 | 0 | 0 | 0 | 2 | 0 | 3 | 0 | 2 | 0 | 0 | 1 | 0 | 0 | 0 | 0 |
| RIENLNKKVDAGFLDIWTYNAELLVLLENERTLDYHDSNVKNLYEKVRSQLKNNA | 0 | 0 | 0 | 0 | 2 | 1 | 1 | 1 | 0 | 0 | 0 | 0 | 0 | 0 | 0 | 0 | 0 | 0 |
| RIENLNKKDDDGFLDIWTYNAELLVLLENERTLDYHDSNVKNLYEKVRSQLKNNA | 0 | 0 | 1 | 0 | 0 | 0 | 0 | 0 | 0 | 0 | 1 | 0 | 0 | 1 | 0 | 1 | 0 | 0 |
| RIENLHKKVDDGFLDIWTYNAELLVLLENERTLDYHDSNVKNLYEKVRSQLKNNA | 0 | 0 | 0 | 0 | 1 | 0 | 0 | 3 | 1 | 1 | 0 | 0 | 0 | 0 | 0 | 0 | 0 | 0 |
| RIENINKKVDDGFLDIWTYNAELLVLLENERTLDYHDSNVKNLYEKVRSQLKNNA | 0 | 1 | 1 | 0 | 1 | 0 | 0 | 1 | 0 | 0 | 0 | 0 | 0 | 0 | 0 | 0 | 0 | 0 |
| EIENLNKKVDDGFLDIWTYNAELLVLLENERTLDYHDSNVKNLYEKVRSQLKNNA | 0 | 0 | 0 | 0 | 1 | 0 | 2 | 0 | 0 | 1 | 2 | 0 | 0 | 0 | 0 | 0 | 0 | 0 |
| SIENLNKKVDDGFLDIWTYNAELLVLLENERTLDYHDSNVKNLYEKVRSQLKNNA | 0 | 0 | 0 | 0 | 0 | 0 | 1 | 1 | 1 | 0 | 0 | 0 | 0 | 0 | 0 | 0 | 0 | 0 |
| RIENLNKKVVDGFLDIWTYNAELLVLLENERTLDYHDSNVKNLYEKVRSQLKNNA | 0 | 0 | 0 | 0 | 0 | 1 | 1 | 1 | 0 | 0 | 0 | 0 | 0 | 0 | 0 | 0 | 0 | 0 |
| RIENLNKKVNDGFLDIWTYNAELLVLLENERTLGYHDSNVKNLYEKVRSQLKNNA | 0 | 0 | 0 | 0 | 0 | 0 | 1 | 1 | 1 | 0 | 0 | 0 | 0 | 0 | 0 | 0 | 0 | 0 |
| RIENLNKKVNDGFLDIWTYNAELLVLLENERTLDYHDSNVKNLCEKVRSQLKNNA | 0 | 0 | 0 | 0 | 0 | 0 | 3 | 1 | 2 | 0 | 0 | 0 | 0 | 0 | 0 | 0 | 0 | 0 |
| RIENLNKKVNDGFLDIWTYNAELLVLLENERTFDYHDSNVKNLYEKVRSQLKNNA | 1 | 0 | 0 | 0 | 0 | 0 | 1 | 1 | 0 | 0 | 0 | 0 | 0 | 0 | 0 | 0 | 0 | 0 |
| RIENLNKKVNDGFLDIWTYNAELLVLLENERALDYHDSNVKNLYEKVRSQLKNNA | 0 | 0 | 1 | 0 | 1 | 0 | 0 | 1 | 0 | 0 | 0 | 0 | 0 | 0 | 0 | 0 | 0 | 0 |
| RIENLNKKVNDGFLDIWTYNAELLVLLENEKTLDYHDSNVKNLYEKVRSQLKNNA | 0 | 0 | 0 | 1 | 0 | 0 | 1 | 0 | 1 | 0 | 0 | 0 | 0 | 0 | 0 | 0 | 0 | 0 |
| RIENLNKKVNDGFLDIWTYNAELLVLLEKERTLDYHDSNVKNLYEKVRSQLKNNA | 0 | 0 | 0 | 0 | 0 | 0 | 1 | 1 | 1 | 0 | 0 | 0 | 0 | 0 | 0 | 0 | 0 | 0 |
| RIENLNKKVDDGFQDIWTYNAELLVLLENERTLDYHDSNVKNLYEKVRSQLKNNA | 0 | 0 | 0 | 0 | 0 | 0 | 0 | 2 | 1 | 0 | 0 | 0 | 1 | 0 | 0 | 0 | 0 | 0 |
| RIENLNKKVDDGFLDIWTYTAELLVLLENERTLDYHDSNVKNLYEKVRSQLKNNA | 0 | 0 | 0 | 0 | 0 | 1 | 1 | 0 | 0 | 0 | 1 | 0 | 0 | 0 | 0 | 0 | 0 | 0 |
| RIENLNKKVDDGFLDIWTYSAELLVLLENERTLDYHDSNVKNLYEKVRSQLKNNA | 0 | 0 | 0 | 0 | 0 | 1 | 4 | 0 | 2 | 0 | 0 | 0 | 0 | 0 | 0 | 0 | 0 | 0 |
| RIENLNKKVDDGFLDIWTYNVELLVLLENERTLDYHDSNVKNLYERVRSQLKNNA | 0 | 0 | 0 | 0 | 0 | 2 | 0 | 2 | 2 | 0 | 0 | 0 | 0 | 0 | 0 | 0 | 0 | 0 |
| RIENLNKKVDDGFLDIWTYNAEVLVLLENERTLDYHDSNVKNLYEKVRSQLKNNA | 0 | 0 | 1 | 0 | 0 | 0 | 0 | 1 | 1 | 0 | 0 | 0 | 0 | 0 | 0 | 0 | 0 | 0 |
| RIENLNKKVDDGFLDIWTYNAELVVLLENERTLDYHDSNVKNLYEKVRSQLKNNA | 0 | 0 | 1 | 0 | 0 | 2 | 1 | 0 | 0 | 0 | 0 | 0 | 0 | 0 | 0 | 0 | 0 | 0 |
| RIENLNKKVDDGFLDIWTYNAELLVLWENERTLDYHDSNVKNLYEKVRSQLKNNA | 1 | 1 | 0 | 0 | 0 | 0 | 1 | 0 | 0 | 0 | 0 | 0 | 0 | 0 | 0 | 0 | 0 | 0 |
| RIENLNKKVDDGFLDIWTYNAELLVLLENERTLDYHDSYVKNLYEKVRSQLKNNA | 0 | 0 | 1 | 0 | 0 | 0 | 0 | 0 | 1 | 1 | 0 | 0 | 0 | 0 | 0 | 0 | 0 | 0 |
| RIENLNKKVDDGFLDIWTYNAELLVLLENERTLDYHDSNVQNLYEKVRSQLKNNA | 0 | 0 | 13 | 0 | 0 | 0 | 0 | 1 | 1 | 0 | 0 | 0 | 0 | 0 | 0 | 0 | 0 | 0 |
| RIENLNKKVDDGFLDIWTYNAELLVLLENERTLDYHDSNVKTLYEKVRSQLKNNA | 1 | 0 | 1 | 0 | 0 | 0 | 1 | 0 | 0 | 0 | 0 | 0 | 0 | 0 | 0 | 0 | 0 | 0 |
| RIENLNKKVDDGFLDIWTYNAELLVLLENERTLDYHDSNVKNLYETVRSQLKNNA | 0 | 0 | 0 | 0 | 0 | 0 | 0 | 0 | 2 | 1 | 0 | 0 | 0 | 0 | 0 | 0 | 0 | 1 |
| RIENLNKKVDDGFLDIWTYNAELLVLLENERTLDYHDSNVKNLYERVRNQLKNNA | 0 | 1 | 0 | 0 | 0 | 0 | 1 | 0 | 0 | 1 | 0 | 0 | 0 | 0 | 0 | 0 | 0 | 0 |
| RIENLNKKVDDGFLDIWTYNAELLVLLENERTLDYHDSNVKNLYEKVRSQVKNNA | 0 | 0 | 0 | 0 | 9 | 0 | 0 | 1 | 0 | 0 | 0 | 0 | 0 | 0 | 0 | 1 | 0 | 0 |
| RIENLNKKVDDGFLDIWTYNAELLVLLENERTLDYHDSNVKNLYEKVRSQLKKNA | 0 | 0 | 0 | 0 | 0 | 0 | 0 | 0 | 1 | 1 | 0 | 0 | 2 | 0 | 0 | 0 | 0 | 0 |
| RIENLNKKVDDGFLDIWTYNAELLVLLENERTLDYHDSNVKNLYEKVRSQFKNNA | 0 | 0 | 0 | 0 | 1 | 0 | 1 | 1 | 0 | 0 | 0 | 0 | 0 | 0 | 0 | 0 | 0 | 0 |
| RIENLNKKVDDGFLDIWTYNAELLVLLENERTLDYHDSNVKNLYEKVRIQLKNNA | 0 | 0 | 4 | 0 | 0 | 0 | 2 | 0 | 0 | 0 | 0 | 2 | 0 | 0 | 0 | 0 | 0 | 0 |
| RIENLNKKVDDGFLDIWTYNAELLVLLENERTLDYHDSNVKNLYEKGRSQLKNNA | 0 | 0 | 0 | 0 | 2 | 0 | 0 | 0 | 0 | 1 | 1 | 0 | 0 | 0 | 0 | 0 | 0 | 0 |
| RIENLNKKVDDGFLDIWTYNAELLVLLENERTLDYHDSNVKNLNEKVRSQLKNNA | 0 | 0 | 0 | 0 | 0 | 0 | 1 | 0 | 0 | 0 | 2 | 2 | 0 | 0 | 0 | 0 | 0 | 0 |
| RIENLNKKVDDGFLDIWTYNAELLVLLENERTLDSHDSNVKNLYEKVRSQLKNNA | 0 | 0 | 0 | 0 | 1 | 0 | 5 | 0 | 3 | 0 | 0 | 0 | 0 | 0 | 0 | 0 | 0 | 0 |
| RIENLNKKVDDGFLDIWTYNAELLVLLENARTLDYHDSNVKNLYEKVRSQLKNNA | 0 | 0 | 1 | 0 | 0 | 0 | 0 | 0 | 1 | 0 | 0 | 0 | 0 | 1 | 0 | 0 | 0 | 0 |
| RIENLNKKVDDGFLDIWTYNAELLVLLEIERTLDYHDSNVKNLYEKVRSQLKNNA | 0 | 0 | 0 | 0 | 0 | 0 | 1 | 2 | 1 | 0 | 0 | 0 | 0 | 0 | 0 | 0 | 0 | 0 |
| RIENLNKKVDDGFLDIWTYNAELLVLLDNERTLDYHDSNVKNLYEKVRSQLKNNA | 0 | 0 | 0 | 0 | 0 | 0 | 2 | 0 | 1 | 0 | 0 | 0 | 0 | 0 | 1 | 0 | 0 | 0 |
| RIENLNKKVDDGFLDIWTYNAALLVLLENERTLDYHDSNVKNLYEKVRSQLKNNA | 0 | 0 | 0 | 0 | 1 | 0 | 1 | 0 | 0 | 0 | 0 | 0 | 0 | 0 | 0 | 0 | 0 | 1 |
| RIENLNKKVDDGFLDIWSYNAELLVLLENERTLDYHDSNVKNLYEKVRSQLKNNA | 0 | 0 | 0 | 0 | 1 | 0 | 1 | 0 | 0 | 0 | 0 | 0 | 0 | 0 | 0 | 1 | 0 | 0 |
| RIENLNKKVDDGFLDICTYNAELLVLLENERTLDYHDSNVKNLYEKVRSQLKNNA | 0 | 0 | 0 | 0 | 0 | 0 | 0 | 0 | 2 | 0 | 0 | 0 | 0 | 1 | 3 | 0 | 0 | 0 |
| RIENLNKKVDDGFLAIWTYNAELLVLLENERTLDYHDSNVKNLYEKVRSQLKNNA | 0 | 0 | 0 | 0 | 4 | 0 | 0 | 0 | 1 | 0 | 0 | 0 | 0 | 0 | 0 | 0 | 0 | 1 |
| GIENLNKKVDDGFLDIWTYNVELLVLLENERTLDYHDSNVKNLYEKVRSQLKNNA | 0 | 0 | 0 | 0 | 1 | 0 | 0 | 1 | 1 | 0 | 0 | 0 | 0 | 0 | 0 | 0 | 0 | 0 |
| RIGNLNKKVNDGFLDIWTYNAELLVLLENERTLDYHDSNVKNLYEKVRSQLKNNA | 0 | 0 | 1 | 0 | 0 | 0 | 0 | 0 | 1 | 0 | 0 | 0 | 0 | 0 | 0 | 0 | 0 | 0 |
| RIEYLNKKVNDGFLDIWTYNAELLVLLENERTLDYHDSNVKNLYEKVRSQLKNNA | 0 | 0 | 0 | 0 | 0 | 0 | 1 | 0 | 1 | 0 | 0 | 0 | 0 | 0 | 0 | 0 | 0 | 0 |
| RIEYLNKKVDDGFLDIWTYNAELLVLLENGRTLDYHDSNVKNLYEKVRSQLKNNA | 0 | 0 | 0 | 0 | 0 | 0 | 0 | 1 | 1 | 0 | 0 | 0 | 0 | 0 | 0 | 0 | 0 | 0 |
| RIENLN?KVNDGFLDIWTYNAELLVLLENERTLDYHDSNVKNLYEKVRSQLKNNA | 0 | 0 | 0 | 0 | 0 | 0 | 0 | 1 | 1 | 0 | 0 | 0 | 0 | 0 | 0 | 0 | 0 | 0 |
| RIENLNKRVDDGFLDIWTYNVELLVLLENERTLDYHDSNVKNLYEKVRSQLKNNA | 0 | 0 | 1 | 0 | 0 | 0 | 1 | 0 | 0 | 0 | 0 | 0 | 0 | 0 | 0 | 0 | 0 | 0 |
| RIENLNKRVDDGFLDIWTYNAELLVLLENERTLDYHDSNVKNLYERVRSQLKNNA | 0 | 0 | 0 | 0 | 0 | 0 | 0 | 1 | 1 | 0 | 0 | 0 | 0 | 0 | 0 | 0 | 0 | 0 |
| RIENLNKQVDDGFLDIWTYNAELLVLLENERTLDYHDSNVKNLYEKVRSQLKNNA | 0 | 0 | 0 | 0 | 0 | 1 | 0 | 0 | 0 | 0 | 0 | 0 | 0 | 1 | 0 | 0 | 0 | 0 |
| RIENLNKKVNGGFLDIWTYNAELLVLLENERTLDYHDSNVKNLYEKVRSQLKNNA | 0 | 0 | 0 | 0 | 0 | 0 | 1 | 0 | 1 | 0 | 0 | 0 | 0 | 0 | 0 | 0 | 0 | 0 |

| | | | | | | | | | | | | | | | | | | |
|---|---|---|---|---|---|---|---|---|---|---|---|---|---|---|---|---|---|---|
| RIENLNKKVNEGFLDIWTYNAELLVLLENERTLDYHDSNVKNLYEKVRSQLKNNA | 0 | 0 | 0 | 0 | 0 | 0 | 0 | 0 | 1 | 0 | 0 | 0 | 0 | 0 | 0 | 0 | 1 | 0 |
| RIENLNKKVNDGSLDIWTYNAELLVLLENERTLDYHDSNVKNLYEKVRSQLKNNA | 0 | 0 | 0 | 0 | 0 | 0 | 2 | 0 | 1 | 0 | 0 | 0 | 0 | 0 | 0 | 0 | 0 | 0 |
| RIENLNKKVNDGFLGIWTYNAELLVLLENERTLDYHDSNVKNLYEKVRSQLKNNA | 0 | 0 | 0 | 0 | 0 | 0 | 2 | 0 | 2 | 0 | 0 | 0 | 0 | 0 | 0 | 0 | 0 | 0 |
| RIENLNKKVNDGFLDVWTYNAELLVLLENERTLDYHDSNVKNLYEKVRSQLKNNA | 0 | 1 | 0 | 0 | 0 | 0 | 1 | 0 | 0 | 0 | 0 | 0 | 0 | 0 | 0 | 0 | 0 | 0 |
| RIENLNKKVNDGFLDIWTYNAELLVLLKNERTLDYHDSNVKNLYEKVRSQLKNNA | 0 | 0 | 0 | 0 | 0 | 0 | 0 | 1 | 3 | 0 | 0 | 0 | 0 | 0 | 0 | 0 | 0 | 0 |
| RIENLNKKVNDGFLDIWTYNAELLVLLENKRTLDYHDSNVKNLYEKVRSQLKNNA | 0 | 0 | 0 | 0 | 1 | 0 | 0 | 0 | 1 | 0 | 0 | 0 | 0 | 0 | 0 | 0 | 0 | 0 |
| RIENLNKKVNDGFLDIWTYNAELLVLLENERTLDYPDSNVKNLYEKVRSQLKNNA | 0 | 0 | 0 | 1 | 1 | 0 | 0 | 0 | 0 | 0 | 0 | 0 | 0 | 0 | 0 | 0 | 0 | 0 |
| RIENLNKKVNDGFLDIWTYNAELLVLLENERTLDYHNSNVKNLYEKVRSQLKNNA | 0 | 0 | 0 | 0 | 1 | 0 | 0 | 0 | 4 | 0 | 0 | 0 | 0 | 0 | 0 | 0 | 0 | 0 |
| RIENLNKKVNDGFLDIWTYNAELLVLLENERTLDYHDSNVKNLYGKVRSQLKNNA | 0 | 0 | 0 | 0 | 0 | 0 | 1 | 0 | 0 | 1 | 0 | 0 | 0 | 0 | 0 | 0 | 0 | 0 |
| RIENLNKKVNDGFLDIWTYNAELLVLLENERTLDYHDSNVKNLYEKVRSQLRNNA | 0 | 0 | 0 | 0 | 0 | 0 | 1 | 0 | 1 | 0 | 0 | 0 | 0 | 0 | 0 | 0 | 0 | 0 |
| RIENLNKKVNDGFLDIWTYNAELLVLLENERTLDYHDSNVKNLYEKVGSQLKNNA | 0 | 0 | 0 | 0 | 1 | 0 | 0 | 1 | 0 | 0 | 0 | 0 | 0 | 0 | 0 | 0 | 0 | 0 |
| RIENLNKKVNDGFLDIWTYNAELLVLLENERTLDYHDSKVKNLYEKVRSQLKNNA | 0 | 0 | 0 | 0 | 0 | 0 | 0 | 0 | 1 | 1 | 0 | 0 | 0 | 0 | 0 | 0 | 0 | 0 |
| RIENLNKKVNDGFLDIWTYNAELLVLLEDERTLDYHDSNVKNLYEKVRSQLKNNA | 0 | 0 | 0 | 0 | 0 | 1 | 2 | 0 | 0 | 0 | 0 | 0 | 0 | 0 | 0 | 0 | 0 | 0 |
| RIENLNKKVNDGFLDIWTYDAELLVLLENERTLDYHDSNVKNLYEKVRSQLKNNA | 0 | 0 | 0 | 0 | 0 | 1 | 1 | 0 | 0 | 0 | 0 | 0 | 0 | 0 | 0 | 0 | 0 | 0 |
| RIENLNKKVNDGFLDIWAYNAELLVLLENERTLDYHDSNVKNLYEKVRSQLKNNA | 0 | 0 | 0 | 0 | 0 | 0 | 2 | 1 | 0 | 0 | 0 | 0 | 0 | 0 | 0 | 0 | 0 | 0 |
| RIENLNKKVNDGFLDIRTYNAELLVLLENERTLDYHDSNVKNLYEKVRSQLKNNA | 0 | 0 | 0 | 0 | 0 | 1 | 0 | 0 | 1 | 0 | 0 | 0 | 0 | 0 | 0 | 0 | 0 | 0 |
| RIENLNKKVDEGFLDIWTYNAELLVLLENERTLDYHDSNVKNLYEKVRSQLKNNA | 0 | 0 | 0 | 0 | 2 | 0 | 2 | 0 | 0 | 0 | 0 | 0 | 0 | 0 | 0 | 0 | 0 | 0 |
| RIENLNKKVDDGFVDIWTYNAELLVLLENERTLDYHDSNVKNLYEKVRSQLKNNA | 0 | 0 | 0 | 0 | 0 | 0 | 0 | 1 | 0 | 0 | 0 | 0 | 2 | 0 | 0 | 0 | 0 | 0 |
| RIENLNKKVDDGFLDIWTYYAELLVLLENERTLDYHDSNVKNLYEKVRSQLKNNA | 0 | 0 | 0 | 1 | 0 | 0 | 0 | 0 | 2 | 0 | 0 | 0 | 0 | 0 | 0 | 0 | 0 | 0 |
| RIENLNKKVDDGFLDIWTYNVELLVLLENERTLGYHDSNVKNLYEKVRSQLKNNA | 0 | 0 | 0 | 0 | 0 | 0 | 0 | 1 | 1 | 0 | 0 | 0 | 0 | 0 | 0 | 0 | 0 | 0 |
| RIENLNKKVDDGFLDIWTYNDELLVLLENERTLDYHDSNVKNLYEKVRSQLKNNA | 0 | 0 | 0 | 0 | 0 | 0 | 1 | 1 | 0 | 0 | 0 | 0 | 0 | 0 | 0 | 0 | 0 | 0 |
| RIENLNKKVDDGFLDIWTYNAQLLVLLENERTLDYHDSNVKNLYEKVRSQLKNNA | 1 | 0 | 1 | 0 | 0 | 0 | 0 | 0 | 0 | 0 | 0 | 0 | 0 | 0 | 0 | 0 | 0 | 0 |
| RIENLNKKVDDGFLDIWTYNAEQLVLLENERTLDYHDSNVKNLYEKVRSQLKNNA | 0 | 0 | 0 | 0 | 0 | 1 | 1 | 0 | 0 | 0 | 0 | 0 | 0 | 0 | 0 | 0 | 0 | 0 |
| RIENLNKKVDDGFLDIWTYNAELLVQLENERTLDYHDSNVKNLYEKVRSQLKNNA | 0 | 0 | 0 | 0 | 0 | 0 | 0 | 1 | 0 | 0 | 0 | 0 | 1 | 0 | 0 | 0 | 0 | 0 |
| RIENLNKKVDDGFLDIWTYNAELLVLLEYERTLDYHDSNVKNLYEKVRSQLKNNA | 0 | 0 | 0 | 0 | 0 | 0 | 0 | 0 | 1 | 0 | 0 | 0 | 1 | 0 | 0 | 0 | 0 | 0 |
| RIENLNKKVDDGFLDIWTYNAELLVLLENERTMDYHDSNVKNLYEKVRSQLKNNA | 0 | 0 | 0 | 0 | 0 | 0 | 0 | 1 | 1 | 0 | 0 | 0 | 0 | 0 | 0 | 0 | 0 | 0 |
| RIENLNKKVDDGFLDIWTYNAELLVLLENERTLVYHDSNVKNLYEKVRSQLKNNA | 0 | 0 | 0 | 0 | 0 | 0 | 0 | 0 | 2 | 0 | 0 | 0 | 0 | 1 | 0 | 0 | 0 | 0 |
| RIENLNKKVDDGFLDIWTYNAELLVLLENERTLGYHDSNVKNLYEKVRNQLKNNA | 0 | 0 | 0 | 0 | 0 | 0 | 1 | 0 | 0 | 0 | 0 | 1 | 0 | 0 | 0 | 0 | 0 | 0 |
| RIENLNKKVDDGFLDIWTYNAELLVLLENERTLDYLDSNVKNLYEKVRSQLKNNA | 0 | 0 | 0 | 0 | 0 | 0 | 0 | 1 | 1 | 0 | 0 | 0 | 0 | 0 | 0 | 0 | 0 | 0 |
| RIENLNKKVDDGFLDIWTYNAELLVLLENERTLDYHDSNVNNLYEKVRSQLKNNA | 0 | 0 | 0 | 0 | 0 | 0 | 2 | 0 | 0 | 0 | 1 | 0 | 0 | 0 | 0 | 0 | 0 | 0 |
| RIENLNKKVDDGFLDIWTYNAELLVLLENERTLDYHDSNVKYLYEKVRSQLKNNA | 0 | 0 | 0 | 0 | 0 | 0 | 0 | 0 | 0 | 0 | 0 | 0 | 1 | 1 | 0 | 0 | 0 | 0 |
| RIENLNKKVDDGFLDIWTYNAELLVLLENERTLDYHDSNVKNLYEKVRSQLKINA | 0 | 1 | 0 | 0 | 0 | 0 | 0 | 0 | 0 | 0 | 1 | 0 | 0 | 0 | 0 | 0 | 0 | 0 |
| RIENLNKKVDDGFLDIWTYNAELLVLLENERTLDYHDSNVKNLYEKVRSQLINNA | 0 | 0 | 0 | 0 | 0 | 0 | 0 | 0 | 0 | 0 | 0 | 0 | 0 | 1 | 1 | 0 | 0 | 0 |
| RIENLNKKVDDGFLDIWTYNAELLVLLENERTLDYHDSNVKNLYEKVRSLLKNNA | 1 | 0 | 0 | 0 | 0 | 0 | 0 | 3 | 0 | 0 | 0 | 0 | 0 | 0 | 0 | 0 | 0 | 0 |
| RIENLNKKVDDGFLDIWTYNAELLVLLENERTLDYHDSNVKNLYEKVRSKLKNNA | 0 | 0 | 0 | 0 | 0 | 0 | 1 | 1 | 0 | 0 | 0 | 0 | 0 | 0 | 0 | 0 | 0 | 0 |
| RIENLNKKVDDGFLDIWTYNAELLVLLENERTLDYHDSNVKNLYARVRSQLKNNA | 0 | 0 | 0 | 0 | 0 | 0 | 0 | 1 | 0 | 0 | 0 | 0 | 1 | 0 | 0 | 0 | 0 | 0 |
| RIENLNKKVDDGFLDIWTYNAELLVLLENERTLDYHDSNVKHLYEKVRSQLKNNA | 0 | 0 | 0 | 0 | 1 | 0 | 0 | 0 | 1 | 0 | 0 | 0 | 0 | 0 | 0 | 0 | 0 | 0 |
| RIENLNKKVDDGFLDIWTYNAELLVLLENERTLDYHDSNGKNLYEKVRSQLKNNA | 0 | 0 | 0 | 0 | 0 | 0 | 0 | 0 | 1 | 0 | 0 | 0 | 1 | 0 | 0 | 0 | 0 | 0 |
| RIENLNKKVDDGFLDIWTYNAELLVLLENERSLDYHDSNVKNLYEKVRSQLKNNA | 0 | 0 | 0 | 0 | 1 | 0 | 0 | 0 | 1 | 0 | 0 | 0 | 0 | 0 | 0 | 0 | 0 | 0 |
| RIENLNKKVDDGFLDIWTYNAELLVLLENERNLDYHDSNVKNLYEKVRNQLKNNA | 0 | 0 | 0 | 0 | 0 | 0 | 0 | 1 | 1 | 0 | 0 | 0 | 0 | 0 | 0 | 0 | 0 | 0 |
| RIENLNKKVDDGFLDIWTYNAELLILLENERTLDHHDSNVKNLYEKVRSQLKNNA | 0 | 0 | 0 | 0 | 0 | 1 | 0 | 3 | 0 | 0 | 0 | 0 | 0 | 0 | 0 | 0 | 0 | 0 |
| RIENLNKKVDDGFLDIWTSNAELLVLLENERTLDYHDSNVKNLYEKVRSQLKNNA | 0 | 0 | 1 | 0 | 0 | 0 | 0 | 0 | 1 | 0 | 0 | 0 | 0 | 0 | 0 | 0 | 0 | 0 |
| RIENLNKKVDDGFLDIWTNNAELLVLLENERTLDYHDSNVKNLYEKVRSQLKNNA | 0 | 0 | 0 | 0 | 0 | 0 | 1 | 0 | 0 | 0 | 0 | 1 | 0 | 0 | 0 | 0 | 0 | 0 |
| RIENLNKKVDDGFLDIWTFNAELLVLLENERTLDYHDSNVKNLYEKVRSQLKNNA | 0 | 1 | 0 | 0 | 0 | 0 | 0 | 1 | 0 | 0 | 0 | 0 | 0 | 0 | 0 | 0 | 0 | 0 |
| RIENLNKKGDDGFLDIWTYNAELLVLLENERTLDYHDSNVKNLYEKVRSQLKNNA | 0 | 0 | 0 | 0 | 2 | 0 | 3 | 0 | 0 | 0 | 0 | 0 | 0 | 0 | 0 | 0 | 0 | 0 |
| RIENLNKKFDDGFLDIWTYNAELLVLLENERTLDYHDSNVKNLYEKVRSQLKNNA | 0 | 0 | 0 | 0 | 0 | 0 | 0 | 0 | 1 | 0 | 1 | 0 | 0 | 0 | 0 | 0 | 0 | 0 |
| RIENLNKEVNDGFLDIWTYNAELLVLLENERTLDYHDSNVKNLYEKVRSQLKNNA | 0 | 0 | 0 | 0 | 0 | 1 | 1 | 0 | 0 | 0 | 0 | 0 | 0 | 0 | 0 | 0 | 0 | 0 |
| RIENLKKKVDDGFLDIWTYNAELLVLLENERTLDYHDSNVKNLYEKVRSQLKNNA | 0 | 0 | 0 | 0 | 1 | 1 | 0 | 0 | 0 | 0 | 0 | 0 | 0 | 0 | 0 | 0 | 0 | 0 |
| RIANLNKKVDDGFLDIWTYNAELLVLLENERTLDYHDSNVKNLYEKVRSQLKNNA | 0 | 0 | 0 | 0 | 0 | 1 | 0 | 0 | 0 | 0 | 0 | 0 | 0 | 0 | 0 | 0 | 1 | 0 |
| GIENLNKKVDDGFLDIWTYNAELLVLLENERTLDYHDSNVKNLYEKVRNQLKNNA | 1 | 0 | 0 | 0 | 0 | 1 | 0 | 0 | 0 | 0 | 0 | 0 | 0 | 0 | 0 | 0 | 0 | 0 |
| RVENLNKKVDDGFLDIWTYNTELLVLLENERTLDYHDSNVKNLYEKVRSQLKNNA | 0 | 0 | 0 | 0 | 1 | 0 | 0 | 0 | 0 | 0 | 0 | 0 | 0 | 0 | 0 | 0 | 0 | 0 |
| RVENLNKKVDDGFLDIWTYNAELLVLLENERTLDYHDSNVKNLYEKVRNQLKNNA | 0 | 0 | 0 | 0 | 0 | 0 | 0 | 0 | 1 | 0 | 0 | 0 | 0 | 0 | 0 | 0 | 0 | 0 |
| RTENSNKKVDDGFLDIRTYNAELLVPLENERTLDYHDSNAKNLYEKVRSQLKNNA | 0 | 0 | 0 | 0 | 0 | 0 | 0 | 0 | 0 | 2 | 0 | 0 | 0 | 0 | 0 | 0 | 0 | 0 |
| RTENLNKKVNDGFLDIWTYNAELLVLLENERTLDYHDSNVKNLYEKVRSQLKNNA | 0 | 0 | 1 | 0 | 0 | 0 | 0 | 0 | 0 | 0 | 0 | 0 | 0 | 0 | 0 | 0 | 0 | 0 |
| RTENLNKKVDDGFLDIWTYNVELLVLLENERTLDYHDSNVKNLYEKVRSQLKNNA | 0 | 0 | 0 | 0 | 0 | 0 | 0 | 2 | 0 | 0 | 0 | 0 | 0 | 0 | 0 | 0 | 0 | 0 |
| RTENLNKKVDDGFLDIWTYNAELLVLLENERTLDYHDSNVKNLYEKVRNQLKNNA | 0 | 0 | 0 | 0 | 0 | 0 | 0 | 0 | 0 | 0 | 0 | 0 | 0 | 1 | 0 | 0 | 0 | 0 |
| RTENLNKKVDDGFLDIWTYNAELLVLLENERTLDYHDSNVKNLYEKVRGQLKNNA | 0 | 0 | 0 | 0 | 1 | 0 | 0 | 0 | 0 | 0 | 0 | 0 | 0 | 0 | 0 | 0 | 0 | 0 |
| RMENLNKKVNDGFLDIWTYNAELLVLLENERTLDYHDSNVKNLYEKVRSQLKNNA | 0 | 0 | 0 | 0 | 0 | 0 | 0 | 0 | 1 | 0 | 0 | 0 | 0 | 0 | 0 | 0 | 0 | 0 |
| RIKNLNKKVDDGFLDIWTYNAELSVLLENERTLDYHDSNVKNLYEKVRNQLKNNA | 0 | 0 | 0 | 0 | 1 | 0 | 0 | 0 | 0 | 0 | 0 | 0 | 0 | 0 | 0 | 0 | 0 | 0 |
| RIGNLNKKVDDGFLDIWTYNAELLVLLENERTLDYHDSNVKNLYEKIRSQLKNNA | 0 | 0 | 0 | 1 | 0 | 0 | 0 | 0 | 0 | 0 | 0 | 0 | 0 | 0 | 0 | 0 | 0 | 0 |
| RIEYLNKKVDDGFLDIWTYNAELLVLLENERTLNYHDSNVKNLYEKVRSQLKNNA | 0 | 0 | 0 | 0 | 0 | 0 | 0 | 0 | 1 | 0 | 0 | 0 | 0 | 0 | 0 | 0 | 0 | 0 |
| RIEYLNKKVDDGFLDIWTYHAELLVLLENERTLDYHDSNVKNLYEKVRSQLKNNA | 0 | 0 | 0 | 0 | 0 | 1 | 0 | 0 | 0 | 0 | 0 | 0 | 0 | 0 | 0 | 0 | 0 | 0 |
| RIESLNKKVNDGFLDIWTYNAELLVLLENERTLDYHDSNVKNLYEKVRSQLKNNA | 0 | 0 | 0 | 0 | 0 | 0 | 0 | 0 | 1 | 0 | 0 | 0 | 0 | 0 | 0 | 0 | 0 | 0 |
| RIESLNKKVNDGFLDIWTYNAELLVLLENERALDYHDSNVKNLYEKVRSQLKNNA | 0 | 0 | 0 | 0 | 0 | 0 | 0 | 0 | 1 | 0 | 0 | 0 | 0 | 0 | 0 | 0 | 0 | 0 |
| RIESLNKKVDDGFLDIWTYNAELLVLLENERTLDYHDSNVKNLYEKVRNQLKNNA | 0 | 0 | 0 | 0 | 0 | 0 | 0 | 0 | 1 | 0 | 0 | 0 | 0 | 0 | 0 | 0 | 0 | 0 |
| RIENSNKKVNDGFLDIWTYNAELLVLLENERTLDYHDSNVKNLYEKVRSQLKNNA | 0 | 0 | 0 | 0 | 1 | 0 | 0 | 0 | 0 | 0 | 0 | 0 | 0 | 0 | 0 | 0 | 0 | 0 |
| RIENSNKKVDDGFLDIWTYNAELSVLLENERTLDYHDSNVKNLYEKVRSQLKNNA | 0 | 0 | 0 | 0 | 0 | 0 | 0 | 0 | 0 | 0 | 0 | 0 | 1 | 0 | 0 | 0 | 0 | 0 |
| RIENSNKKVDDGFLDIWTYNAELLVPLENERTLDYHDSNVKNLYEKVRSQLKNNA | 0 | 0 | 0 | 0 | 0 | 0 | 0 | 1 | 0 | 0 | 0 | 0 | 0 | 0 | 0 | 0 | 0 | 0 |
| RIENSNKKVDDGFLDIWTYNAELLVPLENERTLDHHDSNVKNLYEKVRSQLKNNA | 0 | 0 | 0 | 0 | 0 | 0 | 0 | 0 | 0 | 0 | 1 | 0 | 0 | 0 | 0 | 0 | 0 | 0 |
| RIENSNKKVDDGFLDIWTYNAELLVLLENERTLDYHDSNVKNLYEKVRSQLKNSA | 0 | 0 | 0 | 0 | 0 | 0 | 0 | 1 | 0 | 0 | 0 | 0 | 0 | 0 | 0 | 0 | 0 | 0 |
| RIENSNKKVDDGFLDIWTYNAELLALLENERTLDYHDSNVKNLYEKVRSQLKNNA | 0 | 0 | 0 | 0 | 0 | 0 | 0 | 0 | 0 | 1 | 0 | 0 | 0 | 0 | 0 | 0 | 0 | 0 |
| RIENSNKKVDDGFLDIWTHNAELLVPLENERTLDYHDSNVKNLYEKVRSQLKNNA | 0 | 0 | 0 | 0 | 0 | 0 | 0 | 0 | 0 | 1 | 0 | 0 | 0 | 0 | 0 | 0 | 0 | 0 |
| RIENPNKKVDDGFLDIWTYNAELLVPLENERTLDYHDSNVKNLYEKVRSQLKNNA | 0 | 0 | 0 | 0 | 0 | 0 | 0 | 0 | 1 | 0 | 0 | 0 | 0 | 0 | 0 | 0 | 0 | 0 |
| RIENLSKKVNDGFLDIWTYNAELLVLLENERTLDYHDSNVKNLYEKVRSQLKNNA | 0 | 0 | 0 | 0 | 0 | 0 | 1 | 0 | 0 | 0 | 0 | 0 | 0 | 0 | 0 | 0 | 0 | 0 |
| RIENLSKKVDDGFLDIWTYNAELLILLENERTLDYHDSNVKNLYEKVRSQLKNNA | 0 | 0 | 0 | 0 | 0 | 0 | 1 | 0 | 0 | 0 | 0 | 0 | 0 | 0 | 0 | 0 | 0 | 0 |
| RIENLNQKVDDGFLDIWTYNAELLVLLENERTLDYHDSNVKNLYEKVRSQLKNNA | 0 | 0 | 2 | 0 | 0 | 0 | 0 | 0 | 0 | 0 | 0 | 0 | 0 | 0 | 0 | 0 | 0 | 0 |
| RIENLNKRVDDGFPDIWTYNAELLVLLENERTLDYHDSNVKNLYEKVRSQLKNNA | 0 | 0 | 0 | 0 | 0 | 0 | 0 | 1 | 0 | 0 | 0 | 0 | 0 | 0 | 0 | 0 | 0 | 0 |
| RIENLNKRVDDGFLDIWTYNAELLVLLENERTLDYHDSNVENLYEKVRSQLKNNA | 0 | 0 | 0 | 0 | 0 | 0 | 0 | 0 | 1 | 0 | 0 | 0 | 0 | 0 | 0 | 0 | 0 | 0 |
| RIENLNKRVDDGFLDIWTYNAELLVLLENERTLDYHDSKVKNLYEKVRSQLKNNA | 0 | 0 | 0 | 0 | 0 | 0 | 0 | 0 | 0 | 1 | 0 | 0 | 0 | 0 | 0 | 0 | 0 | 0 |
| RIENLNKRVDDGFLDIWTYNAELLILLENERTLDYHDSNVKNLYEKVRSQLKNNA | 0 | 0 | 0 | 0 | 0 | 0 | 0 | 0 | 0 | 0 | 0 | 0 | 0 | 0 | 1 | 0 | 0 | 0 |
| RIENLNKQVNDGFLDIWTYNAELLVLLENERTLDYHDSNVKNLYEKVRSQLKNNA | 0 | 0 | 0 | 0 | 0 | 0 | 0 | 0 | 1 | 0 | 0 | 0 | 0 | 0 | 0 | 0 | 0 | 0 |
| RIENLNKKVNNGFLDIWTYNAELLVLLENERTLDYHDSNVKNLYEKVRSQLKNNA | 0 | 0 | 0 | 0 | 0 | 0 | 0 | 0 | 0 | 0 | 0 | 0 | 0 | 0 | 1 | 0 | 0 | 0 |
| RIENLNKKVNDSFLDIWTYNAELLVLLENERTLDYHDSNVKNLYEKVRSQLKNNA | 0 | 0 | 0 | 0 | 0 | 0 | 1 | 0 | 0 | 0 | 0 | 0 | 0 | 0 | 0 | 0 | 0 | 0 |
| RIENLNKKVNDGFQDIWTYNAELLVLLENERTLDYHDSNVKNLYEKVRSQLKNNA | 0 | 0 | 0 | 0 | 0 | 0 | 1 | 0 | 0 | 0 | 0 | 0 | 0 | 0 | 0 | 0 | 0 | 0 |
| RIENLNKKVNDGFPDIWTYNAELLVLLENERTLDYHDSNVKNLYEKVRSQLKNNA | 0 | 0 | 0 | 0 | 0 | 1 | 0 | 0 | 0 | 0 | 0 | 0 | 0 | 0 | 0 | 0 | 0 | 0 |
| RIENLNKKVNDGFLEIWTYNAELLVLLENERTLDYHDSNVKNLYEKVRSQLKNNA | 0 | 0 | 0 | 0 | 0 | 0 | 0 | 1 | 0 | 0 | 0 | 0 | 0 | 0 | 0 | 0 | 0 | 0 |
| RIENLNKKVNDGFLDTWTYNAELLVLLENERTLDYHDSNVKNLYEKVRSQLKNNA | 0 | 0 | 0 | 0 | 1 | 0 | 0 | 0 | 0 | 0 | 0 | 0 | 0 | 0 | 0 | 0 | 0 | 0 |
| RIENLNKKVNDGFLDIWTYSAELLVLLENERTLDYHDSNVKNLYEKVRSQLKNNA | 0 | 0 | 0 | 0 | 0 | 0 | 1 | 0 | 0 | 0 | 0 | 0 | 0 | 0 | 0 | 0 | 0 | 0 |
| RIENLNKKVNDGFLDIWTYNVELLVLLENERTLDYHDSNVKNLYEKVRSQLKNNA | 0 | 0 | 0 | 0 | 0 | 0 | 0 | 0 | 1 | 0 | 0 | 0 | 0 | 0 | 0 | 0 | 0 | 0 |
| RIENLNKKVNDGFLDIWTYNTELLVLLENERTLDYHDSNVKNLYEKVRSQLKNNA | 0 | 0 | 0 | 0 | 0 | 0 | 0 | 1 | 0 | 0 | 0 | 0 | 0 | 0 | 0 | 0 | 0 | 0 |
| RIENLNKKVNDGFLDIWTYNDELLVLLENERTLDYHDSNVKNLYEKVRSQLKNNA | 0 | 0 | 0 | 0 | 0 | 1 | 0 | 0 | 0 | 0 | 0 | 0 | 0 | 0 | 0 | 0 | 0 | 0 |
| RIENLNKKVNDGFLDIWTYNAKLLVLLENERTLDYHDSNVKNLYEKVRSQLKNNA | 0 | 0 | 0 | 0 | 0 | 0 | 0 | 0 | 1 | 0 | 0 | 0 | 0 | 0 | 0 | 0 | 0 | 0 |
| RIENLNKKVNDGFLDIWTYNAELLVLSENERTLDYHDSNVKNLYEKVRSQLKNNA | 0 | 0 | 0 | 0 | 0 | 0 | 0 | 0 | 1 | 0 | 0 | 0 | 0 | 0 | 0 | 0 | 0 | 0 |
| RIENLNKKVNDGFLDIWTYNAELLVLLGNERTLDYHDSNVKNLYEKVRSQLKNNA | 0 | 0 | 0 | 0 | 0 | 0 | 0 | 0 | 3 | 0 | 0 | 0 | 0 | 0 | 0 | 0 | 0 | 0 |
| RIENLNKKVNDGFLDIWTYNAELLVLLESERTLDYHDSNVKNLYEKVRSQLKNNA | 0 | 0 | 0 | 0 | 0 | 1 | 0 | 0 | 0 | 0 | 0 | 0 | 0 | 0 | 0 | 0 | 0 | 0 |
| RIENLNKKVNDGFLDIWTYNAELLVLLENGRTLDYHDSNVKNLYEKVRSQLKNNA | 0 | 0 | 0 | 0 | 0 | 0 | 1 | 0 | 0 | 0 | 0 | 0 | 0 | 0 | 0 | 0 | 0 | 0 |
| RIENLNKKVNDGFLDIWTYNAELLVLLENERTSDYHDSNVKNLYEKVRSQLKNNA | 0 | 0 | 0 | 0 | 0 | 1 | 0 | 0 | 0 | 0 | 0 | 0 | 0 | 0 | 0 | 0 | 0 | 0 |
| RIENLNKKVNDGFLDIWTYNAELLVLLENERTLNYHDSNVKNLYEKVRSQLKNNA | 0 | 2 | 0 | 0 | 0 | 0 | 0 | 0 | 0 | 0 | 0 | 0 | 0 | 0 | 0 | 0 | 0 | 0 |
| RIENLNKKVNDGFLDIWTYNAELLVLLENERTLEYHDSNVKNLYEKVRSQLKNNA | 0 | 0 | 0 | 0 | 0 | 0 | 0 | 0 | 1 | 0 | 0 | 0 | 0 | 0 | 0 | 0 | 0 | 0 |
| RIENLNKKVNDGFLDIWTYNAELLVLLENERTLEYHDSNVKNLYEKVRNQLKNNA | 0 | 0 | 0 | 0 | 0 | 0 | 1 | 0 | 0 | 0 | 0 | 0 | 0 | 0 | 0 | 0 | 0 | 0 |
| RIENLNKKVNDGFLDIWTYNAELLVLLENERTLDYRDSNVKNLYEKVRSQLKNNA | 0 | 0 | 0 | 0 | 0 | 1 | 0 | 0 | 0 | 0 | 0 | 0 | 0 | 0 | 0 | 0 | 0 | 0 |
| RIENLNKKVNDGFLDIWTYNAELLVLLENERTLDYQDSNVKNLYEKVRSQLKNNA | 0 | 0 | 0 | 0 | 0 | 0 | 0 | 0 | 1 | 0 | 0 | 0 | 0 | 0 | 0 | 0 | 0 | 0 |
| RIENLNKKVNDGFLDIWTYNAELLVLLENERTLDYHYSNVKNLYEKVRSQLKNNA | 0 | 0 | 0 | 0 | 0 | 0 | 1 | 0 | 0 | 0 | 0 | 0 | 0 | 0 | 0 | 0 | 0 | 0 |
| RIENLNKKVNDGFLDIWTYNAELLVLLENERTLDYHDSSVKNLYEKVRSQLKNNA | 0 | 0 | 0 | 0 | 0 | 0 | 0 | 1 | 0 | 0 | 0 | 0 | 0 | 0 | 0 | 0 | 0 | 0 |
| RIENLNKKVNDGFLDIWTYNAELLVLLENERTLDYHDSNVKSLYEKVRSQLKNNA | 0 | 0 | 0 | 0 | 0 | 0 | 1 | 0 | 0 | 0 | 0 | 0 | 0 | 0 | 0 | 0 | 0 | 0 |
| RIENLNKKVNDGFLDIWTYNAELLVLLENERTLDYHDSNVKNSYEKVRSQLKNNA | 0 | 0 | 0 | 0 | 0 | 0 | 0 | 1 | 0 | 0 | 0 | 0 | 0 | 0 | 0 | 0 | 0 | 0 |
| RIENLNKKVNDGFLDIWTYNAELLVLLENERTLDYHDSNVKNLY?KVRSQLKNNA | 0 | 0 | 0 | 0 | 0 | 0 | 1 | 0 | 0 | 0 | 0 | 0 | 0 | 0 | 0 | 0 | 0 | 0 |
| RIENLNKKVNDGFLDIWTYNAELLVLLENERTLDYHDSNVKNLYETVRSQLKNNA | 0 | 0 | 0 | 0 | 1 | 0 | 0 | 0 | 0 | 0 | 0 | 0 | 0 | 0 | 0 | 0 | 0 | 0 |
| RIENLNKKVNDGFLDIWTYNAELLVLLENERTLDYHDSNVKNLYEKVRSRLKNNA | 0 | 0 | 0 | 0 | 0 | 1 | 0 | 0 | 0 | 0 | 0 | 0 | 0 | 0 | 0 | 0 | 0 | 0 |
| RIENLNKKVNDGFLDIWTYNAELLVLLENERTLDYHDSNVKNLYEKVRSQLKNSA | 0 | 0 | 0 | 0 | 0 | 0 | 0 | 0 | 1 | 0 | 0 | 0 | 0 | 0 | 0 | 0 | 0 | 0 |
| RIENLNKKVNDGFLDIWTYNAELLVLLENERTLDYHDSNVKNLYEKVRSQLKNNV | 0 | 0 | 0 | 0 | 0 | 0 | 0 | 0 | 1 | 0 | 0 | 0 | 0 | 0 | 0 | 0 | 0 | 0 |
| RIENLNKKVNDGFLDIWTYNAELLVLLENERTLDYHDSNVKNLYEKVRSQLKNHA | 0 | 0 | 0 | 0 | 0 | 0 | 0 | 0 | 1 | 0 | 0 | 0 | 0 | 0 | 0 | 0 | 0 | 0 |
| RIENLNKKVNDGFLDIWTYNAELLVLLENERTLDYHDSNVKNLYEKVRSQLKDNA | 0 | 0 | 0 | 0 | 0 | 0 | 0 | 0 | 2 | 0 | 0 | 0 | 0 | 0 | 0 | 0 | 0 | 0 |
| RIENLNKKVNDGFLDIWTYNAELLVLLENERTLDYHDSNVKNLYEKVRSQIKNNA | 0 | 0 | 0 | 0 | 0 | 0 | 0 | 0 | 1 | 0 | 0 | 0 | 0 | 0 | 0 | 0 | 0 | 0 |
| RIENLNKKVNDGFLDIWTYNAELLVLLENERTLDYHDSNVKNLYEKVRGQLKNNA | 0 | 1 | 0 | 0 | 0 | 0 | 0 | 0 | 0 | 0 | 0 | 0 | 0 | 0 | 0 | 0 | 0 | 0 |
| RIENLNKKVNDGFLDIWTYNAELLVLLENERTLDYHDSNVKNLYEKGRSQLKNNA | 0 | 0 | 0 | 1 | 0 | 0 | 0 | 0 | 0 | 0 | 0 | 0 | 0 | 0 | 0 | 0 | 0 | 0 |
| RIENLNKKVNDGFLDIWTYNAELLVLLENERTLDYHDSNVKNLYEKARSQLKNNA | 0 | 0 | 0 | 0 | 0 | 0 | 0 | 1 | 0 | 0 | 0 | 0 | 0 | 0 | 0 | 0 | 0 | 0 |

| | | | | | | | | | | | | | | | | | | |
|---|---|---|---|---|---|---|---|---|---|---|---|---|---|---|---|---|---|---|
| RIENLNKKVNDGFLDIWTYNAELLVLLENERTLDYHDSNVKNLHEKVRSQLKNNA | 0 | 0 | 0 | 0 | 0 | 0 | 0 | 0 | 1 | 0 | 0 | 0 | 0 | 0 | 0 | 0 | 0 | 0 |
| RIENLNKKVNDGFLDIWTYNAELLVLLENERTLDYHDSNVKNFYEKVRSQLKNNA | 0 | 0 | 0 | 0 | 1 | 0 | 0 | 0 | 0 | 0 | 0 | 0 | 0 | 0 | 0 | 0 | 0 | 0 |
| RIENLNKKVNDGFLDIWTYNAELLVLLENERTLDYHDSNVENLYEKVRSQLKNNA | 0 | 0 | 0 | 0 | 0 | 1 | 0 | 0 | 0 | 0 | 0 | 0 | 0 | 0 | 0 | 0 | 0 | 0 |
| RIENLNKKVNDGFLDIWTYNAELLVLLENERTLDYHDSDVKNLYEKVRSQLKNNA | 0 | 0 | 0 | 0 | 0 | 0 | 1 | 0 | 0 | 0 | 0 | 0 | 0 | 0 | 0 | 0 | 0 | 0 |
| RIENLNKKVNDGFLDIWTYNAELLVLLENERTLDYHDLNVKNLYEKVRSQLKNNA | 0 | 0 | 0 | 0 | 0 | 1 | 0 | 0 | 0 | 0 | 0 | 0 | 0 | 0 | 0 | 0 | 0 | 0 |
| RIENLNKKVNDGFLDIWTYNAELLVLLENERTLDSHDSNVKNLYEKVRSQLKNNA | 0 | 0 | 0 | 0 | 1 | 0 | 0 | 0 | 0 | 0 | 0 | 0 | 0 | 0 | 0 | 0 | 0 | 0 |
| RIENLNKKVNDGFLDIWTYNAELLVLLENERTLDCHDSNVKNLYEKVRSQLKNNA | 0 | 0 | 0 | 0 | 0 | 0 | 2 | 0 | 0 | 0 | 0 | 0 | 0 | 0 | 0 | 0 | 0 | 0 |
| RIENLNKKVNDGFLDIWTYNAELLVLLENERNLDYHDSNVKNLYEKVRSQLKNNA | 0 | 0 | 0 | 0 | 0 | 0 | 0 | 0 | 1 | 0 | 0 | 0 | 0 | 0 | 0 | 0 | 0 | 0 |
| RIENLNKKVNDGFLDIWTYNAELLVLLENEITLDYHDSNVKNLYEKVRSQLKNNA | 0 | 0 | 0 | 0 | 0 | 0 | 0 | 1 | 0 | 0 | 0 | 0 | 0 | 0 | 0 | 0 | 0 | 0 |
| RIENLNKKVNDGFLDIWTYNAELLVLLDNERTLDYHDSNVKNLYEKVRSQLKNNA | 0 | 0 | 0 | 0 | 0 | 0 | 0 | 1 | 0 | 0 | 0 | 0 | 0 | 0 | 0 | 0 | 0 | 0 |
| RIENLNKKVNDGFLDIWTYHAELLVLLENERTLDYHDSNVKNLYEKVRSQLKNNA | 0 | 0 | 0 | 0 | 0 | 0 | 0 | 1 | 0 | 0 | 0 | 0 | 0 | 0 | 0 | 0 | 0 | 0 |
| RIENLNKKVNDGFLDIWTHNAELLVLLENERTLDYHDSNVKNLYEKVRSQLKNNA | 0 | 0 | 0 | 0 | 0 | 0 | 0 | 1 | 0 | 0 | 0 | 0 | 0 | 0 | 0 | 0 | 0 | 0 |
| RIENLNKKVNDGFLDIWSYNAELLVLLENERTLDYHDSNVKNLYEKVRSQLKNNA | 0 | 0 | 0 | 0 | 0 | 0 | 0 | 1 | 0 | 0 | 0 | 0 | 0 | 0 | 0 | 0 | 0 | 0 |
| RIENLNKKVNDGFLDIWNYNAELLVLLENERTLDYHDSNVKNLYEKVRSQLKNNA | 0 | 0 | 0 | 0 | 0 | 0 | 0 | 1 | 0 | 0 | 0 | 0 | 0 | 0 | 0 | 0 | 0 | 0 |
| RIENLNKKVNDGFLDIWIYNAELLVLLENERTLDYHDSNVKNLYEKVRSQLKNNA | 0 | 0 | 0 | 0 | 0 | 0 | 0 | 1 | 0 | 0 | 0 | 0 | 0 | 0 | 0 | 0 | 0 | 0 |
| RIENLNKKVKDGFLDIWTYNAELLVLLENERTLDYHDSNVKNLYEKVRSQLKNNA | 0 | 0 | 1 | 0 | 0 | 0 | 0 | 0 | 0 | 0 | 0 | 0 | 0 | 0 | 0 | 0 | 0 | 0 |
| RIENLNKKVDYGFLDIWTYNAELLVLLENERTLDYHDSNVKNLYEKVRSQLKNNA | 0 | 0 | 0 | 0 | 0 | 0 | 0 | 0 | 0 | 1 | 0 | 0 | 0 | 0 | 0 | 0 | 0 | 0 |
| RIENLNKKVDVGFLDIWTYNAELLVLLENERTLDYHDSNVKNLYEKVRSQLKNNA | 0 | 0 | 0 | 0 | 0 | 0 | 0 | 0 | 0 | 0 | 0 | 0 | 0 | 1 | 0 | 0 | 0 | 0 |
| RIENLNKKVDGCFLDIWTYNAELLVLLENERTLDYHDSNVKNLYEKVRSQLKNNA | 0 | 0 | 0 | 0 | 0 | 0 | 0 | 1 | 0 | 0 | 0 | 0 | 0 | 0 | 0 | 0 | 0 | 0 |
| RIENLNKKVDDSFLDIWTYNAELLVLLGNERTLDYHDSNVKNLYEKVRSQLKNNA | 0 | 0 | 0 | 0 | 0 | 0 | 1 | 0 | 0 | 0 | 0 | 0 | 0 | 0 | 0 | 0 | 0 | 0 |
| RIENLNKKVDDGSLDIWTYNAELLVLLENERTLDYHDSSVKNLYEKVRSQLKNNA | 0 | 0 | 0 | 0 | 0 | 0 | 0 | 0 | 0 | 1 | 0 | 0 | 0 | 0 | 0 | 0 | 0 | 0 |
| RIENLNKKVDDGSLDIWTYNAELLVLLENERTLDYHDSNVKNLYEKARSQLKNNA | 0 | 0 | 0 | 0 | 1 | 0 | 0 | 0 | 0 | 0 | 0 | 0 | 0 | 0 | 0 | 0 | 0 | 0 |
| RIENLNKKVDDGLLDTRTYNAELLAPLENERTPDYHDSNVKNPYEKVRSQPKNNA | 0 | 0 | 0 | 0 | 0 | 0 | 0 | 0 | 0 | 0 | 0 | 0 | 0 | 1 | 0 | 0 | 0 | 0 |
| RIENLNKKVDDGILDIWTYNAELLVLLENERTLDYHDSNVKNLYEKVRSQLKNNA | 0 | 0 | 0 | 1 | 0 | 0 | 0 | 0 | 0 | 0 | 0 | 0 | 0 | 0 | 0 | 0 | 0 | 0 |
| RIENLNKKVDDGFRDIWTYNAELLVLLENERTLDYHDSNVKNLYEKVRSQLKNNA | 0 | 0 | 0 | 0 | 0 | 0 | 1 | 0 | 0 | 0 | 0 | 0 | 0 | 0 | 0 | 0 | 0 | 0 |
| RIENLNKKVDDGFPDIWTYNAEPLVPLENERTLDYHDSNVKNLYEKVRSQLKNNA | 0 | 0 | 0 | 0 | 0 | 0 | 0 | 0 | 0 | 1 | 0 | 0 | 0 | 0 | 0 | 0 | 0 | 0 |
| RIENLNKKVDDGFPDIWTHNAEPLVPLENERTLDYHDSNVKNLYEKVRSQLKNNA | 0 | 0 | 0 | 0 | 0 | 0 | 0 | 0 | 0 | 0 | 0 | 0 | 0 | 1 | 0 | 0 | 0 | 0 |
| RIENLNKKVDDGFLYIWTYNAELLVLLENERTLDYHDSNVKNLYEKVRSQLKNNA | 0 | 0 | 0 | 0 | 0 | 0 | 0 | 0 | 0 | 0 | 0 | 2 | 0 | 0 | 0 | 0 | 0 | 0 |
| RIENLNKKVDDGFLNIWTYNAELLVLLENERTLDYHDSNVKNLYEKVRNQLKNNA | 0 | 0 | 0 | 0 | 0 | 0 | 0 | 0 | 0 | 0 | 0 | 0 | 0 | 0 | 0 | 0 | 1 | 0 |
| RIENLNKKVDDGFLNIWPYNAELLVLLENERTLDYHDSNVKNLYEKVRSQLKNNA | 0 | 0 | 1 | 0 | 0 | 0 | 0 | 0 | 0 | 0 | 0 | 0 | 0 | 0 | 0 | 0 | 0 | 0 |
| RIENLNKKVDDGFLGIWTYNAELLVLLENERTLDYHDSNVKNLYEKVRNQLKNNA | 0 | 0 | 0 | 0 | 0 | 0 | 0 | 0 | 0 | 0 | 0 | 0 | 0 | 0 | 0 | 0 | 1 | 0 |
| RIENLNKKVDDGFLDVWTYNAELLVLLENERTLDYHDSNVKNLYEKVRNQLKNNA | 0 | 0 | 0 | 0 | 0 | 0 | 0 | 0 | 1 | 0 | 0 | 0 | 0 | 0 | 0 | 0 | 0 | 0 |
| RIENLNKKVDDGFLDTWTYNAELSVPLENERTLDHHDSNVKNLYEKVRSQLKNNA | 0 | 0 | 0 | 0 | 0 | 0 | 0 | 0 | 0 | 1 | 0 | 0 | 0 | 0 | 0 | 0 | 0 | 0 |
| RIENLNKKVDDGFLDTRTHNAELLVLLENERTLDYHDSNVKNLYEKVRSQLKNNA | 0 | 0 | 0 | 0 | 0 | 0 | 0 | 0 | 0 | 0 | 0 | 0 | 0 | 0 | 0 | 1 | 0 | 0 |
| RIENLNKKVDDGFLDTRTHNAELLAPLENERTLDYHDSNVKNLYEKVRSQLKNNA | 0 | 0 | 0 | 0 | 0 | 0 | 0 | 0 | 0 | 1 | 0 | 0 | 0 | 0 | 0 | 0 | 0 | 0 |
| RIENLNKKVDDGFLDMWTYNAELLVLLENERTLDYHDSNVKNLYEKVRSQLKNNA | 0 | 0 | 0 | 0 | 0 | 0 | 0 | 0 | 0 | 0 | 0 | 0 | 0 | 1 | 0 | 0 | 0 | 0 |
| RIENLNKKVDDGFLDIWTYNVKLLVLLENERTLDYHDSNVKNLYEKVRSQLKNNA | 0 | 0 | 0 | 0 | 0 | 0 | 0 | 1 | 0 | 0 | 0 | 0 | 0 | 0 | 0 | 0 | 0 | 0 |
| RIENLNKKVDDGFLDIWTYNVELSVLLENERTLDYHDSNVKNLYEKVRSQLKNNA | 0 | 0 | 0 | 0 | 1 | 0 | 0 | 0 | 0 | 0 | 0 | 0 | 0 | 0 | 0 | 0 | 0 | 0 |
| RIENLNKKVDDGFLDIWTYNVELLVLLKNERTLDYHDSNVKNLYEKVRSQLKNNA | 0 | 0 | 0 | 0 | 0 | 0 | 0 | 0 | 1 | 0 | 0 | 0 | 0 | 0 | 0 | 0 | 0 | 0 |
| RIENLNKKVDDGFLDIWTYNVELLVLLGNERTLDYHDSNVKNLYEKVRSQLKNNA | 0 | 0 | 0 | 0 | 0 | 0 | 0 | 1 | 0 | 0 | 0 | 0 | 0 | 0 | 0 | 0 | 0 | 0 |
| RIENLNKKVDDGFLDIWTYNVELLVLLENGRTLDYHDSNVKNLYEKVRSQLKNNA | 0 | 0 | 0 | 0 | 1 | 0 | 0 | 0 | 0 | 0 | 0 | 0 | 0 | 0 | 0 | 0 | 0 | 0 |
| RIENLNKKVDDGFLDIWTYNVELLVLLENERTLDYNDSNVKNLYEKVRSQLKNNA | 0 | 0 | 0 | 0 | 0 | 0 | 0 | 0 | 1 | 0 | 0 | 0 | 0 | 0 | 0 | 0 | 0 | 0 |
| RIENLNKKVDDGFLDIWTYNVELLVLLENERTLDYHDTNVKNLYEKVRSQLKNNA | 0 | 0 | 0 | 0 | 0 | 0 | 0 | 0 | 1 | 0 | 0 | 0 | 0 | 0 | 0 | 0 | 0 | 0 |
| RIENLNKKVDDGFLDIWTYNVELLVLLENERTLDYHDSNVKSLYEKVRSQLKNNA | 0 | 0 | 0 | 0 | 1 | 0 | 0 | 0 | 0 | 0 | 0 | 0 | 0 | 0 | 0 | 0 | 0 | 0 |
| RIENLNKKVDDGFLDIWTYNVELLVLLENERTLDYHDSNVKNLYVKVRSQLKNNA | 0 | 0 | 0 | 0 | 0 | 0 | 0 | 0 | 1 | 0 | 0 | 0 | 0 | 0 | 0 | 0 | 0 | 0 |
| RIENLNKKVDDGFLDIWTYNVELLVLLENERTLDYHDSNVKNLYEKVRSQLKNNG | 0 | 0 | 0 | 0 | 0 | 0 | 0 | 0 | 1 | 0 | 0 | 0 | 0 | 0 | 0 | 0 | 0 | 0 |
| RIENLNKKVDDGFLDIWTYNVELLVLLENERTLDYHDSNVKNLYEKVRRQLKNNA | 0 | 0 | 0 | 0 | 0 | 0 | 1 | 0 | 0 | 0 | 0 | 0 | 0 | 0 | 0 | 0 | 0 | 0 |
| RIENLNKKVDDGFLDIWTYNVELLVLLENERTLDYHDSNVKKLYEKVRSQLKNNA | 0 | 0 | 0 | 0 | 0 | 0 | 0 | 0 | 0 | 0 | 0 | 0 | 1 | 0 | 0 | 0 | 0 | 0 |
| RIENLNKKVDDGFLDIWTYNVELLVLLENERTLDYHASNVKNLYEKVRSQLKNNA | 0 | 0 | 0 | 0 | 0 | 0 | 0 | 0 | 1 | 0 | 0 | 0 | 0 | 0 | 0 | 0 | 0 | 0 |
| RIENLNKKVDDGFLDIWTYNVELLVLLENEETLDYHDSNVKNLYEKVRSQLKNNA | 0 | 0 | 0 | 0 | 0 | 0 | 0 | 0 | 1 | 0 | 0 | 0 | 0 | 0 | 0 | 0 | 0 | 0 |
| RIENLNKKVDDGFLDIWTYNVELLVLLEKERTLDYHDSNVKNLYEKVRSQLKNNA | 0 | 0 | 0 | 0 | 0 | 0 | 1 | 0 | 0 | 0 | 0 | 0 | 0 | 0 | 0 | 0 | 0 | 0 |
| RIENLNKKVDDGFLDIWTYNTELLVLLENERTLDYHDSNVRNLYEKVRSQLKNNA | 0 | 0 | 0 | 0 | 0 | 0 | 0 | 0 | 0 | 0 | 0 | 0 | 0 | 0 | 0 | 0 | 1 | 0 |
| RIENLNKKVDDGFLDIWTYNTELLVLLENERTLDYHDSNVKNLYEKVRSQLKHNA | 0 | 0 | 0 | 0 | 0 | 0 | 0 | 0 | 1 | 0 | 0 | 0 | 0 | 0 | 0 | 0 | 0 | 0 |
| RIENLNKKVDDGFLDIWTYNTELLVLLENERTLDYHDSNVKNLYEKVRRQLKNNA | 0 | 0 | 0 | 0 | 0 | 0 | 0 | 0 | 1 | 0 | 0 | 0 | 0 | 0 | 0 | 0 | 0 | 0 |
| RIENLNKKVDDGFLDIWTYNTELLVLLENERTLDYHDSNVKNLYEKVGSQLKNNA | 0 | 0 | 0 | 0 | 0 | 0 | 0 | 0 | 1 | 0 | 0 | 0 | 0 | 0 | 0 | 0 | 0 | 0 |
| RIENLNKKVDDGFLDIWTYNTELLVLLENERTLDYHDSNMKNLYEKVRSQLKNNA | 0 | 0 | 0 | 0 | 0 | 0 | 1 | 0 | 0 | 0 | 0 | 0 | 0 | 0 | 0 | 0 | 0 | 0 |
| RIENLNKKVDDGFLDIWTYNTELLVLLENERTLDYHDSNAKNLYEKVRSQLKNNA | 0 | 0 | 0 | 0 | 0 | 0 | 1 | 0 | 0 | 0 | 0 | 0 | 0 | 0 | 0 | 0 | 0 | 0 |
| RIENLNKKVDDGFLDIWTYNSELLVLLENERTLDYHDSNVKNLYEKVRSQLKNNA | 0 | 0 | 0 | 0 | 0 | 0 | 0 | 0 | 0 | 0 | 1 | 0 | 0 | 0 | 0 | 0 | 0 | 0 |
| RIENLNKKVDDGFLDIWTYNIELLVLLENERTLDYHDSNVKNLYEKVRSQLKNNA | 0 | 0 | 0 | 0 | 0 | 0 | 1 | 0 | 0 | 0 | 0 | 0 | 0 | 0 | 0 | 0 | 0 | 0 |
| RIENLNKKVDDGFLDIWTYNGELLVLLENERTLDYHDSNVKSLYEKVRSQLKNNA | 0 | 0 | 0 | 0 | 0 | 0 | 0 | 0 | 1 | 0 | 0 | 0 | 0 | 0 | 0 | 0 | 0 | 0 |
| RIENLNKKVDDGFLDIWTYNAVLLVLLENERTLDYHDSNVKNLYEKVRSQLKNNA | 0 | 0 | 0 | 0 | 0 | 0 | 0 | 0 | 2 | 0 | 0 | 0 | 0 | 0 | 0 | 0 | 0 | 0 |
| RIENLNKKVDDGFLDIWTYNAETLVLLENERTLDYHDSNVKNLYEKVRSQLKNNA | 0 | 0 | 0 | 0 | 0 | 0 | 2 | 0 | 0 | 0 | 0 | 0 | 0 | 0 | 0 | 0 | 0 | 0 |
| RIENLNKKVDDGFLDIWTYNAEPLVLLENERTLDYHDSNVKNLYEKVRNQLKNNA | 0 | 0 | 0 | 0 | 0 | 0 | 0 | 0 | 0 | 0 | 0 | 0 | 0 | 0 | 1 | 0 | 0 | 0 |
| RIENLNKKVDDGFLDIWTYNAEPLVLLENERTLDYHDSNVKNLYEKARSQLKNNA | 0 | 0 | 0 | 0 | 0 | 0 | 0 | 0 | 0 | 0 | 0 | 0 | 0 | 0 | 0 | 0 | 0 | 1 |
| RIENLNKKVDDGFLDIWTYNAEMLVLLENERTLDYHDSNVKNLYERVRSQLKNNA | 0 | 0 | 0 | 0 | 1 | 0 | 0 | 0 | 0 | 0 | 0 | 0 | 0 | 0 | 0 | 0 | 0 | 0 |
| RIENLNKKVDDGFLDIWTYNAEMLILLENERTLDYHDSNVKNLYEKVRSQLKNNA | 0 | 0 | 0 | 0 | 0 | 0 | 0 | 0 | 0 | 0 | 0 | 0 | 0 | 1 | 0 | 0 | 0 | 0 |
| RIENLNKKVDDGFLDIWTYNAELWVLLENERTLDYHDSNVKNLYEKVRSQLKNNA | 0 | 0 | 0 | 0 | 0 | 0 | 0 | 0 | 1 | 0 | 0 | 0 | 0 | 0 | 0 | 0 | 0 | 0 |
| RIENLNKKVDDGFLDIWTYNAELVVLLENERTLDYHDSNVKNLYEKVRNQLKNNA | 0 | 0 | 0 | 0 | 0 | 0 | 1 | 0 | 0 | 0 | 0 | 0 | 0 | 0 | 0 | 0 | 0 | 0 |
| RIENLNKKVDDGFLDIWTYNAELSVLLENERTLDYHDSNVKNLHEKVRSQLKNNA | 1 | 0 | 0 | 0 | 0 | 0 | 0 | 0 | 0 | 0 | 0 | 0 | 0 | 0 | 0 | 0 | 0 | 0 |
| RIENLNKKVDDGFLDIWTYNAELLVVLENERTLDYHDSNVKNLYEKVRSQLKNNA | 0 | 0 | 0 | 0 | 0 | 0 | 0 | 1 | 0 | 0 | 0 | 0 | 0 | 0 | 0 | 0 | 0 | 0 |
| RIENLNKKVDDGFLDIWTYNAELLVRLENERTLDYHDSNVKNLYEKVRSQLKNNA | 0 | 0 | 0 | 0 | 0 | 0 | 0 | 0 | 0 | 0 | 0 | 0 | 1 | 0 | 0 | 0 | 0 | 0 |
| RIENLNKKVDDGFLDIWTYNAELLVPLENERTLDHHDSNVKNSYEKARSQLKNNA | 0 | 0 | 0 | 0 | 0 | 0 | 0 | 0 | 0 | 1 | 0 | 0 | 0 | 0 | 0 | 0 | 0 | 0 |
| RIENLNKKVDDGFLDIWTYNAELLVLSENERTLDYHDSNVKNLYEKVRSQLKNDA | 0 | 0 | 0 | 0 | 0 | 0 | 2 | 0 | 0 | 0 | 0 | 0 | 0 | 0 | 0 | 0 | 0 | 0 |
| RIENLNKKVDDGFLDIWTYNAELLVLLQNERTLDYHDSNVKNLYEKVRSQLKNNA | 0 | 0 | 0 | 0 | 0 | 0 | 0 | 0 | 0 | 0 | 0 | 0 | 0 | 0 | 1 | 0 | 0 | 0 |
| RIENLNKKVDDGFLDIWTYNAELLVLLKNERTLHYHDSNVKNLYEKVRSQLKNNA | 0 | 0 | 0 | 0 | 0 | 0 | 0 | 0 | 0 | 1 | 0 | 0 | 0 | 0 | 0 | 0 | 0 | 0 |
| RIENLNKKVDDGFLDIWTYNAELLVLLGNERTLDYHDSNVKSLYEKVRSQLKNNA | 0 | 0 | 0 | 0 | 0 | 0 | 0 | 1 | 0 | 0 | 0 | 0 | 0 | 0 | 0 | 0 | 0 | 0 |
| RIENLNKKVDDGFLDIWTYNAELLVLLETERTLDYHDSNVKNLYEKVRSQLKNNA | 0 | 0 | 0 | 0 | 0 | 0 | 0 | 1 | 0 | 0 | 0 | 0 | 0 | 0 | 0 | 0 | 0 | 0 |
| RIENLNKKVDDGFLDIWTYNAELLVLLESERTLDYHDSNVKNLYERVRSQLKNNA | 0 | 0 | 0 | 0 | 0 | 0 | 0 | 0 | 0 | 0 | 0 | 1 | 0 | 0 | 0 | 0 | 0 | 0 |
| RIENLNKKVDDGFLDIWTYNAELLVLLENVRTLDYHDSNVKNLYEKVRSQLKNNA | 0 | 0 | 0 | 0 | 0 | 0 | 0 | 1 | 0 | 0 | 0 | 0 | 0 | 0 | 0 | 0 | 0 | 0 |
| RIENLNKKVDDGFLDIWTYNAELLVLLENQRTLDYHDSNVKNLYEKVRSQLKNNA | 0 | 0 | 0 | 0 | 0 | 0 | 1 | 0 | 0 | 0 | 0 | 0 | 0 | 0 | 0 | 0 | 0 | 0 |
| RIENLNKKVDDGFLDIWTYNAELLVLLENESTLDYHDSNVKNLYEKVRSQLKNNA | 0 | 0 | 0 | 0 | 0 | 0 | 0 | 0 | 1 | 0 | 0 | 0 | 0 | 0 | 0 | 0 | 0 | 0 |
| RIENLNKKVDDGFLDIWTYNAELLVLLENESTLDYHDLNVKNLYEKVRSQLKNNA | 0 | 0 | 0 | 0 | 0 | 0 | 0 | 0 | 0 | 0 | 0 | 0 | 0 | 0 | 1 | 0 | 0 | 0 |
| RIENLNKKVDDGFLDIWTYNAELLVLLENERTWNYHDSNVKNLYEKVRSQLKNNA | 0 | 0 | 0 | 0 | 0 | 0 | 0 | 0 | 1 | 0 | 0 | 0 | 0 | 0 | 0 | 0 | 0 | 0 |
| RIENLNKKVDDGFLDIWTYNAELLVLLENERTLYYHDSNVKNLYEKVRSQLKNNA | 0 | 0 | 0 | 0 | 0 | 0 | 0 | 1 | 0 | 0 | 0 | 0 | 0 | 0 | 0 | 0 | 0 | 0 |
| RIENLNKKVDDGFLDIWTYNAELLVLLENERTLHYHDSNVKNLYEKVRSQLKNNA | 0 | 0 | 0 | 0 | 0 | 0 | 0 | 0 | 1 | 0 | 0 | 0 | 0 | 0 | 0 | 0 | 0 | 0 |
| RIENLNKKVDDGFLDIWTYNAELLVLLENERTLGHHDSNVKNLYEKVRSQLKNNA | 0 | 0 | 0 | 0 | 0 | 0 | 0 | 0 | 1 | 0 | 0 | 0 | 0 | 0 | 0 | 0 | 0 | 0 |
| RIENLNKKVDDGFLDIWTYNAELLVLLENERTLEHHDSNVKNLYEKVRSQLKNNA | 0 | 0 | 0 | 0 | 0 | 0 | 0 | 1 | 0 | 0 | 0 | 0 | 0 | 0 | 0 | 0 | 0 | 0 |
| RIENLNKKVDDGFLDIWTYNAELLVLLENERTLDYYDSNVKNLYEKVRSQLKDNA | 0 | 0 | 0 | 0 | 0 | 0 | 1 | 0 | 0 | 0 | 0 | 0 | 0 | 0 | 0 | 0 | 0 | 0 |
| RIENLNKKVDDGFLDIWTYNAELLVLLENERTLDYHYSNMKNLYEKVRSQLKNNA | 0 | 0 | 0 | 0 | 0 | 1 | 0 | 0 | 0 | 0 | 0 | 0 | 0 | 0 | 0 | 0 | 0 | 0 |
| RIENLNKKVDDGFLDIWTYNAELLVLLENERTLDYHVSNVKNLYEKVRSQLKNNA | 0 | 0 | 0 | 0 | 0 | 0 | 0 | 0 | 1 | 0 | 0 | 0 | 0 | 0 | 0 | 0 | 0 | 0 |
| RIENLNKKVDDGFLDIWTYNAELLVLLENERTLDYH?SNVKNLYEKVRSQLKNNA | 0 | 0 | 0 | 0 | 0 | 0 | 0 | 0 | 1 | 0 | 0 | 0 | 0 | 0 | 0 | 0 | 0 | 0 |
| RIENLNKKVDDGFLDIWTYNAELLVLLENERTLDYHNSNVKNLYERVRSQLKNNA | 0 | 0 | 0 | 0 | 0 | 0 | 0 | 0 | 0 | 1 | 0 | 0 | 0 | 0 | 0 | 0 | 0 | 0 |
| RIENLNKKVDDGFLDIWTYNAELLVLLENERTLDYHNSNVKNLYEKVRSQLKNTA | 0 | 0 | 0 | 0 | 0 | 0 | 0 | 0 | 0 | 0 | 0 | 0 | 0 | 0 | 0 | 0 | 0 | 1 |
| RIENLNKKVDDGFLDIWTYNAELLVLLENERTLDYHDSNVKSLYEKVRNQLKNNA | 0 | 0 | 0 | 0 | 0 | 0 | 0 | 0 | 1 | 0 | 0 | 0 | 0 | 0 | 0 | 0 | 0 | 0 |
| RIENLNKKVDDGFLDIWTYNAELLVLLENERTLDYHDSNVKNLYVKVRSQLKNNA | 0 | 0 | 0 | 0 | 0 | 0 | 0 | 0 | 0 | 0 | 1 | 0 | 0 | 0 | 0 | 0 | 0 | 0 |
| RIENLNKKVDDGFLDIWTYNAELLVLLENERTLDYHDSNVKNLYGRVRSQLKNNA | 0 | 0 | 0 | 0 | 1 | 0 | 0 | 0 | 0 | 0 | 0 | 0 | 0 | 0 | 0 | 0 | 0 | 0 |
| RIENLNKKVDDGFLDIWTYNAELLVLLENERTLDYHDSNVKNLYEMVRSQLKNNA | 0 | 0 | 0 | 0 | 0 | 0 | 0 | 1 | 0 | 0 | 0 | 0 | 0 | 0 | 0 | 0 | 0 | 0 |
| RIENLNKKVDDGFLDIWTYNAELLVLLENERTLDYHDSNVKNLYEKVTSQLKNNA | 0 | 0 | 0 | 1 | 0 | 0 | 0 | 0 | 0 | 0 | 0 | 0 | 0 | 0 | 0 | 0 | 0 | 0 |
| RIENLNKKVDDGFLDIWTYNAELLVLLENERTLDYHDSNVKNLYEKVRTQLKNNA | 0 | 0 | 1 | 0 | 0 | 0 | 0 | 0 | 0 | 0 | 0 | 0 | 0 | 0 | 0 | 0 | 0 | 0 |
| RIENLNKKVDDGFLDIWTYNAELLVLLENERTLDYHDSNVKNLYEKVRSQLKNYA | 0 | 0 | 0 | 0 | 0 | 0 | 0 | 0 | 1 | 0 | 0 | 0 | 0 | 0 | 0 | 0 | 0 | 0 |
| RIENLNKKVDDGFLDIWTYNAELLVLLENERTLDYHDSNVKNLYEKVRSQLKNNG | 0 | 0 | 0 | 0 | 0 | 0 | 0 | 0 | 1 | 0 | 0 | 0 | 0 | 0 | 0 | 0 | 0 | 0 |
| RIENLNKKVDDGFLDIWTYNAELLVLLENERTLDYHDSNVKNLYEKVRSQLK?NA | 0 | 0 | 0 | 0 | 0 | 0 | 0 | 0 | 1 | 0 | 0 | 0 | 0 | 0 | 0 | 0 | 0 | 0 |
| RIENLNKKVDDGFLDIWTYNAELLVLLENERTLDYHDSNVKNLYEKVRNQLKNSA | 0 | 0 | 1 | 0 | 0 | 0 | 0 | 0 | 0 | 0 | 0 | 0 | 0 | 0 | 0 | 0 | 0 | 0 |
| RIENLNKKVDDGFLDIWTYNAELLVLLENERTLDYHDSNVKNLYEKERSQLKNNA | 0 | 0 | 0 | 0 | 0 | 0 | 0 | 0 | 0 | 0 | 0 | 0 | 0 | 1 | 0 | 0 | 0 | 0 |
| RIENLNKKVDDGFLDIWTYNAELLVLLENERTLDYHDSNVKNLYEKARSQSKNNA | 0 | 0 | 0 | 0 | 0 | 0 | 0 | 0 | 0 | 0 | 1 | 0 | 0 | 0 | 0 | 0 | 0 | 0 |
| RIENLNKKVDDGFLDIWTYNAELLVLLENERTLDYHDSNVKNLYDRVRSQLKNNA | 0 | 0 | 0 | 0 | 0 | 0 | 0 | 0 | 1 | 0 | 0 | 0 | 0 | 0 | 0 | 0 | 0 | 0 |
| RIENLNKKVDDGFLDIWTYNAELLVLLENERTLDYHDSNVKNLYDKVRNQLKNNA | 0 | 0 | 0 | 0 | 0 | 0 | 0 | 1 | 0 | 0 | 0 | 0 | 0 | 0 | 0 | 0 | 0 | 0 |
| RIENLNKKVDDGFLDIWTYNAELLVLLENERTLDYHDSNVKNLYAKVRSQLKNNA | 0 | 0 | 0 | 0 | 0 | 0 | 0 | 0 | 0 | 0 | 0 | 0 | 0 | 0 | 1 | 0 | 0 | 0 |
| RIENLNKKVDDGFLDIWTYNAELLVLLENERTLDYHDSNVKNLHEKVRSQSKNNA | 0 | 0 | 0 | 0 | 0 | 0 | 0 | 0 | 0 | 0 | 0 | 0 | 0 | 0 | 0 | 0 | 1 | 0 |
| RIENLNKKVDDGFLDIWTYNAELLVLLENERTLDYHDSNVKNLFEKVRSQLKNNA | 0 | 0 | 0 | 0 | 0 | 0 | 0 | 0 | 0 | 1 | 0 | 0 | 0 | 0 | 0 | 0 | 0 | 0 |
| RIENLNKKVDDGFLDIWTYNAELLVLLENERTLDYHDSNVKNLDEKVRSQLKNNA | 0 | 0 | 0 | 0 | 0 | 0 | 0 | 0 | 0 | 1 | 0 | 0 | 0 | 0 | 0 | 0 | 0 | 0 |
| RIENLNKKVDDGFLDIWTYNAELLVLLENERTLDYHDSNVKNLCEKVRNQLKNNA | 0 | 0 | 0 | 0 | 0 | 0 | 0 | 1 | 0 | 0 | 0 | 0 | 0 | 0 | 0 | 0 | 0 | 0 |
| RIENLNKKVDDGFLDIWTYNAELLVLLENERTLDYHDSNVKKIYEKVRSQLKNNA | 0 | 0 | 0 | 0 | 0 | 0 | 1 | 0 | 0 | 0 | 0 | 0 | 0 | 0 | 0 | 0 | 0 | 0 |
| RIENLNKKVDDGFLDIWTYNAELLVLLENERTLDYHDSNVENLYERVRSQLKNNA | 0 | 0 | 0 | 0 | 0 | 0 | 1 | 0 | 0 | 0 | 0 | 0 | 0 | 0 | 0 | 0 | 0 | 0 |
| RIENLNKKVDDGFLDIWTYNAELLVLLENERTLDYHDSNEKNLYEKVRSQLKNNA | 0 | 0 | 0 | 0 | 0 | 0 | 0 | 0 | 1 | 0 | 0 | 0 | 0 | 0 | 0 | 0 | 0 | 0 |
| RIENLNKKVDDGFLDIWTYNAELLVLLENERTLDYHDSNAKNLYEKVRSQSKSNA | 0 | 0 | 0 | 0 | 0 | 0 | 0 | 0 | 0 | 0 | 0 | 1 | 0 | 0 | 0 | 0 | 0 | 0 |
| RIENLNKKVDDGFLDIWTYNAELLVLLENERTLDYHDSNAKNLYEKVRSQLKDNA | 0 | 0 | 1 | 0 | 0 | 0 | 0 | 0 | 0 | 0 | 0 | 0 | 0 | 0 | 0 | 0 | 0 | 0 |

| | | | | | | | | | | | | | | | | | | |
|---|---|---|---|---|---|---|---|---|---|---|---|---|---|---|---|---|---|---|
| RIENLNKKVDDGFLDIWTYNAELLVLLENERTLDYHDSIVKNLYEKVRSQLKNNA | 0 | 0 | 0 | 0 | 0 | 0 | 0 | 0 | 0 | 0 | 1 | 0 | 0 | 0 | 0 | 0 | 0 | 0 |
| RIENLNKKVDDGFLDIWTYNAELLVLLENERTLDYHDSDVKNLYERVRSQLKNNA | 0 | 0 | 0 | 0 | 0 | 0 | 0 | 0 | 1 | 0 | 0 | 0 | 0 | 0 | 0 | 0 | 0 | 0 |
| RIENLNKKVDDGFLDIWTYNAELLVLLENERTLDYHDSCVKNLYEKVRSQLKNNA | 0 | 0 | 0 | 0 | 0 | 0 | 0 | 0 | 0 | 0 | 0 | 1 | 0 | 0 | 0 | 0 | 0 | 0 |
| RIENLNKKVDDGFLDIWTYNAELLVLLENERTLDYHDLNVKSLYEKVRSQLKNNA | 0 | 0 | 0 | 0 | 0 | 0 | 1 | 0 | 0 | 0 | 0 | 0 | 0 | 0 | 0 | 0 | 0 | 0 |
| RIENLNKKVDDGFLDIWTYNAELLVLLENERTLDYHASNVKNLYEKVRSQLKNNA | 0 | 0 | 0 | 0 | 0 | 0 | 0 | 0 | 0 | 0 | 0 | 0 | 1 | 0 | 0 | 0 | 0 | 0 |
| RIENLNKKVDDGFLDIWTYNAELLVLLENERTLDYDDSNVKNLYERVRSQLKNNA | 0 | 0 | 0 | 0 | 0 | 0 | 0 | 1 | 0 | 0 | 0 | 0 | 0 | 0 | 0 | 0 | 0 | 0 |
| RIENLNKKVDDGFLDIWTYNAELLVLLENERTLDYDDSNVKNLYEKVRSQLKNKA | 0 | 0 | 1 | 0 | 0 | 0 | 0 | 0 | 0 | 0 | 0 | 0 | 0 | 0 | 0 | 0 | 0 | 0 |
| RIENLNKKVDDGFLDIWTYNAELLVLLENERTLDHHDSNVKNSHEKVRSQLKNNA | 0 | 0 | 0 | 0 | 0 | 0 | 0 | 0 | 0 | 0 | 0 | 0 | 0 | 0 | 0 | 0 | 0 | 1 |
| RIENLNKKVDDGFLDIWTYNAELLVLLENERTLDCHDSNVKNLYEKVRSQLTNNA | 0 | 0 | 0 | 0 | 0 | 0 | 0 | 0 | 0 | 0 | 0 | 0 | 0 | 1 | 0 | 0 | 0 | 0 |
| RIENLNKKVDDGFLDIWTYNAELLVLLENERTFGLPRFKCENLYEKVRSQLKNNA | 0 | 0 | 0 | 0 | 0 | 0 | 0 | 1 | 0 | 0 | 0 | 0 | 0 | 0 | 0 | 0 | 0 | 0 |
| RIENLNKKVDDGFLDIWTYNAELLVLLENEKTLDYHNSNVKNLYEKVRSQLKNNA | 0 | 0 | 0 | 0 | 1 | 0 | 0 | 0 | 0 | 0 | 0 | 0 | 0 | 0 | 0 | 0 | 0 | 0 |
| RIENLNKKVDDGFLDIWTYNAELLVLLENEKTLDYHDSNVKNLYERVRSQLKNNA | 1 | 0 | 0 | 0 | 0 | 0 | 0 | 0 | 0 | 0 | 0 | 0 | 0 | 0 | 0 | 0 | 0 | 0 |
| RIENLNKKVDDGFLDIWTYNAELLVLLENEKRTLDYHDSNVKNLYEKVRSQLKNNA | 0 | 0 | 0 | 0 | 0 | 0 | 0 | 0 | 1 | 0 | 0 | 0 | 0 | 0 | 0 | 0 | 0 | 0 |
| RIENLNKKVDDGFLDIWTYNAELLVLLENEITLDYHDSNVKNLYEKVRSQLKNNA | 0 | 0 | 0 | 0 | 0 | 0 | 0 | 0 | 0 | 0 | 0 | 0 | 0 | 1 | 0 | 0 | 0 | 0 |
| RIENLNKKVDDGFLDIWTYNAELLVL?ENERTLDYHDSNVKNLYEKVRSQLKNNA | 0 | 0 | 0 | 0 | 0 | 0 | 0 | 0 | 1 | 0 | 0 | 0 | 0 | 0 | 0 | 0 | 0 | 0 |
| RIENLNKKVDDGFLDIWTYNAELLLLLENERTLDYHDSNVKNLYEKVRSQLKNNA | 0 | 0 | 0 | 0 | 0 | 0 | 1 | 0 | 0 | 0 | 0 | 0 | 0 | 0 | 0 | 0 | 0 | 0 |
| RIENLNKKVDDGFLDIWTYNAELLILLENERTLDYYDSNVKNLYEKVRSQLKNNA | 0 | 0 | 0 | 0 | 0 | 0 | 0 | 0 | 0 | 0 | 0 | 1 | 0 | 0 | 0 | 0 | 0 | 0 |
| RIENLNKKVDDGFLDIWTYNAELLILLENERTLDYHDSNVKNLYERVRSQLKNNA | 0 | 0 | 0 | 0 | 0 | 0 | 0 | 0 | 0 | 0 | 0 | 0 | 1 | 0 | 0 | 0 | 0 | 0 |
| RIENLNKKVDDGFLDIWTYNAELLILLENERTLDYHDSNVKNLYEKVRNQLKNNA | 1 | 0 | 0 | 0 | 0 | 0 | 0 | 0 | 0 | 0 | 0 | 0 | 0 | 0 | 0 | 0 | 0 | 0 |
| RIENLNKKVDDGFLDIWTYNAELLILLENERTLDYHDSNVENLYEKVRSQLKNNA | 0 | 0 | 0 | 0 | 0 | 0 | 0 | 0 | 0 | 1 | 0 | 0 | 0 | 0 | 0 | 0 | 0 | 0 |
| RIENLNKKVDDGFLDIWTYNAELLGLLENERTLDYHDSNVKNLYEKVRSQLKNNA | 0 | 0 | 0 | 0 | 1 | 0 | 0 | 0 | 0 | 0 | 0 | 0 | 0 | 0 | 0 | 0 | 0 | 0 |
| RIENLNKKVDDGFLDIWTYNAELLAPLENERTLDYHDSNVKNLYEKVRSQLKNNA | 0 | 0 | 0 | 0 | 1 | 0 | 0 | 0 | 0 | 0 | 0 | 0 | 0 | 0 | 0 | 0 | 0 | 0 |
| RIENLNKKVDDGFLDIWTYNAELFVLLENQRTLDYHDSNVKNLYEKVRSQLKNNA | 0 | 0 | 1 | 0 | 0 | 0 | 0 | 0 | 0 | 0 | 0 | 0 | 0 | 0 | 0 | 0 | 0 | 0 |
| RIENLNKKVDDGFLDIWTHNAELLVPLENERTLDYHDSNVKNLYEKVRSQLKNNA | 0 | 0 | 0 | 0 | 0 | 0 | 0 | 0 | 0 | 0 | 0 | 0 | 1 | 0 | 0 | 0 | 0 | 0 |
| RIENLNKKVDDGFLDIWTHNAELLVPLENERTLDYHDSNVKNLYEKARSQLKNNA | 0 | 0 | 0 | 0 | 0 | 0 | 0 | 0 | 0 | 0 | 0 | 1 | 0 | 0 | 0 | 0 | 0 | 0 |
| RIENLNKKVDDGFLDIRTYNAELLVPLENERTLDHHDPNVKNLYEKVRSQLKNNA | 0 | 0 | 0 | 0 | 1 | 0 | 0 | 0 | 0 | 0 | 0 | 0 | 0 | 0 | 0 | 0 | 0 | 0 |
| RIENLNKKVDDGFLDILTYNAELLVLLENGRTLDYHDSNVKNLYEKVRSQLKNNA | 0 | 0 | 0 | 0 | 0 | 0 | 0 | 0 | 0 | 0 | 1 | 0 | 0 | 0 | 0 | 0 | 0 | 0 |
| RIENLNKKVDDGFLDILTYNAELLILLENERTLDYHDSNVKNLYEKVRSQLKNNA | 0 | 0 | 0 | 0 | 0 | 0 | 0 | 0 | 1 | 0 | 0 | 0 | 0 | 0 | 0 | 0 | 0 | 0 |
| RIENLNKKVDDCFLDIWTYNAELLVLLENERTLDYHDSNVKNLYEKVRSQLKNNA | 0 | 0 | 0 | 0 | 0 | 0 | 0 | 1 | 0 | 0 | 0 | 0 | 0 | 0 | 0 | 0 | 0 | 0 |
| RIENLNKKVADGFLDIWTYNAELLVLLENERTLDYHDSNVKNLYEKVRSQLKNNA | 0 | 0 | 0 | 0 | 0 | 0 | 0 | 0 | 5 | 0 | 0 | 0 | 0 | 0 | 0 | 0 | 0 | 0 |
| RIENLNKKLDDGFLDIWTYNAELLVLLENERTLDYHDSNVKNLYEKVRSQLKNNA | 0 | 0 | 0 | 0 | 1 | 0 | 0 | 0 | 0 | 0 | 0 | 0 | 0 | 0 | 0 | 0 | 0 | 0 |
| RIENLNKKANDGFLDTWTYNAELLVLLENERTLDYHDSNVKNLYEKVRSQLKNNA | 0 | 0 | 0 | 0 | 0 | 0 | 0 | 0 | 0 | 1 | 0 | 0 | 0 | 0 | 0 | 0 | 0 | 0 |
| RIENLNKKADDGFPDTWTYNAELLVPLENERTLDYHDSNVKNLHEKVRSQSKNNA | 0 | 0 | 0 | 0 | 0 | 0 | 0 | 0 | 0 | 0 | 0 | 0 | 0 | 0 | 0 | 1 | 0 | 0 |
| RIENLNKEVDDGFLDIWTYNVELLVLLENERTLDYHDSNVKNLYEKVRSQLKNNA | 0 | 0 | 0 | 0 | 0 | 0 | 0 | 1 | 0 | 0 | 0 | 0 | 0 | 0 | 0 | 0 | 0 | 0 |
| RIENLIKKVDDGFLDIWTYNAELLVLLENERTLDYHDSNVKNLYEKVRSQLKNNA | 0 | 0 | 0 | 0 | 0 | 0 | 0 | 0 | 1 | 0 | 0 | 0 | 0 | 0 | 0 | 0 | 0 | 0 |
| RIENFNKKVDDGFLDIWTYNVELLVLLENERTLDYHDSNVKNLYEKVRSQLKNNA | 0 | 0 | 0 | 0 | 0 | 0 | 1 | 0 | 0 | 0 | 0 | 0 | 0 | 0 | 0 | 0 | 0 | 0 |
| RIEKLNKKVDDGFLDIWTYNAELLVLLENERTLDYHNSNVKNLYEKVRSQLKNNA | 0 | 0 | 0 | 0 | 1 | 0 | 0 | 0 | 0 | 0 | 0 | 0 | 0 | 0 | 0 | 0 | 0 | 0 |
| RIEILNKKVDDGFLDIWTYNAELLVLLENERTLDYHDSNVKNLYEKVRSQLKNNA | 0 | 0 | 0 | 0 | 0 | 0 | 0 | 1 | 0 | 0 | 0 | 0 | 0 | 0 | 0 | 0 | 0 | 0 |
| RIEHLNKKVNDGFLDIWTYNAELLVLLENERTLDYHDSNVKNLYEKVRSQLKNNA | 0 | 0 | 1 | 0 | 0 | 0 | 0 | 0 | 0 | 0 | 0 | 0 | 0 | 0 | 0 | 0 | 0 | 0 |
| RIEDLNKKVDDGFLDIWTYNVELLVLLENERTLDYHDSNVKNLYEKVRSQLKNNA | 0 | 0 | 0 | 0 | 0 | 0 | 1 | 0 | 0 | 0 | 0 | 0 | 0 | 0 | 0 | 0 | 0 | 0 |
| RIEDLNKKVDDGFLDIWTYNAELLVLLENERTLDYHDSNVNNLYEKVRSQLKNNA | 0 | 0 | 0 | 1 | 0 | 0 | 0 | 0 | 0 | 0 | 0 | 0 | 0 | 0 | 0 | 0 | 0 | 0 |
| RIEDLNKKVDDGFLDIWTYNAELLVLLENERTLDYHDSNVKSLYEKVRSQLKNNA | 0 | 0 | 0 | 0 | 1 | 0 | 0 | 0 | 0 | 0 | 0 | 0 | 0 | 0 | 0 | 0 | 0 | 0 |
| RIEDLNKKVDDGFLDIWTYNAELLVLLENERTLDYHDSNVKNLYEKIRSQLKNNA | 0 | 0 | 0 | 0 | 0 | 0 | 0 | 0 | 1 | 0 | 0 | 0 | 0 | 0 | 0 | 0 | 0 | 0 |
| RIEDLNKKVDDGFLDIRTYNAELLVLLENERTLDYHDSNVKNLYEKVRSQLKNNA | 0 | 0 | 0 | 0 | 0 | 0 | 1 | 0 | 0 | 0 | 0 | 0 | 0 | 0 | 0 | 0 | 0 | 0 |
| RIDNLNKKVNDGFLDIWTYNAELLVLLENERTLDYHDSNVKNLYEKVRSQLKNNA | 0 | 0 | 0 | 0 | 0 | 0 | 0 | 1 | 0 | 0 | 0 | 0 | 0 | 0 | 0 | 0 | 0 | 0 |
| RIDNLNKKVDDGFLDIWTYNAELLVPLENERTLDYHDSNVKNLYEKVRSQLKNNA | 0 | 0 | 0 | 0 | 0 | 0 | 0 | 0 | 0 | 1 | 0 | 0 | 0 | 0 | 0 | 0 | 0 | 0 |
| GIENLNKKVNDGFLDIWTYNAELLVLLENERTLDYHDSNVKNLYEKVRNQLKNNA | 0 | 0 | 0 | 0 | 0 | 0 | 1 | 0 | 0 | 0 | 0 | 0 | 0 | 0 | 0 | 0 | 0 | 0 |
| GIENLNKKVDDSFLDIWTYNAELLVLLENERTLDYHDSNVKNLYEKVRSQLKNNA | 0 | 0 | 0 | 0 | 0 | 0 | 0 | 0 | 1 | 0 | 0 | 0 | 0 | 0 | 0 | 0 | 0 | 0 |
| GIENLNKKVDDGFLDIWTYNAELLVLLGNERTLDYHDSNVKNLYEKVRSQLKNNA | 0 | 0 | 0 | 0 | 0 | 0 | 0 | 0 | 0 | 0 | 0 | 0 | 0 | 0 | 0 | 0 | 1 | 0 |
| GIENLNKKVDDGFLDIWTYNAELLVLLENERTLGYHDSNVKNLYEKVRSQLKNNA | 0 | 0 | 0 | 0 | 0 | 0 | 0 | 1 | 0 | 0 | 0 | 0 | 0 | 0 | 0 | 0 | 0 | 0 |
| GIENLNKKVDDGFLDIWTYNAELLVLLENERTLDYHDSNVKNLYERVRSQLKNNA | 0 | 0 | 0 | 0 | 1 | 0 | 0 | 0 | 0 | 0 | 0 | 0 | 0 | 0 | 0 | 0 | 0 | 0 |
| GIENLNKKVDDGFLDIWTYNAELLVLLENERTLDYHDSNVKNLYEKVRSQLKNSA | 0 | 0 | 0 | 0 | 0 | 0 | 0 | 1 | 0 | 0 | 0 | 0 | 0 | 0 | 0 | 0 | 0 | 0 |
| GIENLNKKVDDGFLDIWTYNAELLVLLENERTLDYHDSNVKNLYEKIRSQLKNNA | 0 | 0 | 0 | 0 | 0 | 0 | 0 | 0 | 1 | 0 | 0 | 0 | 0 | 0 | 0 | 0 | 0 | 0 |
| GIENLNKKVDDGFLDIWTYNAELLVLLENERTLDYHDSNVKNLCEKVRSQLKNNA | 0 | 0 | 0 | 0 | 0 | 0 | 0 | 1 | 0 | 0 | 0 | 0 | 0 | 0 | 0 | 0 | 0 | 0 |
| GIENLNKKVDDGFLDIWTYNAELLVLLENEGTLDYHDSNVKNLYEKVRSQLKNNA | 0 | 0 | 0 | 0 | 1 | 0 | 0 | 0 | 0 | 0 | 0 | 0 | 0 | 0 | 0 | 0 | 0 | 0 |
| GIENLNKKVDDGFLDIWAYNAELLVLLENERTLDYHDSNVKNLYEKVRSQLKNNA | 0 | 0 | 0 | 0 | 0 | 0 | 1 | 0 | 0 | 0 | 0 | 0 | 0 | 0 | 0 | 0 | 0 | 0 |
